# KLF5 promotes proliferation, migration, and autophagy-/EMT‑associated molecular changes in lens epithelial cells via transcriptional activation of THBS1 in traumatic cataract

**DOI:** 10.1186/s41065-026-00701-x

**Published:** 2026-06-24

**Authors:** Junjun Han, Tian Wang, Xin Yang, Xiaoli Li

**Affiliations:** Department of Ophthalmology, Henan Provincial People’s Hospital, Henan Eye Hospital, Henan Eye Institute, People’s Hospital of Zhengzhou University, No. 7, Weiwu Road, Jinshui District, Zhengzhou City, Henan 450000 China

**Keywords:** Traumatic cataract, Lens epithelial cells, Thrombospondin 1 (THBS1), Krüppel-like factor 5 (KLF5), Proliferation, Migration, Autophagy-related markers, Epithelial-mesenchymal transition

## Abstract

**Purpose:**

Traumatic cataract is a common blinding eye disease after ocular trauma, and its pathogenesis is closely related to lens epithelial cell dysfunction, while the definite molecular regulatory mechanism between upstream transcription factor and downstream target gene remains poorly clarified. This study aimed to clarify the role and molecular mechanism of the krüppel-like factor 5 (KLF5)/ thrombosponin 1 (THBS1) axis in regulating proliferation, migration, epithelial-mesenchymal transition and autophagy of lens epithelial cells in traumatic cataract, and to explore its potential clinical therapeutic value.

**Methods:**

The GSE295383 dataset in the gene expression omnibus (GEO) database was downloaded, and the differentially expressed genes (DEGs) were screened by linear models for microarray data (limma) package of R language. Combined with Weighted gene co-expression network analysis (WGCNA), the gene co-expression network was constructed and the key modules were screened. Gene ontology (GO), kyoto encyclopedia of genes and genomes (KEGG) and gene set enrichment analysis (GSEA) combined with human transcription factor target (hTFtarget) and JASPAR databases were used to predict the upstream transcription factors of THBS1. Subsequently, SRA01/04 cells were induced with transforming growth factor-beta 2 (TGF-β2) to construct a cataract cell model.

**Results:**

THBS1 and KLF5 were highly expressed in LECs exposed to TGF-β2. KLF5 could activate THBS1 transcription by binding to THBS1 promoter − 174 to -165 sites. Knockdown of THBS1 inhibited TGF-β2-induced viability, proliferation, migration, and altered the expression of epithelial-mesenchymal transition (EMT)- and autophagy-related markers in LECs. Knockdown of KLF5 downregulated THBS1 expression and produced a similar inhibitory effect, while overexpression of THBS1 reversed the effect of KLF5 knockdown.

**Conclusions:**

This study demonstrated that KLF5 promoted the proliferation, migration, and EMT‑associated molecular changes of LECs in traumatic cataract through transcriptional activation of THBS1, and regulated the expression of autophagy‑related markers in LECs, suggesting that KLF5/THBS1 axis might be a potential target for the treatment of traumatic cataract.

**Supplementary Information:**

The online version contains supplementary material available at https://doi.org/10.1186/s41065-026-00701-x.

## Introduction

Traumatic cataract stands as a leading cause of vision impairment following ocular trauma. It constitutes a substantial share of blinding ocular conditions globally, particularly among adolescents and manual laborers, significantly compromising both the patients’ quality of life and social productivity [1]. The degree of transparency exhibited by the lens is contingent upon the delicate equilibrium maintained between the homeostatic capabilities of lens epithelial cells (LECs) and the surrounding lens microenvironment [2]. Ocular trauma has the potential to disrupt the lens’s inherent physiological barrier, setting off a localized inflammatory reaction and cellular stress, which can instigate a cascade of pathological alterations in LECs [3]. These pathological manifestations can compromise both the structural integrity and transparency of the lens [4]. Specifically, an excessive proliferation of LECs can readily result in cellular aggregation, while aberrant migratory capabilities can facilitate the accumulation of cells in the central or subcapsular regions of the lens [5]. The process of epithelial-mesenchymal transition (EMT) causes LECs to relinquish their epithelial traits, acquire mesenchymal cell markers, and secrete copious amounts of extracellular matrix [6]. This transformation ultimately culminates in lens opacity and irreversible impairment of vision. Currently, the primary clinical approach for treating traumatic cataract involves surgically excising the opaque lens and implanting an intraocular lens [7]. Nevertheless, this procedure still carries the potential risks of postoperative complications, including posterior capsular opacification, increased intraocular pressure, and inflammatory responses [8]. Hence, gaining a profound comprehension of the fundamental molecular mechanisms underlying the aberrant pathological behaviors of LECs in traumatic cataract, and uncovering the pivotal regulatory factors and signaling pathways, has emerged as a critical imperative for devising targeted therapeutic strategies and enhancing the prognostic outcomes for patients.

In recent years, bioinformatics is widely used to explore disease pathological mechanisms and biomarkers, and Weighted Gene Co-expression Network Analysis (WGCNA) has demonstrated significant promise in unraveling the molecular underpinnings of complex diseases [9, 10]. Through the construction of a gene co-expression network, it becomes feasible to pinpoint crucial gene modules that exhibit a strong correlation with disease phenotypes [11, 12]. Additionally, this approach enables the identification of core regulatory factors along with their potential target genes, thereby offering precise guidance for subsequent mechanistic validation [13, 14]. In the realm of cataract research [15], while certain studies have successfully utilized WGCNA to pinpoint gene modules associated with cataract, a pressing challenge still lies ahead: how to further refine and identify core genes of functional significance from these modules, as well as to clarify their underlying molecular regulatory mechanisms.

Previous research has demonstrated that the regulatory network comprising transcription factors and their target genes serves as a pivotal mechanism in governing the pathological behavior of cells [16]. Thrombospondin-1 (THBS1), a multifunctional protein within the extracellular matrix, has been shown to participate in critical biological processes including cell adhesion, signal transduction, and fibrotic process [17]. Notably, aberrant expression of THBS1 has been observed in various ocular diseases, such as age-related cataract and diabetic retinopathy [18, 19]. Krüppel-like factor 5 (KLF5), a transcription factor of the zinc finger structural family, regulates gene transcription by binding to the promoter regions of target genes. This binding subsequently modulates key cellular processes such as cell proliferation and differentiation [20, 21]. KLF5 has been confirmed by a number of studies to be highly expressed in a variety of diseases, such as diabetic nephropathy and nasopharyngeal carcinoma, and is involved in the regulation of EMT and autophagy progression [22–24]. Most importantly, the study by Li et al. confirmed that KLF5 promotes oxidative stress and EMT in LECs by regulating MDM2 in diabetic cataract [25]. Notably, up to now, no existing literature has confirmed the direct transcriptional binding and regulatory relationship between KLF5 and THBS1 in any pathological model, and the role of the KLF5/THBS1 axis in traumatic cataract remains completely unreported. Accordingly, this study aimed to screen core genes related to traumatic cataract by bioinformatics analysis, explore the biological function of THBS1 in lens epithelial cell proliferation, migration, autophagy and EMT, and further clarify the transcriptional regulatory mechanism between KLF5 and THBS1. The findings were expected to offer new insights into the molecular pathogenesis and promising therapeutic targets for traumatic cataract.

Most importantly, it is necessary to strictly distinguish traumatic cataract from complicated cataract represented by diabetic cataract in clinical and basic research. Traumatic cataract is triggered by definite ocular blunt or penetrating trauma, with acute lens opacity and local ocular inflammatory response after trauma. Diabetic complicated cataract is a metabolic cataract secondary to long-term hyperglycemia, characterized by slow progressive lens snowflake-like opacity, accompanied by diabetic microvascular lesions, and no clear history of ocular trauma. In this study, all clinical dataset samples excluded patients with diabetes, uveitis and other risk factors for complicated cataract, and only enrolled subjects with a clear history of ocular trauma, to ensure that the research object was single and targeted at traumatic cataract.

## Materials and methods

### Construction of gene co-expression network

The GSE295383 dataset was downloaded from the gene expression omnibus (GEO) database (https://www.ncbi.nlm.nih.gov/geo/). It contained 34 lens capsule samples, including 17 non-cataract donor samples (13 males and 4 females) and 17 postoperative cataract donor samples (6 males and 11 females). Detailed individual exact age information was not released in the original GEO dataset; therefore, only gender distribution and sample grouping were presented in this study. In the GSE295383 dataset, enrolled lens capsule samples were strictly defined as traumatic cataract with definite ocular trauma history; patients with diabetes, autoimmune uveitis, and other systemic or ocular diseases leading to complicated cataract were excluded to eliminate the confounding effect of complicated cataract on subsequent bioinformatics analysis and mechanism exploration. The R language was applied to process the sample expression profile data, and the limma package was used to screen for differentially expressed genes (DEGs). Benjamini-Hochberg (BH) method was used for multiple testing correction to control false discovery rate (FDR). The threshold for DEGs was set as |log fold change (logFC)| > 1.5 and BH-adjusted *P* < 0.05.The WGCNA algorithm was used to construct the gene co-expression network, and the top 6000 genes with the highest variance were selected for analysis. In the WGCNA analysis, the soft-thresholding power (β) was calculated using the pickSoftThreshold function. The minimum power achieving scale-free topology fit with R^2^ = 0.85 was selected as β = 26. Network type was set to signed during soft threshold evaluation; the subsequent blockwiseModules construction adopted the default unsigned network with TOMType = “unsigned”. The minimum module size was set as minModuleSize = 30. According to the gene correlation, a hierarchical clustering dendrogram was constructed to identify gene modules, and the correlation between modules and the cataract phenotype was analyzed to screen the key modules. Moreover, the correlation between gene significance (GS) and module membership (MM) was presented by scatter plots.

### Bioinformatics analysis

DEGs in key modules were visualized by volcano plots and heatmap. Cataract-related genes in Venn diagram were obtained from the GeneCards website (https://www.genecards.org/). In addition, the prediction of THBS1’s transcription factor was performed in the human transcription factor target (hTFtarget) database (https://guolab.wchscu.cn/hTFtarget/#!/). The first 2000 bp of the THBS1 promoter was obtained from the UCSC website (https://genome.ucsc.edu/), and then transcription factor binding site information was determined through the JASPAR database (https://jaspar.elixir.no). All online databases were accessed and data downloaded on August 29, 2025, with official URLs provided above.

### Gene Ontology (GO) and Kyoto Encyclopedia of Gene and Genomes (KEGG) enrichment analyses

The “clusterProfiler” package in the R language and DAVID database (https://david.ncifcrf.gov/tools.jsp) were used to carry out GO enrichment analysis (adj. *P* < 0.05) and KEGG pathway enrichment analysis (adj. *P* < 0.05) for the genes in the Venn diagram intersection part, to deeply explore the biological functions of these genes and the related pathways involved. Among them, GO analysis covers three components: biological process (BP), cellular component (CC) and molecular function (MF).

### Gene Set Enrichment Analysis (GSEA)

GSEA can rank genes in gene tables based on phenotypic correlation and evaluate the distribution trend of genes in predefined gene sets to determine the contribution of these genes to a particular phenotype. In this study, GSEA was performed on the entire ranked transcriptome background using the clusterProfiler software package instead of a limited subset of intersection genes to screen out the biological signaling pathways related to cataract phenotype.

### Cell culture and treatment

The human lens epithelial cell (LEC) line SRA01/04 was purchased from Procell (Wuhan, China). The growth medium for these cells consisted of 20% fetal bovine serum (Thermo Fisher Scientific, Rockville, MD, USA), 1% penicillin/streptomycin (Invitrogen, Carlsbad, CA, USA), and DMEM medium (high glucose) (Thermo Fisher Scientific, ). The culture conditions were constant temperature 37℃, containing 95% air and 5% CO_2_.

TGF-β2 was used to establish the cell model because ocular trauma can trigger local inflammation and upregulate TGF-β2 in the lens microenvironment, which recapitulates the key pathological phenotypes of LEC proliferation, migration, EMT, and autophagy during traumatic cataract development. To mimic the pathological state of LECs in cataract, SRA01/04 cells were treated with recombinant human transforming growth factor-beta 2 (TGF-β2) (1 µg/mL) (Yeasen, Shanghai, China) for 12 h to establish an in vitro cell model of cataract. In this study, TGF-β2 induction was applied for two purposes: firstly, to establish a stable in vitro cell model mimicking the pathological changes of lens epithelial cells under traumatic cataract microenvironment; secondly, this model was used to validate the transcriptomic differential expression trends of KLF5 and THBS1 screened from GEO bioinformatics analysis, and further explore the regulatory mechanism and cellular function of the KLF5/THBS1 axis.

### Western blot

SRA01/04 cells were lysed using RIPA lysis buffer (Beyotime) containing protease inhibitors. Subsequently, the protein concentration was determined utilizing the BCA Protein Assay Kit (Beyotime). Following separation via SDS-PAGE, the proteins were transferred onto PVDF membranes (Millipore, Billerica, MA, USA). The membranes were blocked with a solution of 5% skim milk/TBST for 1 h, and were then incubated with primary antibodies overnight at 4℃ and secondary antibody Goat Anti-Rabbit IgG H&L (HRP) (1:5000, ab6721, Abcam) for 1 h at room temperature. Protein bands were visualized using RapidStep ECL Reagent (Millipore Corp.) and imaged with a chemiluminescence system. Band intensities were quantified using ImageJ software. All target protein levels were normalized to the corresponding loading control (GAPDH) before statistical analysis. Primary antibodies used for Western blot assay included Anti-Thrombospondin 1 antibody (1:1000, ab263905, Abcam, Cambridge, UK), Anti-LC3B antibody (1:1000, ab192890, Abcam), Anti-Beclin 1 antibody (1:1000, ab207612, Abcam), Anti-SQSTM1/p62 antibody (1:1000, ab109012, Abcam), Anti-APG5L/ATG5 antibody (1:1000, ab108327, Abcam), Anti-Collagen I (COL-1) antibody (1:1000, ab138492, Abcam), Anti-Fibronectin antibody (1:1000, ab2413, Abcam), Anti-alpha smooth muscle actin (α-SMA) antibody (1:1000, ab124964, Abcam), Anti-E Cadherin antibody (E-cad) (1:1000, ab40772, Abcam), Anti-KLF5 antibody (1:1000, ab137676, Abcam), anti-PI3K (1:1000, 4257, Cell Signaling Technology (CST), Danvers, Massachusetts, USA), anti- phospho-PI3K (1:1000, 4228, CST), anti-AKT (1:1000, 4691, CST), anti-phospho-AKT (1:1000, 4060, CST), and Anti-GAPDH antibody (1:10000, ab181602, Abcam).

### Cell transfection

Short hairpin RNAs (shRNAs) targeting THBS1 (sh-THBS1) and KLF5 (sh-KLF5) (GeneChem, Shanghai, China) were used to achieve loss of function of THBS and KLF5 in SRA01/04 cells, and non-targeted sh-NC was used as the control group. As for the overexpression of THBS1, the empty pcDNA3.1 vector (Invitrogen) was used as the control. The THBS1 sequence was cloned into the pcDNA3.1 vector to construct the THBS1 overexpression vector (oe-THBS1). Finally, sh-THBS1, sh-KLF5 or oe-THBS1 was transfected into SRA01/04 cells using Lipofectamine 2000 (Invitrogen). The transfection process was carried out strictly in accordance with the manufacturer’s instructions.

### Assay of cell viability

Cell viability was measured using the Cell Counting Kit-8 (CCK-8) (Yeasen). For TGF-β dose-response analysis, lens epithelial cells were treated with TGF-β at concentrations of 0, 0.1, 0.5, 1, and 2 µg/mL for 24 h. Cell viability was measured using the CCK-8 assay according to the manufacturer’s instructions.

In particular, SRA01/04 cells were inoculated into 96-well plates with a cell density of 1 × 10^4^ cells per well. To reduce edge effects, the peripheral wells were filled with PBS. After 24 h of incubation in an incubator, the corresponding TGF-β2 and transfection treatments were performed. 3 compound holes were set in each group. After 48 h, 10 µL of CCK8 reagent was introduced into each well, followed by gentle mixing. The plates were then placed back in the incubator for 3 h. Subsequently, the optical density (OD) values were determined at a wavelength of 450 nm utilizing a microplate reader.

### Assay of cell proliferation

Monitoring of the proliferative behavior of SRA01/04 cells was achieved using the BeyoClick™ EdU-594 Cell Proliferation Assay kit (C0078L, Beyotime). The specific operation steps were carried out in strict accordance with the manufacturer’s instructions.

### Assay of cell migration

Cell migration was determined by Transwell and wound healing assays. The procedure of Transwell assay was as follows: SRA01/04 cells were collected and adjusted to 1 × 10^5^ cells/mL with serum-free DMEM medium, and 500 µL of complete medium was added to the lower chamber of 24-well plate. Then, the Transwell chamber (Corning, Tewksbury, MA, USA) was placed into the lower chamber and 200 µL of the cell suspension was added, and the cells were incubated for 12 h in an incubator. Subsequently, the surface of the upper chamber filter membrane was gently rubbed with a sterile cotton swab, and the chamber membrane was placed face down into a well containing 400 µL 4% paraformaldehyde (Beyotime) and fixed at room temperature (30 min). Next, the chambers were then transferred to wells containing 0.1% crystal violet (Solarbio) staining solution and stained at room temperature in the dark (20 min). After washing with PBS to remove excess staining solution, the purple-stained cells on the lower surface of the filter membrane were observed under a microscope and counted.

For the scratch assay, SRA01/04 cells were seeded into a 6-well plate, and when confluence reached 90%, a straight line of uniform width was drawn vertically into the 6-well plate with a 10 µL sterile pipette tip. Care should be taken to keep the width of the scratches in each group basically the same. The cell surface was washed twice with PBS, and photographs were taken to record the scratch width (0 h). After 24 h of incubation in the incubator, photographs were taken again to record the wound width. Images were imported into Image J software, and wound width was quantified to assess cell migration ability.

### Chromatin immunoprecipitation (ChIP) assay

SRA01/04 cells underwent cross-linking treatment with formaldehyde. Cross-linked chromatin was then isolated and sheared into small fragments by sonication. These chromatin fragments were then incubated with either anti-KLF5 antibody (ab277773, Abcam) or IgG (ab172730, Abcam) as a negative control overnight at 4℃. Subsequently, protein A/G beads (Beyotime) were introduced to facilitate the capture of the antibody-protein-DNA complex. These beads were then subjected to multiple rounds of washing to effectively eliminate any non-specific binding. Once the step of reversing cross-linking was finished, the pertinent DNA fragments underwent purification. Ultimately, qPCR was employed to quantify the enrichment of the THBS1 promoter region in the immunoprecipitated DNA.

### Dual-luciferase reporter assay

SRA01/04 cells that had been transfected with sh-NC or sh-KLF5 were seeded into 24-well culture plates. When the cells had grown to a density of about 70%, wild-type THBS1 promoter firefly luciferase reporter plasmid (THBS1-WT) or the luciferase reporter plasmid with a mutation at the binding site of the THBS1 promoter (THBS1-MUT) was co-transfected into the cells with the pRL-TK plasmid (which expresses renilla luciferase) as an internal control. Following transfection, the cells were incubated for a period of 48 h, after which they were lysed. The activities of both firefly luciferase and renilla luciferase within the cell lysates were then measured sequentially, utilizing the dual-luciferase reporter system (Promega). Finally, the relative luciferase activity was determined by normalizing the firefly luciferase activity to the renilla luciferase activity.

### Statistical analysis

All experiments were performed with at least 3 biological replicates. Data were analyzed with the use of GraphPad Prism 9.0 software, and results were presented as mean ± standard deviation. Prior to parametric statistical analysis, the Shapiro–Wilk test was used to verify the normality of data distribution, and Levene’s test was applied to assess the homogeneity of variance. All experimental data followed a normal distribution and exhibited homogeneous variance. Student’s *t*-test was used to analyze the differences between the two groups. Differences between multiple groups were analyzed by one-way or two-way analysis of variance (ANOVA) with Tukey’s post hoc test. *P* < 0.05 indicated a significant difference between the data.

## Results

### WGCNA identified key gene modules associated with cataract

**Fig. 1:**
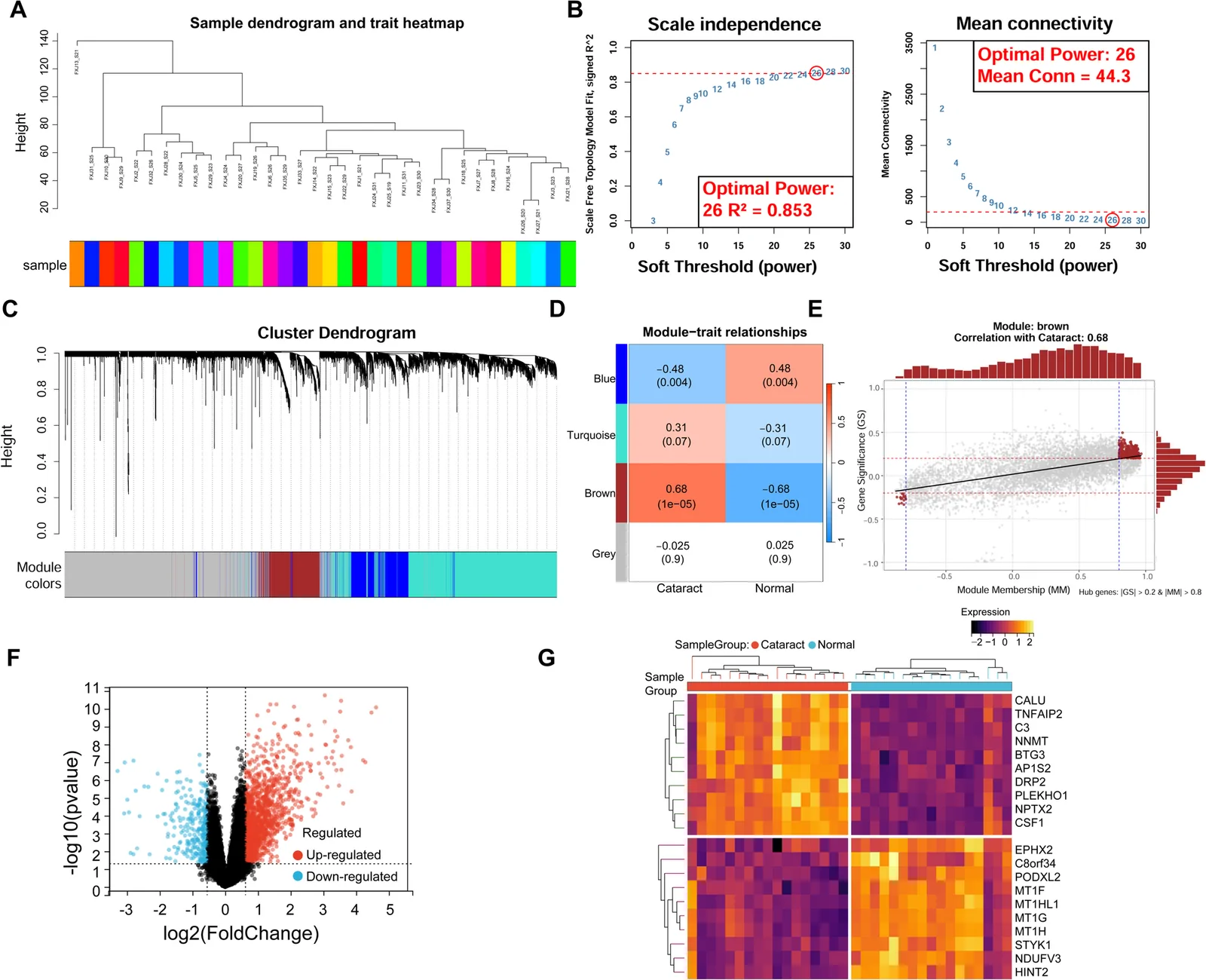
Construction of the WGCNA co-expression network. **A** Sample clustering dendrogram with tree leaves corresponding to individual samples. **B** The scale independence and average connectivity under different soft thresholds were predicted, with 26 selected as the optimal soft threshold for constructing the gene co-expression network. The scale-free R2 was 0.853. **C** The clustering dendrogram of genes showed, there were 4 modules. **D** Module-trait correlation heatmap to reveal the strength of association between each module and trait (Cataract or Normal). **E** The correlation between MM and GS in the “Brown” module was displayed via a scatter plot. **F** Volcano plot of DEGs between Cataract and Normal samples. **G** Hierarchical clustering heatmap of top DEGs

To precisely identify the central genes closely associated with the phenotype of cataract, this study employed the WGCNA algorithm to construct a gene co-expression network. The expression profiles of the GSE295383 dataset were analyzed using the limma function in the R language software. The sample hierarchical clustering analysis indicated that the clustering effect among the samples was good, and no significant outliers were found (Fig. 1A). Furthermore, the scale-free topological indices under different soft threshold powers were evaluated. Finally, a power of 26 was selected (with R^2^ = 0.853 at this point), and under this power, the network could approximately achieve a scale-free topological structure. At the same time, an analysis was conducted on the trend of average connectivity changes during the process of increasing the soft threshold power. It was found that when the power was 26, the average connectivity tended to stabilize, which was consistent with the aforementioned selection results (Fig. 1B). Based on the optimized power, a gene clustering dendrogram was constructed according to the similarity of gene co-expression. Through the dynamic tree-cutting method and subsequent merging operations on highly similar modules, the genes were separated into different modules, and there was a clear separation between the modules and the features (Fig. 1C). 4 distinct gene modules were successfully identified. By analyzing the correlations between each gene module and the phenotype (cataract or normal samples), it was found that the “Brown” module (cor = 0.68, *P* = 1e − 05) exhibited the most representative characteristics (Fig. 1D). The scatter plot further revealed the high correlation between GS (|GS|>0.2) and module MM (|GS|>0.8) in the “Brown” module (Fig. 1E). Subsequently, a volcano plot was used to comprehensively display the overall distribution of differentially expressed genes (DEGs) in the “Brown” module, including both upregulated and downregulated genes (Fig. 1F). The heatmap also provided detailed information on the top 10 upregulated and downregulated genes (Fig. 1G). In conclusion, through the WGCNA algorithm, the genes were successfully clustered into modules closely related to the characteristics, laying a solid foundation for in-depth exploration of the key gene modules in cataract.

### Screening of hub genes by GO, KEGG, and GSEA enrichment analysis

**Fig. 2:**
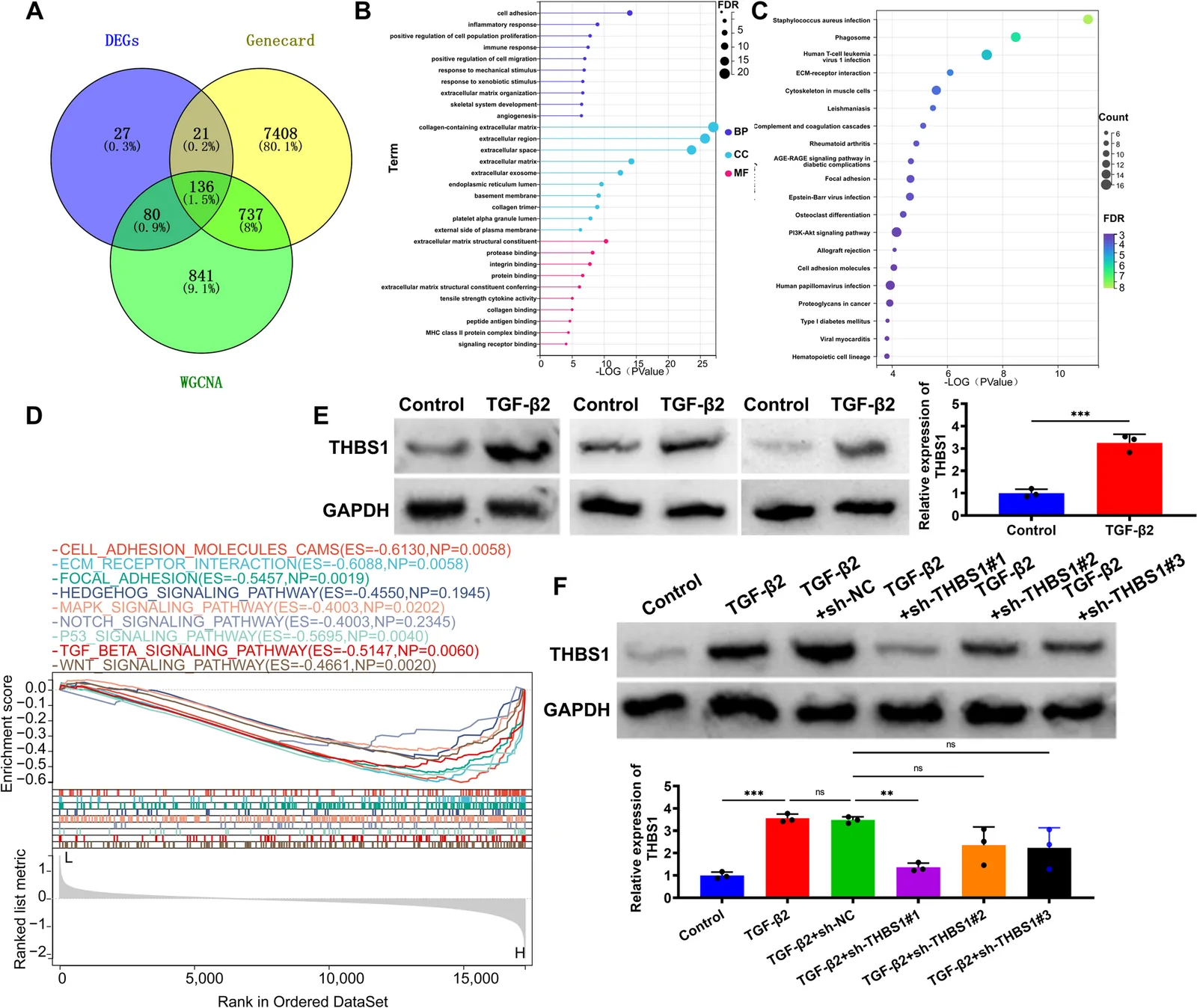
Screening of hub gene. **A** A Venn diagram among DEGs derived from Fig. 1F (|logFC|og.5), cataract-related genes in the GeneCards database, and genes included in the “Brown” module of WGCNA. **B**-**D** GO, KEGG, and GSEA enrichment analysis of genes in the Venn diagram intersection set. **E** After LECs were treated with TGF-β2 (1 µg/mL) for 12 h, the protein level of THBS1 was determined by Western blot using untreated SRA01/04 cells as the Control group (n = 3 biological replicates). **F** SRA01/04 cells exposed to TGF-β2 were transfected with sh-NC, sh-THBS1#1, sh-THBS1#2 or sh-THBS1#3. The knockdown efficiency of THBS1 was determined by Western blot (n = 3 biological replicates). ns, not significant; **, *P* < 0.01; ***, *P* < 0.001

To further screen the hub genes, first, a Venn diagram was used to display the intersection of 264 DEGs selected from the heatmap in Fig. 1F and 8302 cataract-related genes from the GeneCards database, and 1794 genes in the “Brown” module of the WGCNA algorithm. The result was shown in Fig. 2A. There were 136 common genes in the intersection. The GO functional enrichment analysis indicated that the intersected genes were mainly enriched in cell adhesion, positive regulation of cell population proliferation, and positive regulation of cell migration, and were related to the structural components of the extracellular matrix (Fig. 2B). The KEGG pathway enrichment analysis revealed that the intersecting genes were significantly enriched in the PI3K-AKT signaling pathway (*P* < 0.05). Among them, the THBS1 gene exhibited the maximum absolute value of log fold change (logFC = 3.8) among all intersected candidate genes, with significantly high expression in cataract tissues (*P* < 0.05). This prominent differential expression pattern, combined with its enrichment in cataract-related functional pathways, made THBS1 stand out as the most prioritized key candidate gene for subsequent mechanistic validation (Fig. 2C). GSEA analysis results showed that the intersecting genes were significantly enriched in CELL_ADHESION_MOLECULES_CAMS, ECM_RECEPTOR_INTERACTION, FOCAL_ADHESION, HEDGEHOG_SIGNALING_PATHWAY, MAPK_SIGNALING_PATHWAY, NOTCH_SIGNALING_PATHWAY, P53_SIGNALING_PATHWAY, TGF_BETA_SIGNALING_PATHWAY, and WNT_SIGNALING_PATHWAY, and all of these pathways were closely related to the EMT process (Fig. 2D). To determine the optimal working concentration of TGF-β for our in vitro model, we performed a dose-response cell viability assay (Supplementary Fig. 1B). Compared with the 0 µg/mL control group, cell viability was significantly increased at concentrations of 0.5, 1, and 2 µg/mL. Cell viability reached a plateau at 1 µg/mL, with no significant difference observed between 1 µg/mL and 2 µg/mL. Based on these results, 1 µg/mL TGF-β was selected as the optimal concentration for all subsequent functional experiments, as it induced the maximal pro-proliferative effect without reaching a non-specific plateau. Then, it was found that after treating LECs with TGF-β2, the protein level of THBS1 was obviously upregulated (Fig. 2E). After that, the knockdown efficiency of 3 shRNAs targeting THBS1 was detected, and the results presented that sh-THBS1#1 could significantly inhibit the protein expression of THBS1 in SRA01/04 cells exposed to TGF-β2, so sh-THBS1#1 was selected (Fig. 2F). Collectively, THBS1 was identified as a hub gene and was likely to be closely related to cell migration, proliferation, and the EMT process.

### Knockdown of THBS1 curbed the viability, proliferation and migration of SRA01/04 cells treated with TGF-β2

**Fig. 3:**
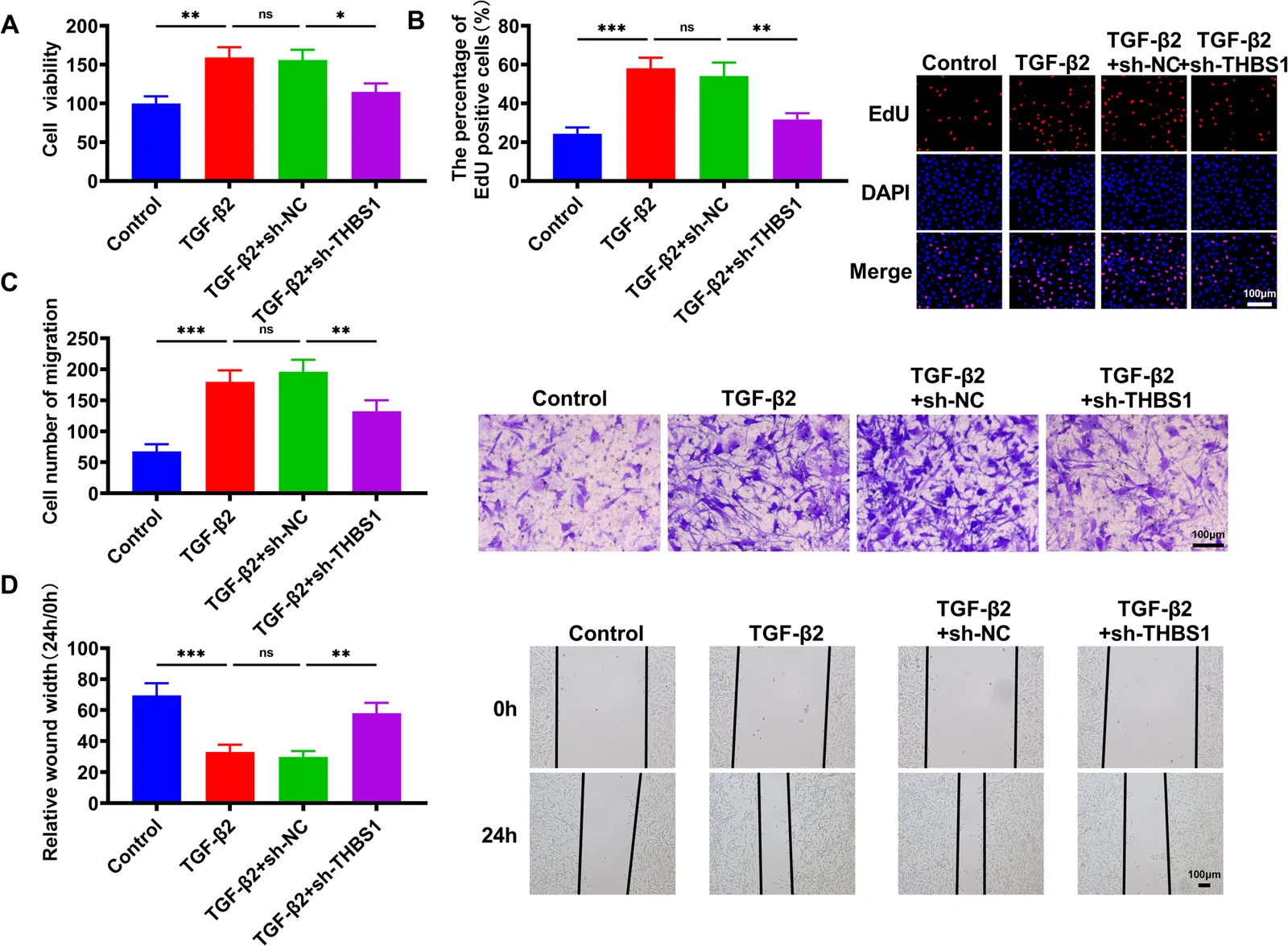
Influence of silencing THBS1 on the viability, proliferation and migration of SRA01/04 cells exposed to TGF-β2. SRA01/04 cells were divided into 4 groups: the Control group, TGF-β2 group, TGF-β2 + sh-NC group, and TGF-β2 + sh-THBS1 group. **A** The viability of each group of cells was determined using the CCK-8 kit (n = 3 biological replicates). **B** Monitoring of cell proliferation behavior using EdU method (Scale bar: 100 μm) (n = 3 biological replicates). **C** and **D**) The migration ability of SRA01/04 cells was evaluated by using Transwell and scratch assays (Scale bar: 100 μm) (n = 3 biological replicates). ns, not significant; *, *P* < 0.05; **, *P* < 0.01; ***, *P* < 0.001

To determine the role played by THBS1 in TGF-β2-exposed LECs, cell viability, proliferation, and migration were analyzed after THBS1 knockdown. CCK-8 and EdU assays showed that the viability of SRA01/04 cells was enhanced and the number of proliferating cells was increased after treatment with TGF-β2. However, when THBS1 expression was impeded, cell viability and proliferation were also decreased (Figs. 3A and B). In the Transwell experiment of Fig. 3C, it could be observed that TGF-β2 caused a significant increase in the number of cells migrating to the lower chamber. Knockdown of THBS1 evidently inhibited cell migration. Meanwhile, the results of Transwell assay were further verified by scratch assay. Compared with the Control group, the wound width of SRA01/04 cells in the TGF-β2 group was significantly reduced, while the wound width of TGF-β2 + sh-THBS1 group was larger than that of TGF-β2 + sh-NC group (Fig. 3D). The above results indicated that THBS1 knockdown could remarkably restrain the enhanced cell viability, proliferation, and migration ability of SRA01/04 cells in response to TGF-β2.

### Knockdown of THBS1 reversed TGF-β2-induced alterations in autophagy/EMT-related markers in SRA01/04 cells

**Fig. 4:**
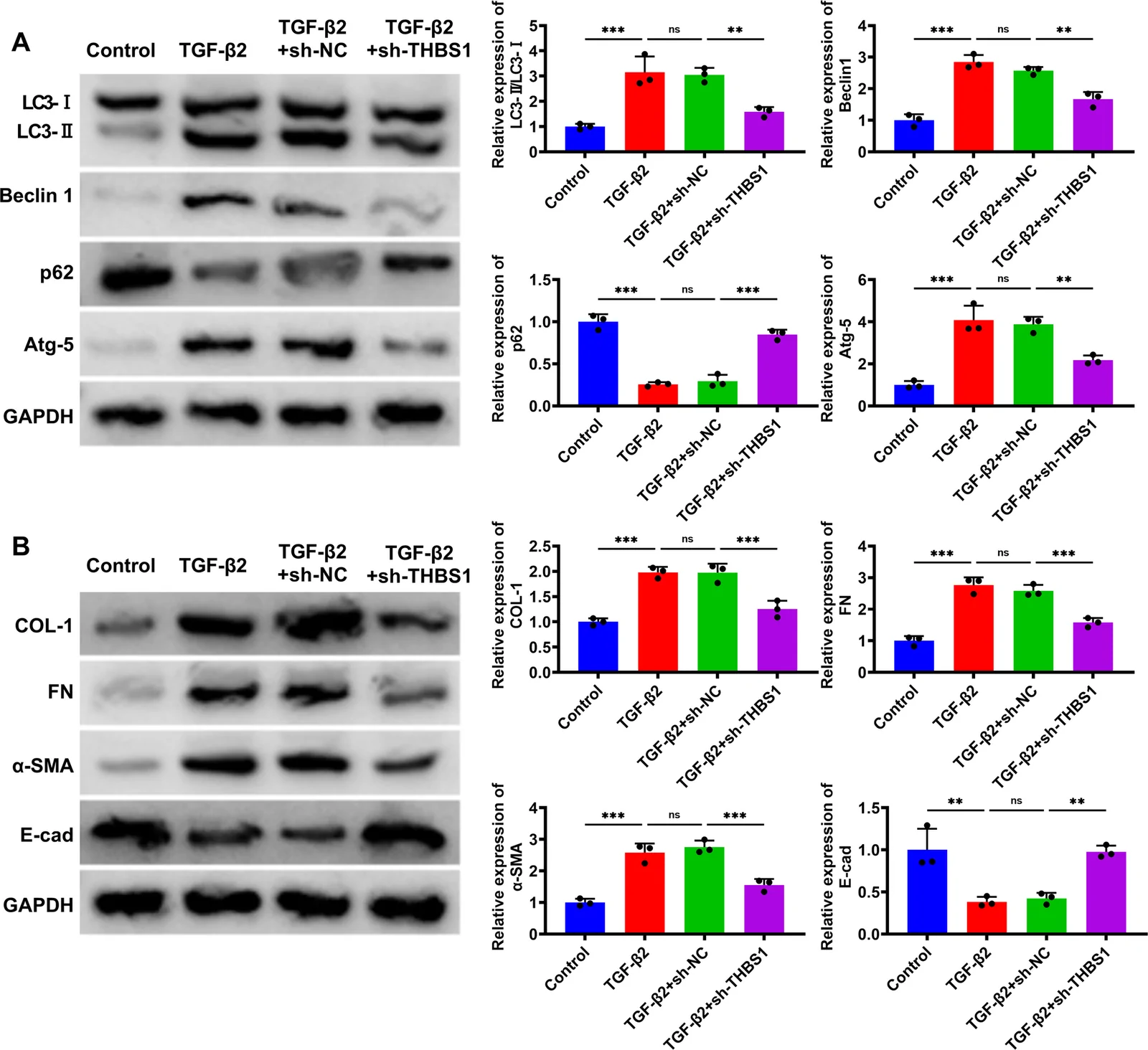
Effect of silencing THBS1 on autophagy and EMT-related markers. SRA01/04 cells were divided into 4 groups: the Control group, TGF-β2 group, TGF-β2 + sh-NC group, and TGF-β2 + sh-THBS1 group. **A** The changes in the levels of autophagy-related proteins (LC3-I, LC3-II, Beclin 1, p62, and Atg-5) in LECs exposed to TGF-β2 after silencing THBS1 were detected by Western blot (n = 3 biological replicates). **B** The influence of silencing THBS1 on the expression of EMT-related proteins (COL-1, FN, α-SMA, and E-cad) in SRA01/04 cells exposed to TGF-β2 was measured by Western blot (n = 3 biological replicates). ns, not significant; **, *P* < 0.01; ***, *P* < 0.001

Notably, although autophagy was not the dominant pathway in the initial genomic enrichment, traumatic cataract-induced cellular stress can robustly activate LEC autophagy, which closely interacts with EMT and participates in cataract pathogenesis [26–28]. For this reason, this part included autophagy-related molecular detection to comprehensively characterize the regulatory effect of the KLF5/THBS1 axis on the complete pathological phenotypes of LECs. The effect of knockdown of THBS1 on the expression of autophagy-related and EMT-related proteins was examined by Western blot. LC3B, Beclin1, ATG5 and p62 are classic autophagy-related markers that reflect the initiation and flux of autophagic process, and their abnormal expression is closely associated with cataract-related pathological changes in LECs. Figure 4A displayed that TGF-β2 induced alterations in the expression of autophagy-related markers in LECs, as evidenced by an increased LC3-I to LC3-II transition, upregulated levels of Beclin 1 and Atg-5, and downregulated p62 expression. Silencing of THBS1 reversed these TGF-β2-induced changes in autophagy-related protein expression, with decreased relative expression of LC3-II/LC3-I, Beclin 1, and Atg-5, and increased p62 protein level. E-cad is a core epithelial marker, while COL-1, FN and α-SMA are typical mesenchymal markers; the expression shift of these proteins is the key characteristic of EMT in LECs during traumatic cataract progression. Obviously, the protein expression of COL-1, FN, and α-SMA was markedly increased, while the protein level of E-cad was downregulated in SRA01/04 cells exposed to TGF-β2. Likewise, knockdown of THBS1 reversed the effect of TGF-β2 on the protein levels of COL-1, FN, α-SMA, and E-cad (Fig. 4B). In summary, TGF-β2 induced alterations in autophagy/EMT-related markers in LECs, and inhibiting the expression of THBS1 could effectively reverse these changes in autophagy/EMT-related markers.

### KLF5 transcriptionally activated THBS1 expression in LECs

**Fig. 5:**
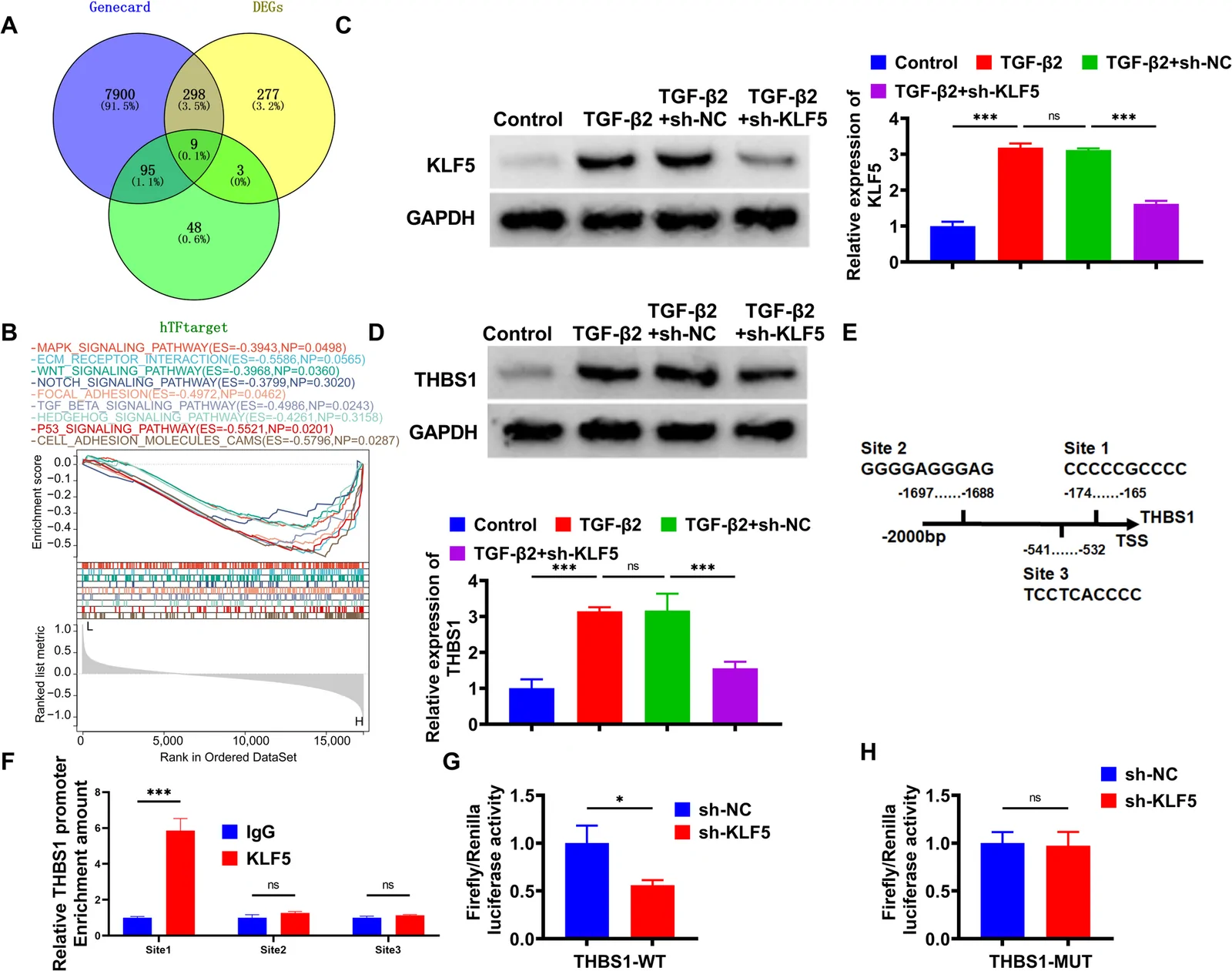
KLF5 transcriptionally activates THBS1 expression in SRA01/04 cells. **A** A Venn diagram among DEGs derived from Fig. 1F (|logFC|ogF, cataract-related genes in the GeneCards database, and transcription factors of THBS1 predicted via the hTFtarget database. **B** GSEA enrichment analysis of genes in the Venn diagram intersection set. **C** Western blot was used to detect the protein level of KLF5 in LECs exposed to TGF-β2 and the knockdown efficiency of KLF5 after transfection with sh-KLF5 (n = 3 biological replicates). **D** SRA01/04 cells were divided into 4 groups: the Control group, TGF-β2 group, TGF-β2 + sh-NC group, and TGF-β2 + sh-KLF5 group. The effect of KLF5 knockdown on THBS1 protein levels was determined by Western blot (n = 3 biological replicates). **E** The UCSC website was used to select the first 2000 base pairs of the THBS1 gene promoter region, and the binding sites of KLF5 factors were determined with the help of the JASPAR database. **F** ChIP assay was performed to assess the binding of KLF5 to the THBS1 promoter (n = 3 biological replicates). **G** and **H** Dual-luciferase reporter assay was used to analyze the transcriptional activities of THBS1-WT and THBS1-MUT reporter genes in SRA01/04 cells transfected with sh-KLF5 or sh-NC (n = 3 biological replicates). ns, not significant; *, *P* < 0.05; ***, *P* < 0.001

To further clarify the molecular mechanism of THBS1 regulating cataract, the focus was placed on the upstream transcription factors of THBS1. A total of 155 transcription factors of THBS1 were predicted from the hTFtarget database, and subsequently 587 DEGs with upregulated expression (|logFC| ≥ 1) were screened based on the volcano plot in Fig. 1F. The intersection of the above two and 8302 cataract-related genes from the GeneCards database was presented by a Venn diagram. The intersection set contained nine genes, namely KLF5, MYC, ERG, RUNX1, RUNX2, MYOD1, CEBPD, CEBPA, and ETS1 (Fig. 5A). GSEA enrichment analysis verified that genes in the intersection were mainly enriched in MAPK_SIGNALING_PATHWAY, ECM_RECEPTOR_INTERACTION, WNT_SIGNALING_PATHWAY, NOTCH_SIGNALING_PATHWAY, FOCAL_ADHESION, TGF_BETA_SIGNALING_PATHWAY, HEDGEHOG_SIGNALING_PATHWAY, P53_SIGNALING_PATHWAY, and CELL_ADHESION_MOLECULES_CAMS (Fig. 5B). Through a systematic review of previous related studies, it was found that only KLF5 showed a high expression state in other diseases and could promote the progression of the diseases [23, 25, 29]. Based on this, this study selected KLF5 as the research object. Similar to THBS1, the protein level of KLF5 was also upregulated in TGF-β2-exposed SRA01/04 cells. After transfection with sh-KLF5, the protein expression of KLF5 was specifically hindered (Fig. 5C). As expected, THBS1 protein expression was also blocked in the presence of sh-KLF5 (Fig. 5D). 3 binding sites of KLF5 on the THBS1 promoter, Site1 (-174~-165, CCCCCGCCCC), Site2 (-1697~-1688, GGGGAGGGAG), and Site3 (-541~-532, TCCTCACCCC), were demonstrated through JASPAR website (Fig. 5E). ChIP assay presented that the KLF5 antibody at Site1 precipitated significantly more THBS1 promoter DNA than the IgG group, while Site2 and Site3 were not significantly different from the IgG group, indicating that KLF5 was able to directly bind Site1 at the THBS1 promoter (Fig. 5F). Furthermore, the enrichment level of THBS1 promoter Site1 in the TGF-β2-treated group was significantly higher than that in the untreated control group, demonstrating that TGF-β2 exposure markedly enhanced the binding of KLF5 to the THBS1 promoter (Supplementary Fig. 2). In the dual-luciferase reporter assay, knockdown of KLF5 substantially decreased the luciferase activity of the THBS1-WT construct. In contrast, no statistically significant alterations were detected in the THBS1-MUT group when compared to the sh-NC group (Figs. 5G and H). In summary, the genomic and bioinformatic analyses successfully identified the brown module as the pivotal gene module associated with traumatic cataract. THBS1 was screened and validated as the central hub gene involved in LEC adhesion, proliferation, migration, and EMT-related signaling cascades. Meanwhile, KLF5 was predicted as a major upstream transcription factor capable of regulating THBS1 transcription, providing a reliable genomic basis for subsequent in vitro mechanistic and functional validation experiments.

### THBS1 overexpression abolished the inhibitory effect of KLF5 knockdown on the viability, proliferation, migration, and autophagy/EMT related markers of SRA01/04 cells induced by TGF-β2

**Fig. 6:**
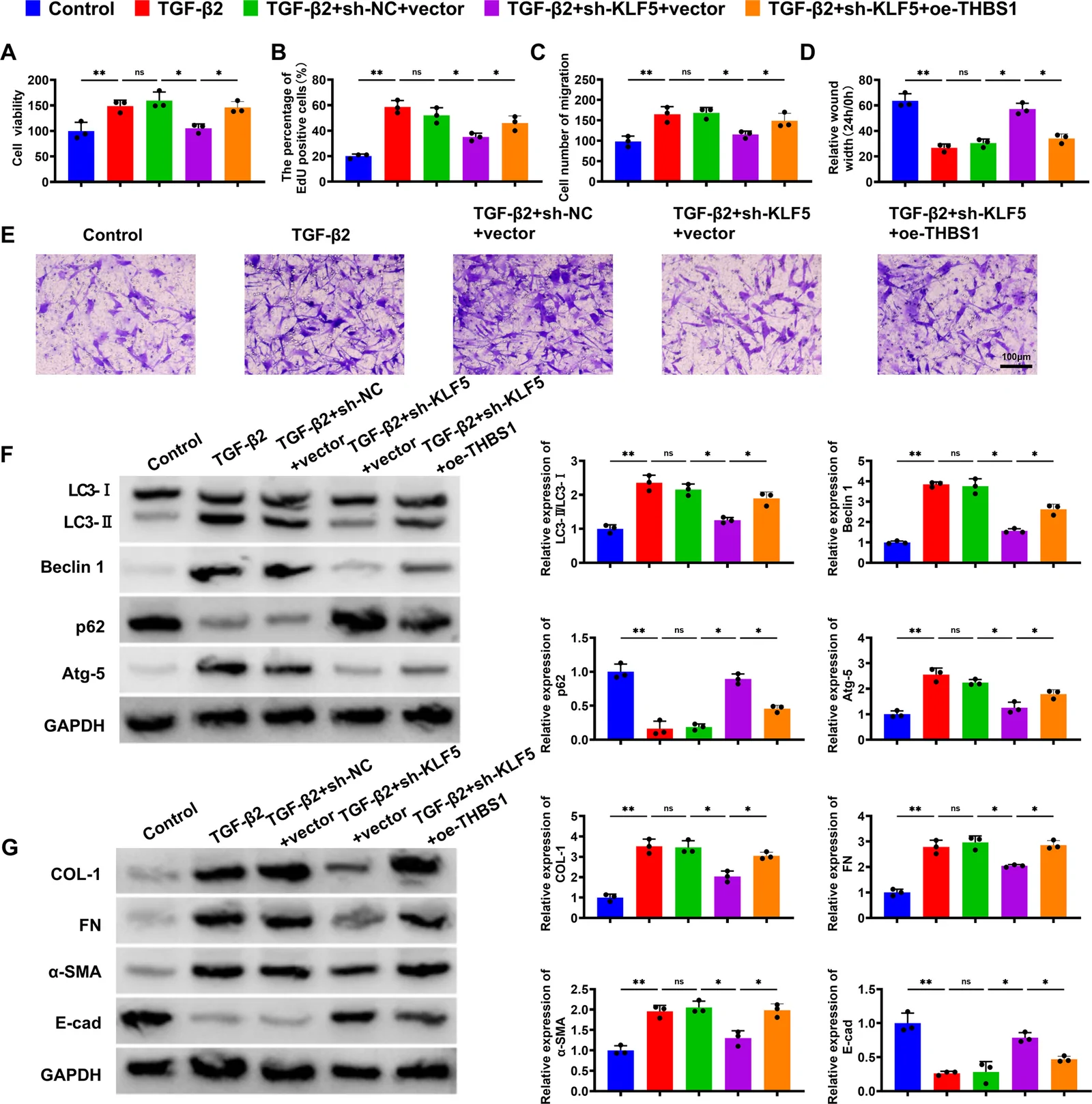
THBS1 overexpression reverses the effects of KLF5 knockdown on the viability, proliferation, migration, and autophagy- and EMT-related markers of SRA01/04 cells exposed to TGF-β2. SRA01/04 cells were divided into 5 groups: the Control group, TGF-β2 group, TGF-β2 + sh-NC+vector group, TGF-β2 + sh-KLF5 + vector group, and TGF-β2 + sh-KLF5 + oe-THBS1 group. **A** and **B** Analysis of cell viability and proliferation by CCK-8 and EdU assays (n = 3 biological replicates). **C** and **D **Detection of cell migration was performed via Transwell and scratch assay (n = 3 biological replicates). **E** Images of cells migrating to the lower chamber of a Transwell plate under a light microscope (Scale bar: 100 μm) (n = 3 biological replicates). **F** and **G** Monitoring of autophagy-related (LC3-I, LC3-II, Beclin 1, p62, and Atg-5) and EMT-related (COL-1, FN, α-SMA, and E-cad) proteins by Western blot (n = 3 biological replicates). ns, not significant; *, *P* < 0.05; **, *P* < 0.01

Rescue experiments were performed to verify the regulatory role of the KLF5/THBS1 axis in SRA01/04 cells exposed to TGF-β2. The results of cell viability, proliferation and migration assays in Fig. 6A-E can confirm that knockdown of KLF5 notably repressed the increased cell viability, proliferation, and migration ability due to TGF-β2 treatment. However, these inhibitory effects were completely abolished when THBS1 was overexpressed. Knockdown of KLF5 reversed TGF-β2-induced alterations in autophagy/EMT-related markers in LECs, consistent with the effects of THBS1 knockdown. Similarly, the effects of KLF5 knockdown on autophagy-related (LC3-I, LC3-II, Beclin 1, p62, and Atg-5) (Fig. 6F) and EMT-related (COL-1, FN, α-SMA, and E-cad) (Fig. 6G) proteins were reversed by overexpression of THBS1. The results of the above rescue experiments confirmed that transcriptional activation of THBS1 by KLF5 accelerated the growth and migration of LECs in cataract, regulated the expression of autophagy/EMT-related markers.

### The KLF5/THBS1 axis regulates the activation of PI3K/AKT signaling pathway in TGF-β2-induced LECs

Given that GO/KEGG and GSEA enrichment analysis revealed significant enrichment of the PI3K-AKT signaling pathway in traumatic cataract, we further detected the phosphorylation levels of PI3K and AKT to explore whether PI3K/AKT acts as the downstream signaling cascade of the KLF5/THBS1 axis. As shown in Supplementary Fig. 1A, TGF-β2 treatment obviously upregulated the protein phosphorylation levels of p-PI3K and p-AKT without affecting the total protein levels of PI3K and AKT. Knockdown of KLF5 significantly reduced the phosphorylation of PI3K and AKT induced by TGF-β2. Notably, overexpression of THBS1 largely restored the decreased p-PI3K and p-AKT expression caused by KLF5 knockdown. Collectively, these results demonstrated that the KLF5/THBS1 axis positively modulates the activation of PI3K/AKT signaling pathway in lens epithelial cells under traumatic cataract microenvironment.

## Discussion

Although prior work has linked KLF5 to EMT and cataract pathogenesis, our study substantially advances the field by identifying THBS1 as a novel downstream mediator of KLF5 in traumatic cataract. This study systematically illustrates that the KLF5/THBS1 axis constitutes the core regulatory mechanism underlying LEC pathological abnormalities, in which KLF5 functions as an upstream transcription factor to directly activate THBS1 transcription, thereby mediating LEC proliferation, migration, EMT progression and autophagy alterations. Importantly, it was further demonstrate that KLF5 drives these pathological LEC behaviors through THBS1-dependent activation of the PI3K/AKT signaling pathway, which represents the key innovation and theoretical contribution of the present work.THBS1 was identified as a core hub gene for traumatic cataract through weighted gene co-expression network analysis (WGCNA) combined with GO and KEGG enrichment analysis, and its association with EMT-related signaling pathways was further confirmed by GSEA [30–38]. These EMT-associated pathways are the key regulatory cascades driving LEC phenotypic abnormalities in traumatic cataract, and their activation is closely linked to the loss of epithelial characteristics, enhanced cell migration and extracellular matrix remodeling of LECs—hallmark pathological changes of cataract formation. As a multifunctional extracellular matrix protein, THBS1 has been implicated in the progression of various ocular fibrotic and degenerative diseases, and our in vitro validation confirmed its upregulation in TGF-β2-induced cataract cell models and its pro-pathological role in promoting LEC viability, proliferation and migration. This finding is consistent with previous reports that THBS1 upregulation exacerbates posterior capsule opacification, further supporting its conserved pathogenic function in lens-related ocular disorders. Autophagy serves as a crucial degradation and recycling system within cells, which is essential for maintaining intracellular homeostasis by clearing away damaged or superfluous organelles and proteins [39, 40]. In the context of traumatic cataracts, LECs undergo substantial physical or chemical injury that triggers a host of intracellular stress responses, which is accompanied by abnormal alterations in the expression of autophagy-related markers in LECs [41].

Emerging evidence suggests that changes in autophagic activity may eliminate intracellular stress signaling molecules and thereby facilitate the EMT process, although the specific link between autophagic flux and EMT in traumatic cataract LECs remains to be elucidated [42, 43]. THBS1 is recognized as a multifunctional protein within the extracellular matrix. Prior research has indicated its involvement in promoting pathological processes in a range of fibrotic conditions as well as ocular pathologies [44]. Notably, Chen et al. demonstrated that lncRNA HOTAIR/WTAP contributes to the aggravation of posterior capsule opacification through the upregulation of THBS1 expression [45]. Moreover, it was reported that the transcription factor ATF6 drove SNAI1, thereby stimulating EMT in the fibrotic lesions of cataracts [46]. Besides, the transcription factor FOXO3 has been proven to exacerbate diabetic cataracts by inducing autophagy [47]. KLF5, as a key transcription factor, was predicted and verified to directly bind to the THBS1 promoter and activate its transcription. Both ChIP and dual‑luciferase assays confirmed that KLF5 serves as an upstream regulator of THBS1 in LECs. Herein, it was found that TGF-β2 exposure could induce significant changes in autophagy and EMT-related markers. Inhibiting the KLF5/THBS1 axis could alleviate these changes to some extent. However, it should be noted that the current research results only show the changes in the expression levels of autophagy and EMT-related markers, and are not sufficient to fully confirm that the KLF5/THBS1 axis regulates the autophagy and EMT processes of LECs in traumatic cataracts. Furthermore, with the addition of PI3K/AKT pathway phosphorylation validation, our study is no longer limited to a simple correlative relationship between KLF5/THBS1 and cellular phenotypes, but establishes a clear molecular mechanistic link: KLF5 transcriptionally activates THBS1, further triggers PI3K/AKT signaling pathway activation, and ultimately promotes abnormal proliferation, migration, EMT and autophagy alterations of lens epithelial cells in traumatic cataract. TGF-β2 acts as an upstream pathological inducer in traumatic cataract, and the KLF5/THBS1/PI3K-AKT cascade serves as the core downstream signaling branch mediating TGF-β2-induced LEC pathological changes, which also echoes our previous GSEA enrichment results of the TGF-β signaling pathway.

Although this study preliminarily clarified the regulatory role of the KLF5/THBS1 axis in lens epithelial cells during traumatic cataract pathogenesis, several limitations still remain. First, all in vitro experiments were performed exclusively in the SRA01/04 cell line, and the TGF-β2-induced cell model only mimics inflammatory injury responses after ocular trauma. This in vitro system cannot fully recapitulate the complex mechanical trauma stimulation and dynamic in vivo inflammatory microenvironment of traumatic cataract. Second, all findings are merely validated at the cellular level, without verification in clinical specimens or animal models, which reduces the generalizability and clinical translational value of the present results. Third, autophagy status was indirectly evaluated only by detecting the expression of classical marker proteins including LC3, Beclin1, ATG5, and p62. These static molecular alterations cannot effectively distinguish excessive autophagosome formation from impaired lysosomal degradation, making it difficult to accurately define genuine autophagic activity. Fourth, the assessment of EMT progression was solely based on the expression profiles of epithelial and mesenchymal markers. This study lacked cell morphological observation, detection of key EMT transcription factors such as SNAI1 and ZEB1, and functional verification via cell invasion assays, rendering the current EMT conclusion preliminary. Future research will improve the experimental system in multiple aspects. Autophagic flux assays with lysosomal inhibitor intervention will be adopted to precisely evaluate actual autophagic activity. Cell morphological observation, detection of core EMT transcriptional regulators, and invasion functional experiments will be integrated to further support EMT evidence. In addition, multiple cell lines, animal models and clinical tissue samples will be included for further validation, so as to solidify the role of the KLF5/THBS1 axis in traumatic cataract and provide more reliable experimental basis for potential clinical therapeutic targets.

In essence, based on WGCNA bioinformatics analysis and in vitro cellular experiments, the current study has elucidated an important regulatory role of the KLF5/THBS1 axis in the pathological changes of LECs during the development of traumatic cataract. This finding not only provides a new experimental perspective for understanding the molecular mechanisms underlying LEC dysfunction in traumatic cataract but also establishes a preliminary scientific basis for exploring potential novel therapeutic targets for this disease. Moving forward, future research must delve deeper into the mechanisms of the KLF5/THBS1 axis within an in vivo context, as well as explore other potential regulatory factors and signaling pathways. Such efforts aim to furnish more thorough theoretical backing and practical guidance for the effective treatment of traumatic cataract.

## Supplementary Information

Supplementary Material 1: Figure 1. (A) Western blot analysis of p-PI3K, total PI3K, p-AKT and total AKT protein levels in Control, TGF-β2, TGF-β2 + sh-NC+vector, TGF-β2 + sh-KLF5 + vector, and TGF-β2 + sh-KLF5 + oe-THBS1 groups (*n* = 3 biological replicates). (B) CCK-8 was used to detect the effect of different concentrations (0, 0.1, 0.5, 1, and 2 µg/mL) of TGF-β2 on cell viability (*n* = 3 biological replicates). ns, not significant; *, *P* < 0.05; **, *P* < 0.01; ***, *P* < 0.001.

Supplementary Material 2: Figure 2 ChIP assay were conducted to assess the enrichment level of KLF5 at the THBS1 promoter site 1 in untreated and TGF-β2-treated SRA01/04 cells (n = 3 biological replicates). ***, *P*<0.001.

Supplementary Material 3.

## Data Availability

The datasets used and analysed during the current study are available from the corresponding author on reasonable request.
