# Supplementary material for: KLF5 promotes proliferation, migration, and autophagy-/EMT‑associated molecular changes in lens epithelial cells via transcriptional activation of THBS1 in traumatic cataract
Source: Hereditas. 2026 Jun 24;163:94. doi: 10.1186/s41065-026-00701-x (PMC13555852; doi:10.1186/s41065-026-00701-x)
Supplement: Supplementary file 1 [file 41065_2026_701_MOESM3_ESM.pdf]

Fig2 E

THBS1
180KDA

GAPDH
36KDA

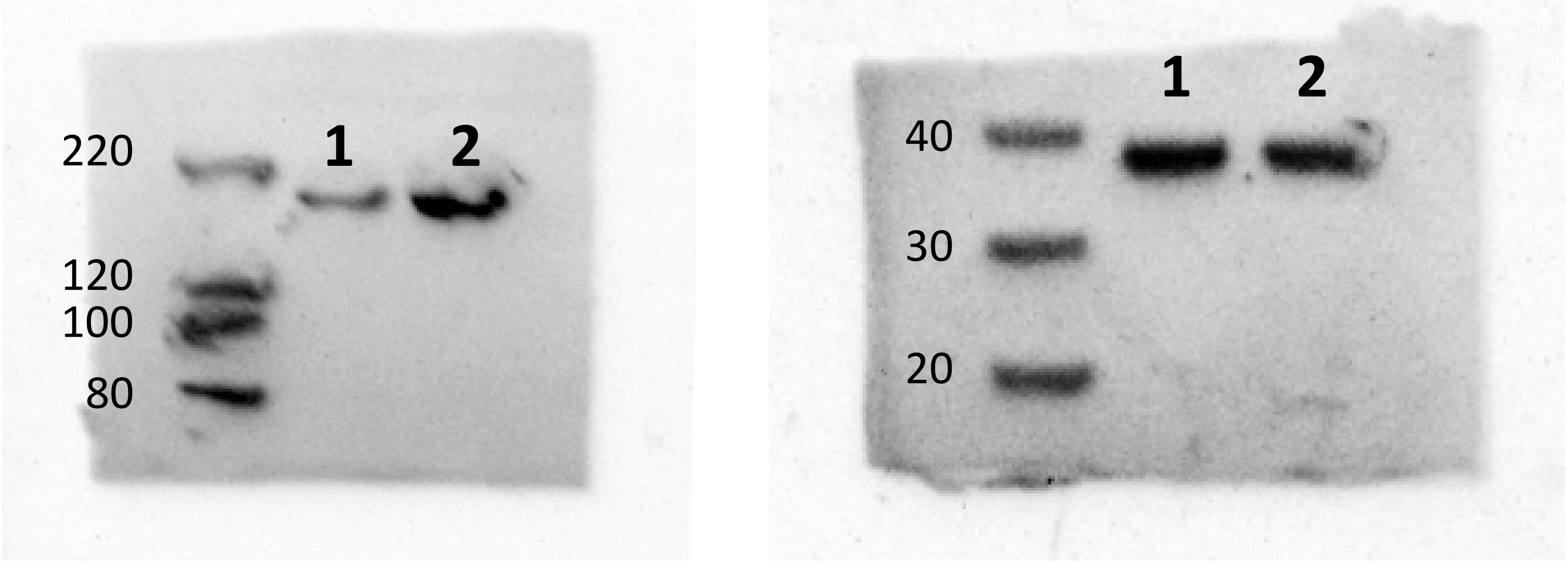

1. Control
2. TGF-β2

Fig2 F

1. Control
2. TGF-β2
3. TGF-β2+sh-NC
4. TGF-β2+sh-THBS1#1
5. TGF-β2+sh-THBS1#2
6. TGF-β2+sh-THBS1#3

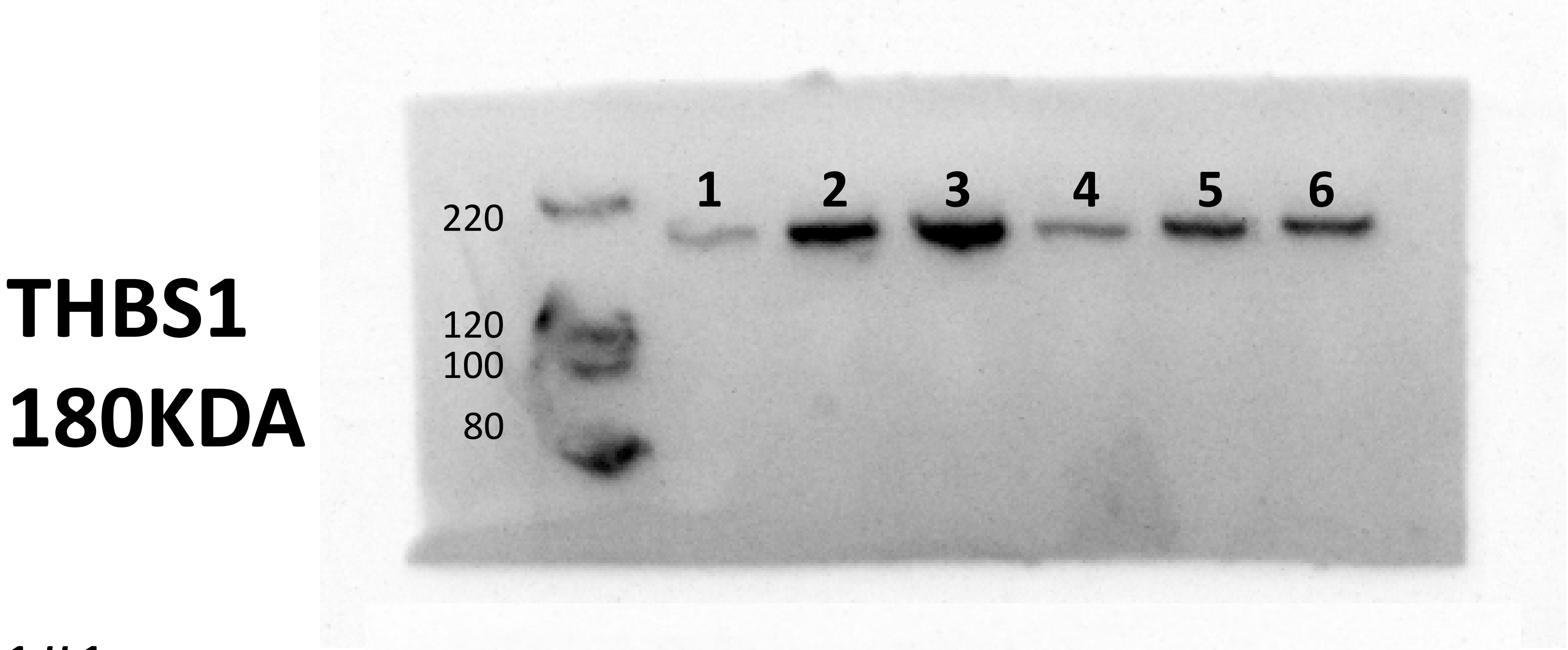

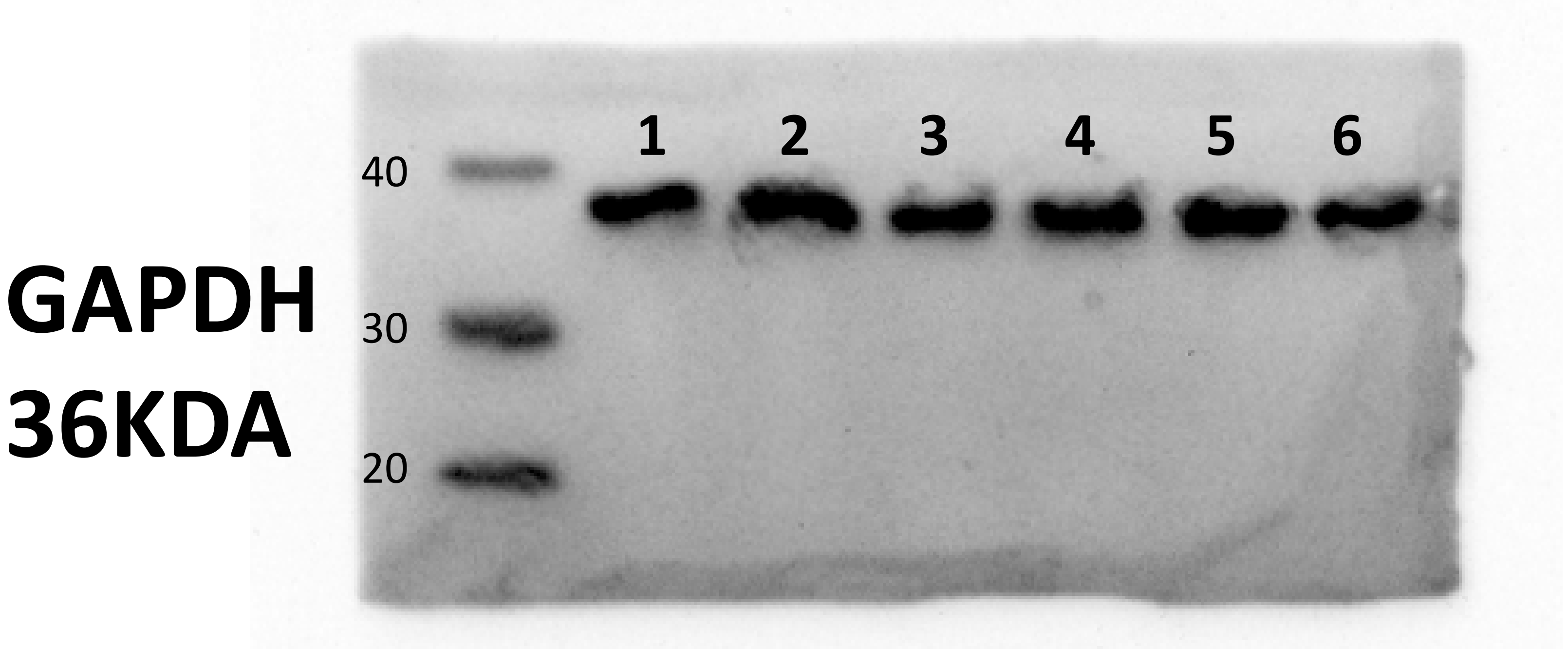

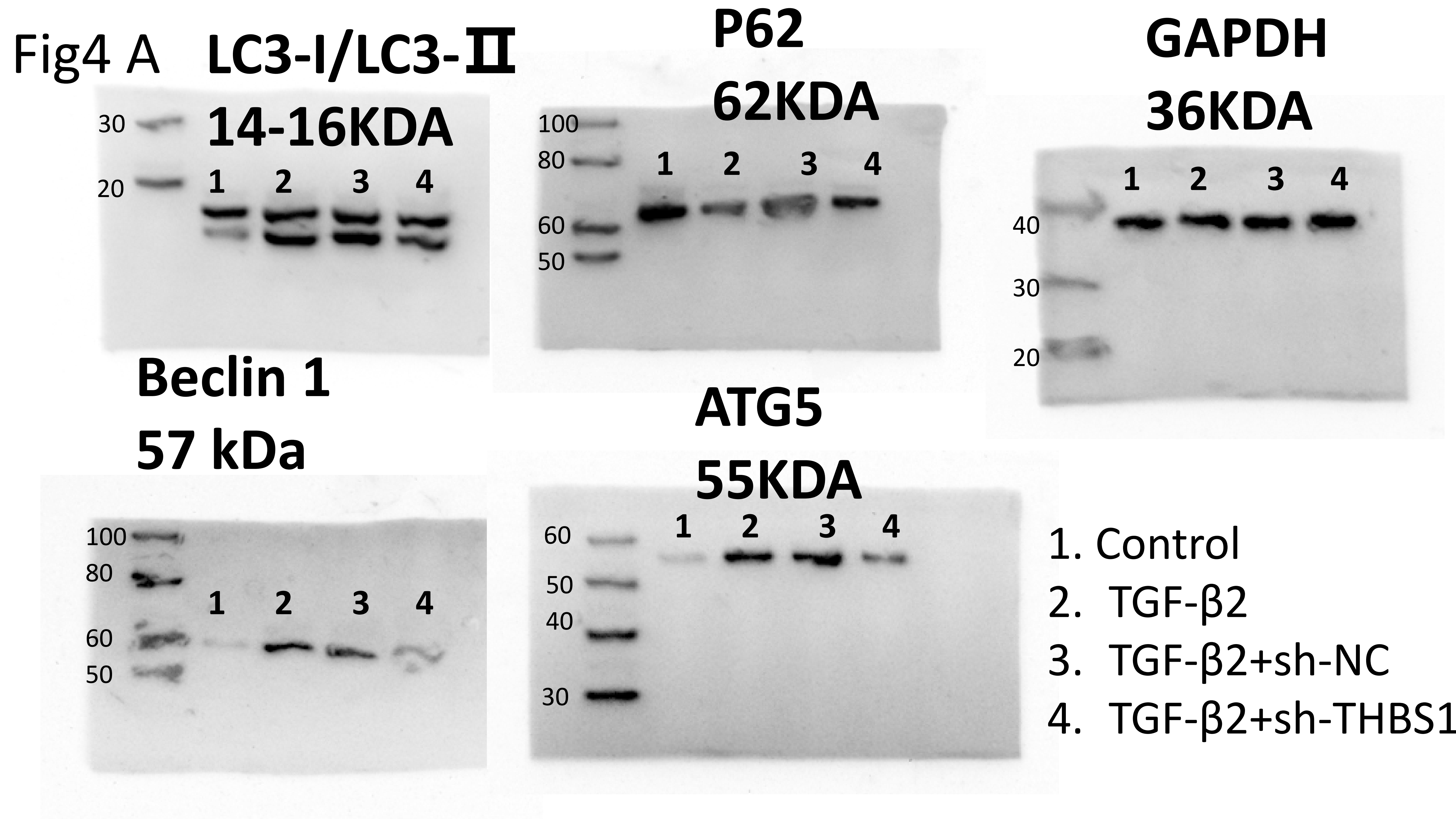
Fig4 A
LC3-I/LC3-Ⅱ
14-16KDA
30
20
1 2 3 4
P62
62KDA
100
80
60
50
1 2 3 4
GAPDH
36KDA
40
30
20
1 2 3 4
Beclin 1
57 kDa
100
80
60
50
1 2 3 4
ATG5
55KDA
60
50
40
30
1 2 3 4
1. Control
2. TGF-β2
3. TGF-β2+sh-NC
4. TGF-β2+sh-THBS1

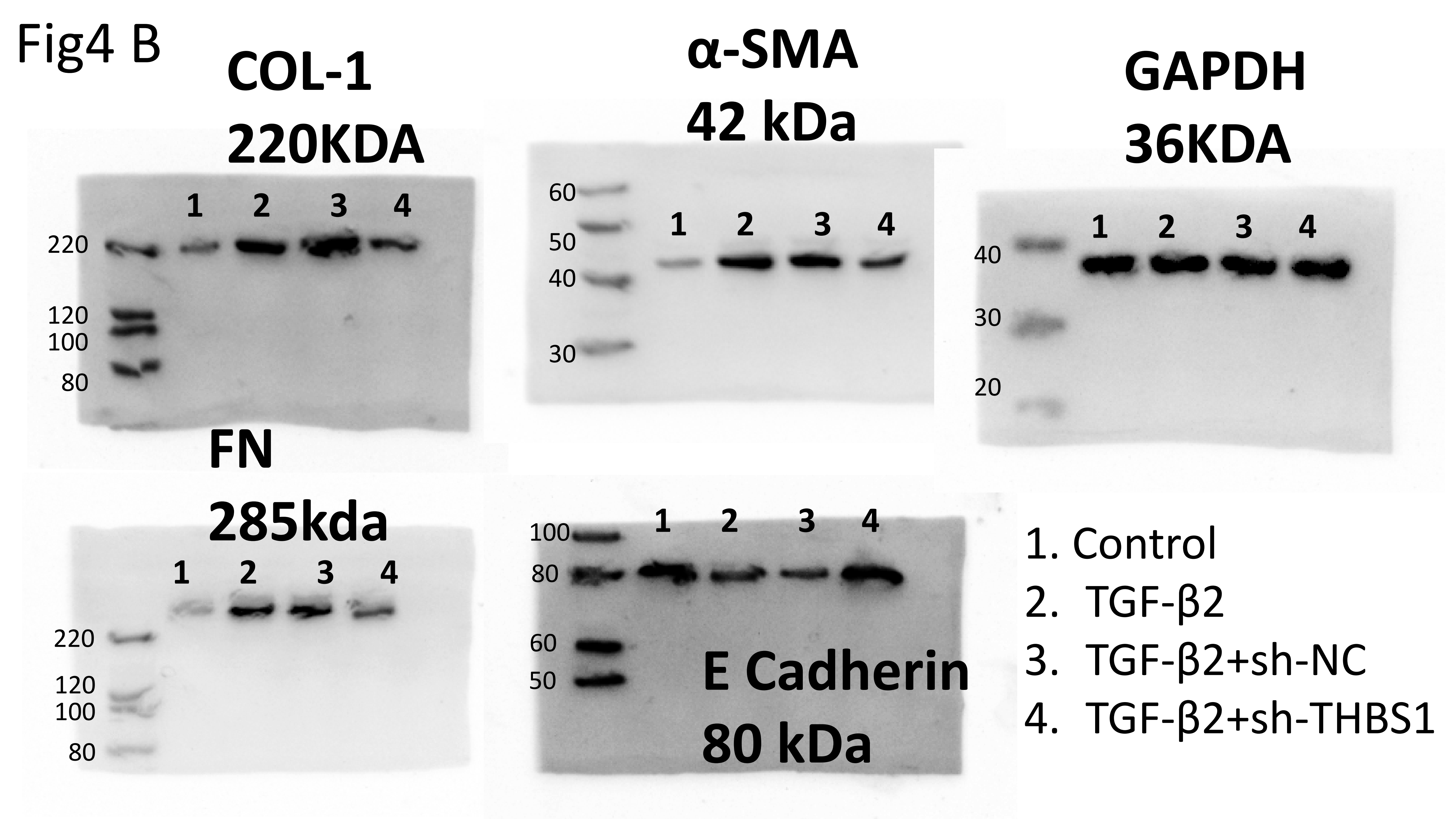
Fig4 B
COL-1
220KDA
1 2 3 4
220
120
100
80
α-SMA
42 kDa
60
50
40
30
1 2 3 4
GAPDH
36KDA
1 2 3 4
40
30
20
FN
285kda
1 2 3 4
220
120
100
80
100
80
60
50
1 2 3 4
E Cadherin
80 kDa
1. Control
2. TGF-β2
3. TGF-β2+sh-NC
4. TGF-β2+sh-THBS1

Fig5 A

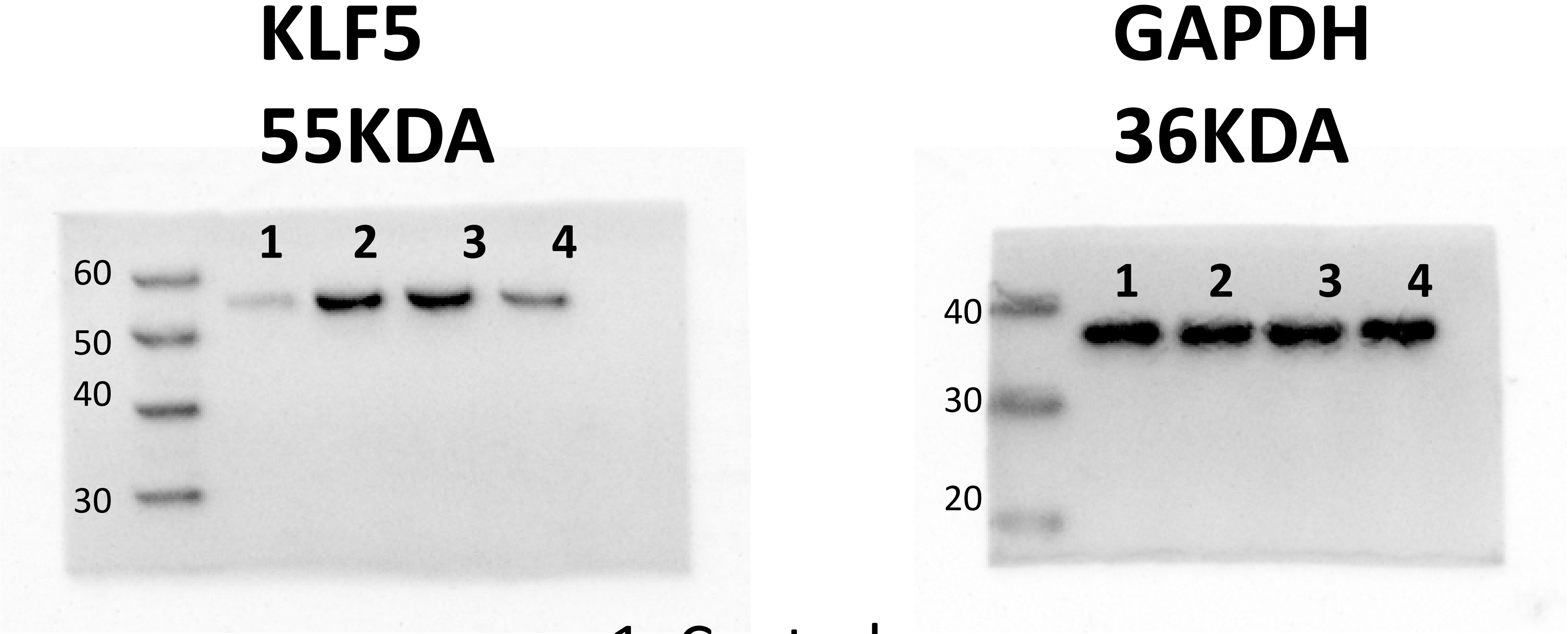

1. Control
2. TGF-β2
3. TGF-β2+sh-NC
4. TGF-β2+sh-KLF5

Fig5 D

THBS1
180KDA

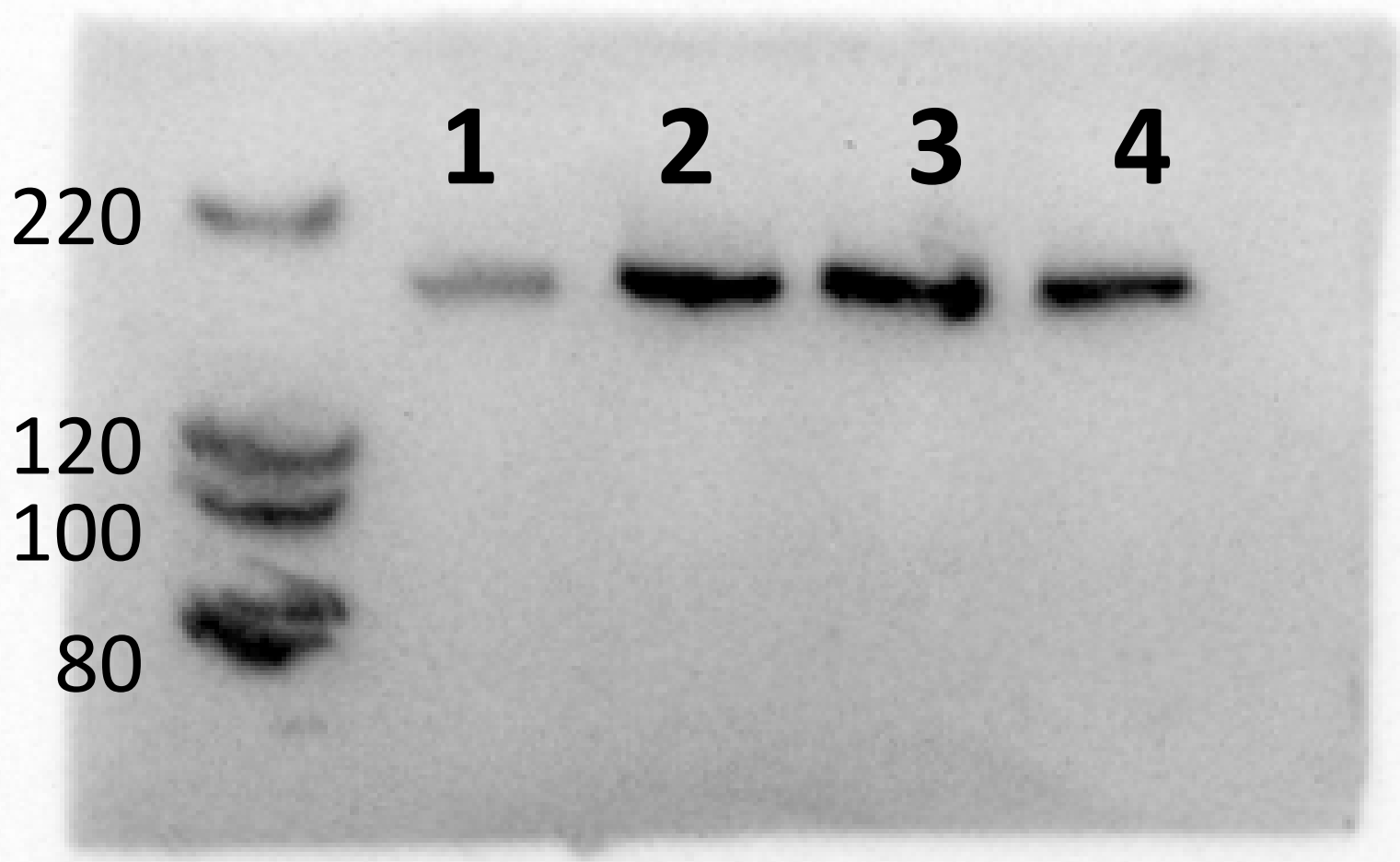

GAPDH
36KDA

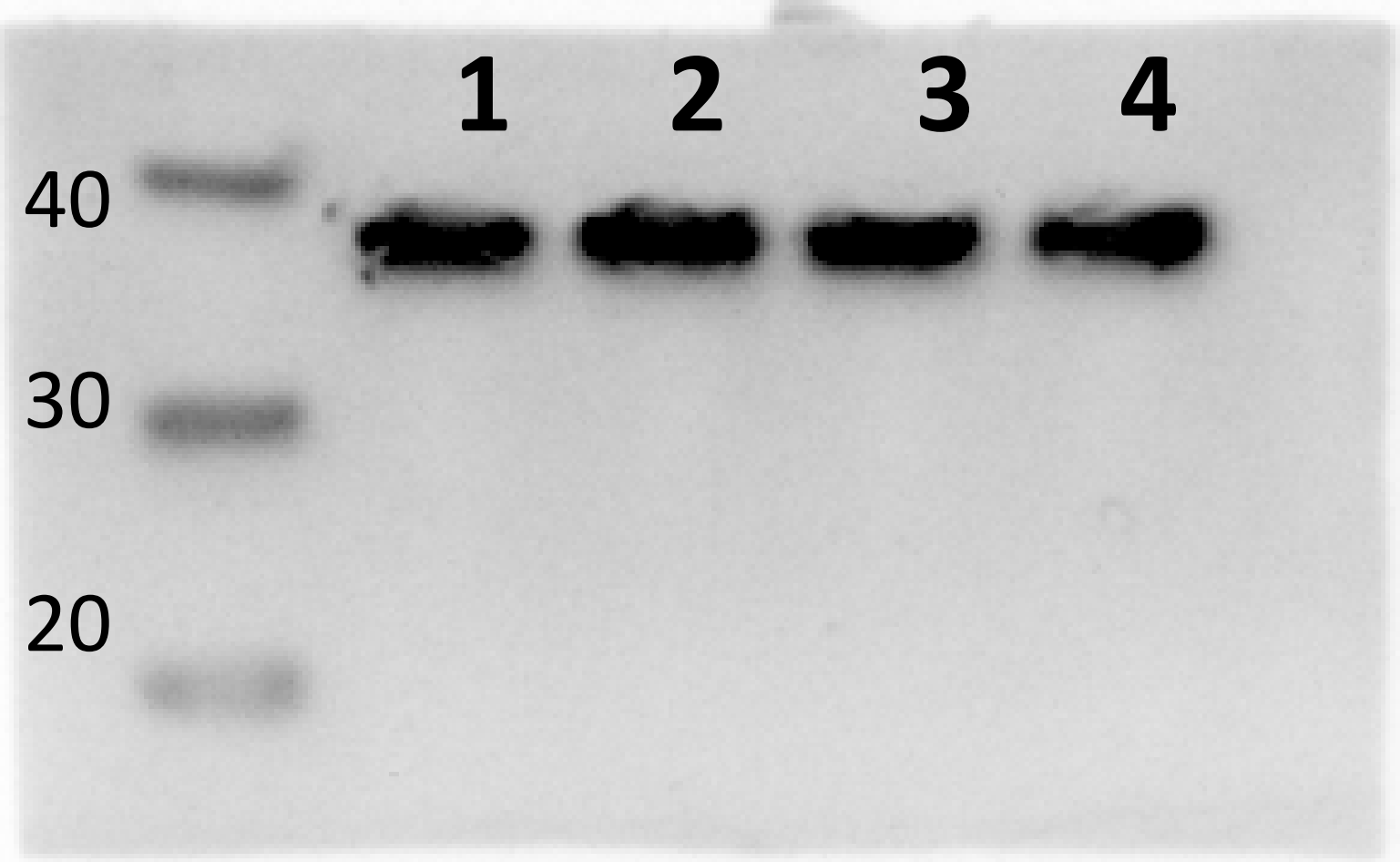

1. Control
2. TGF-β2
3. TGF-β2+sh-NC
4. TGF-β2+sh-KLF5

Fig6 F

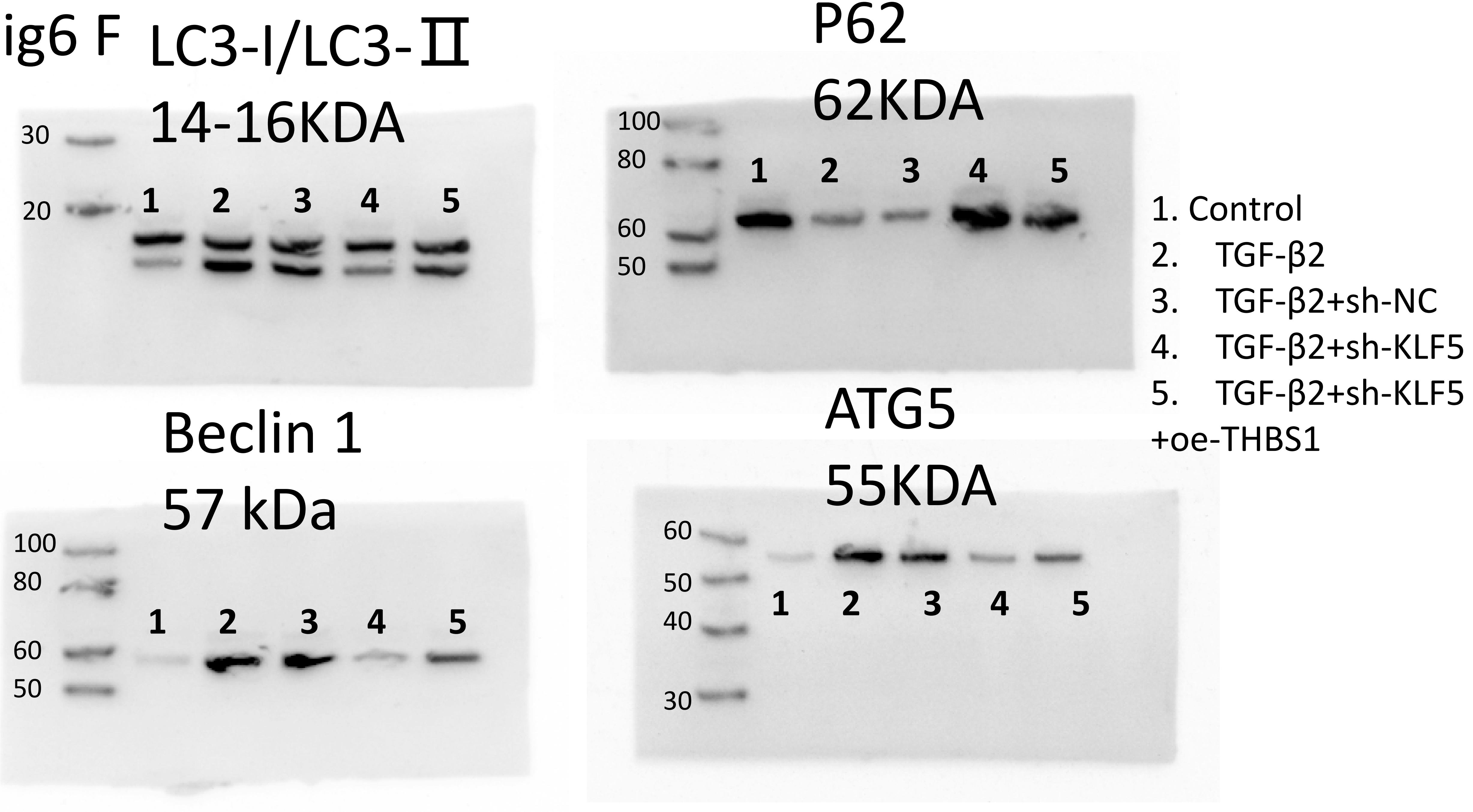
LC3-I/LC3-Ⅱ
14-16KDA
30
20
1 2 3 4 5
P62
62KDA
100
80
60
50
1 2 3 4 5
1. Control
2. TGF-β2
3. TGF-β2+sh-NC
4. TGF-β2+sh-KLF5
5. TGF-β2+sh-KLF5 +oe-THBS1
Beclin 1
57 kDa
100
80
60
50
1 2 3 4 5
ATG5
55KDA
60
50
40
30
1 2 3 4 5

Fig6 F

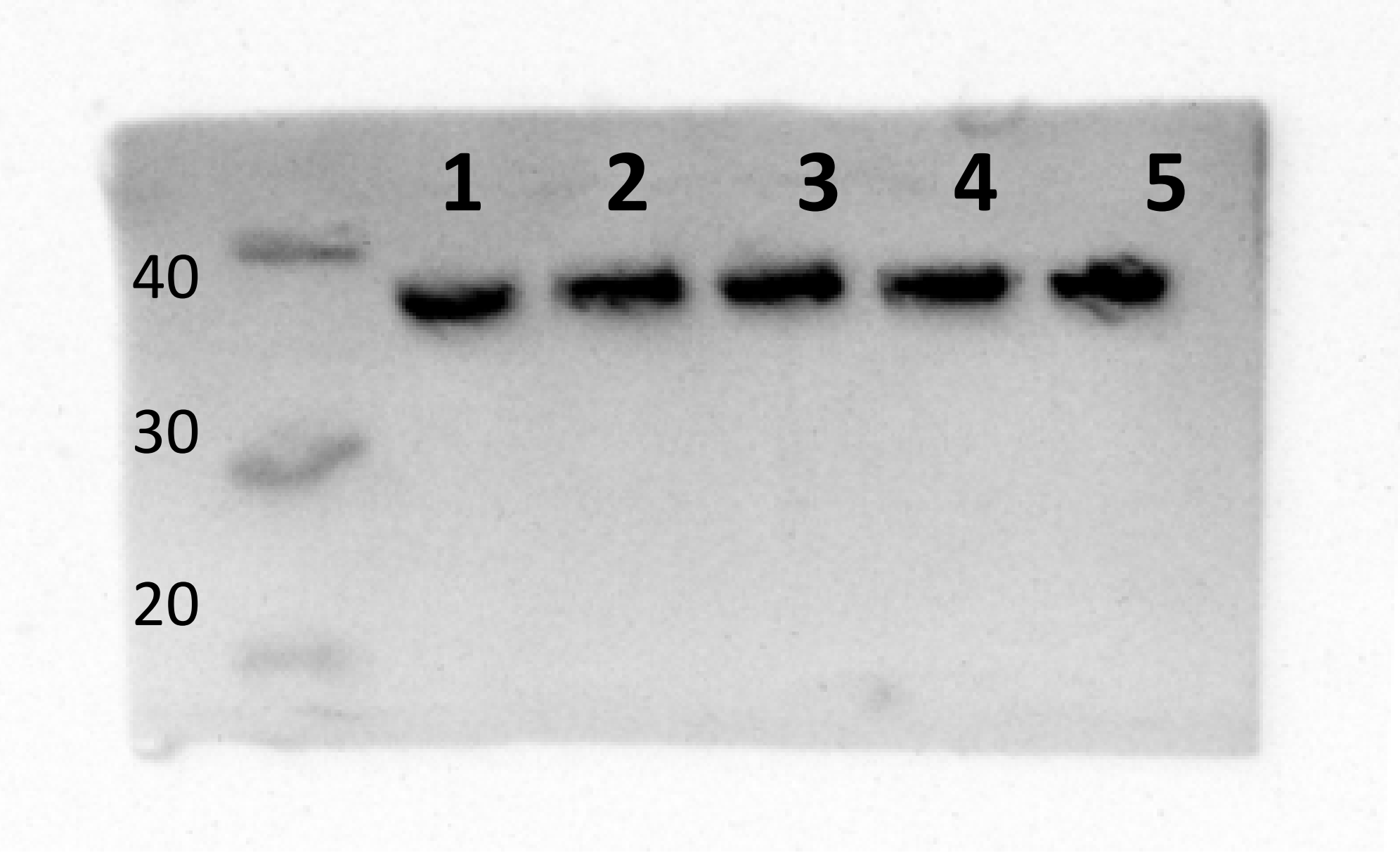

GAPDH
36KDA

1. Control
2. TGF-β2
3. TGF-β2+sh-NC
4. TGF-β2+sh-KLF5
5. TGF-β2+sh-KLF5
+oe-THBS1

Fig6 G

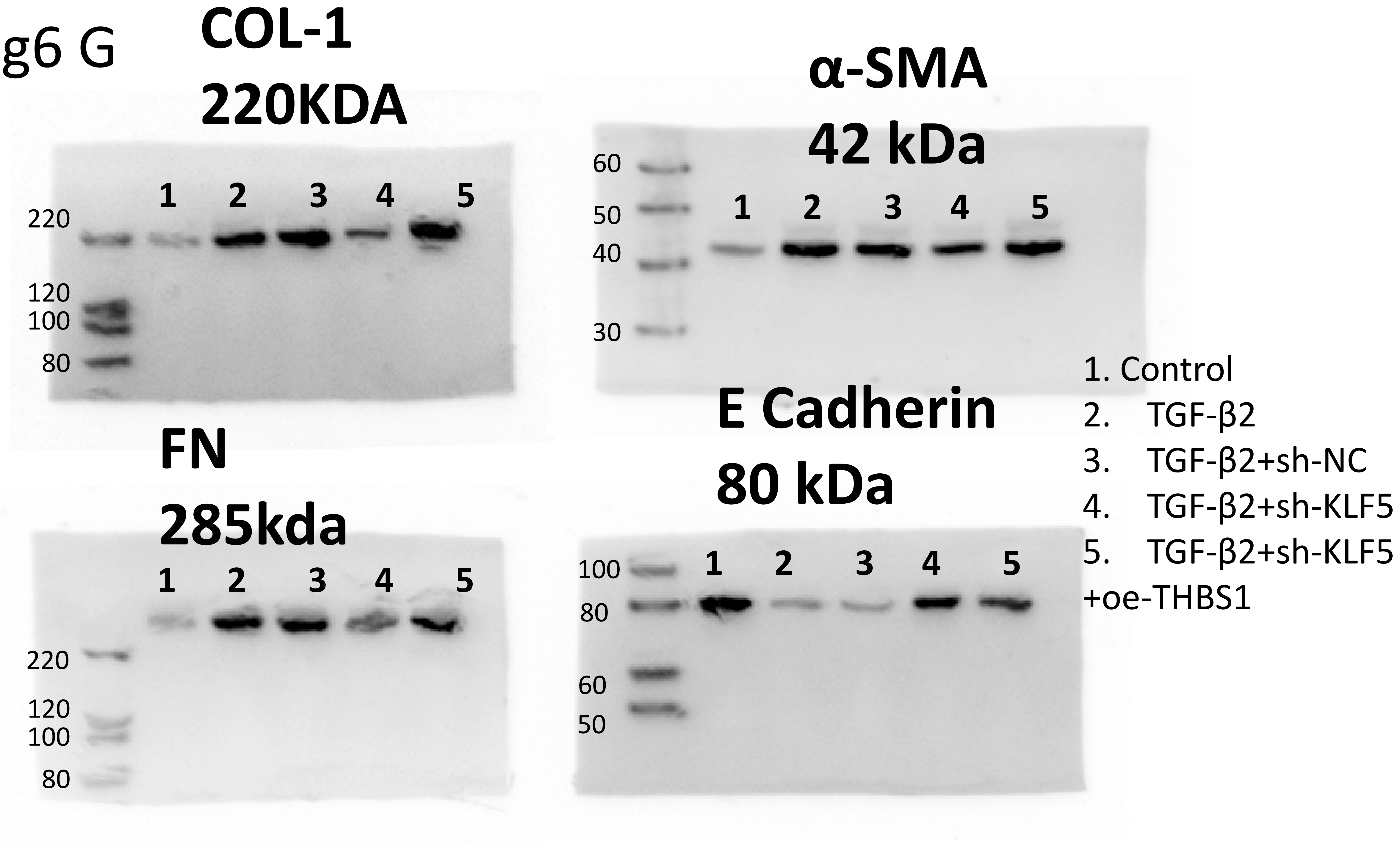

COL-1
220KDA
1 2 3 4 5
220
120
100
80
α-SMA
42 kDa
60
50
40
30
1 2 3 4 5
FN
285kda
1 2 3 4 5
220
120
100
80
E Cadherin
80 kDa
100
80
60
50
1 2 3 4 5
1. Control
2. TGF-β2
3. TGF-β2+sh-NC
4. TGF-β2+sh-KLF5
5. TGF-β2+sh-KLF5
+oe-THBS1

Fig6 G

GAPDH
36KDA

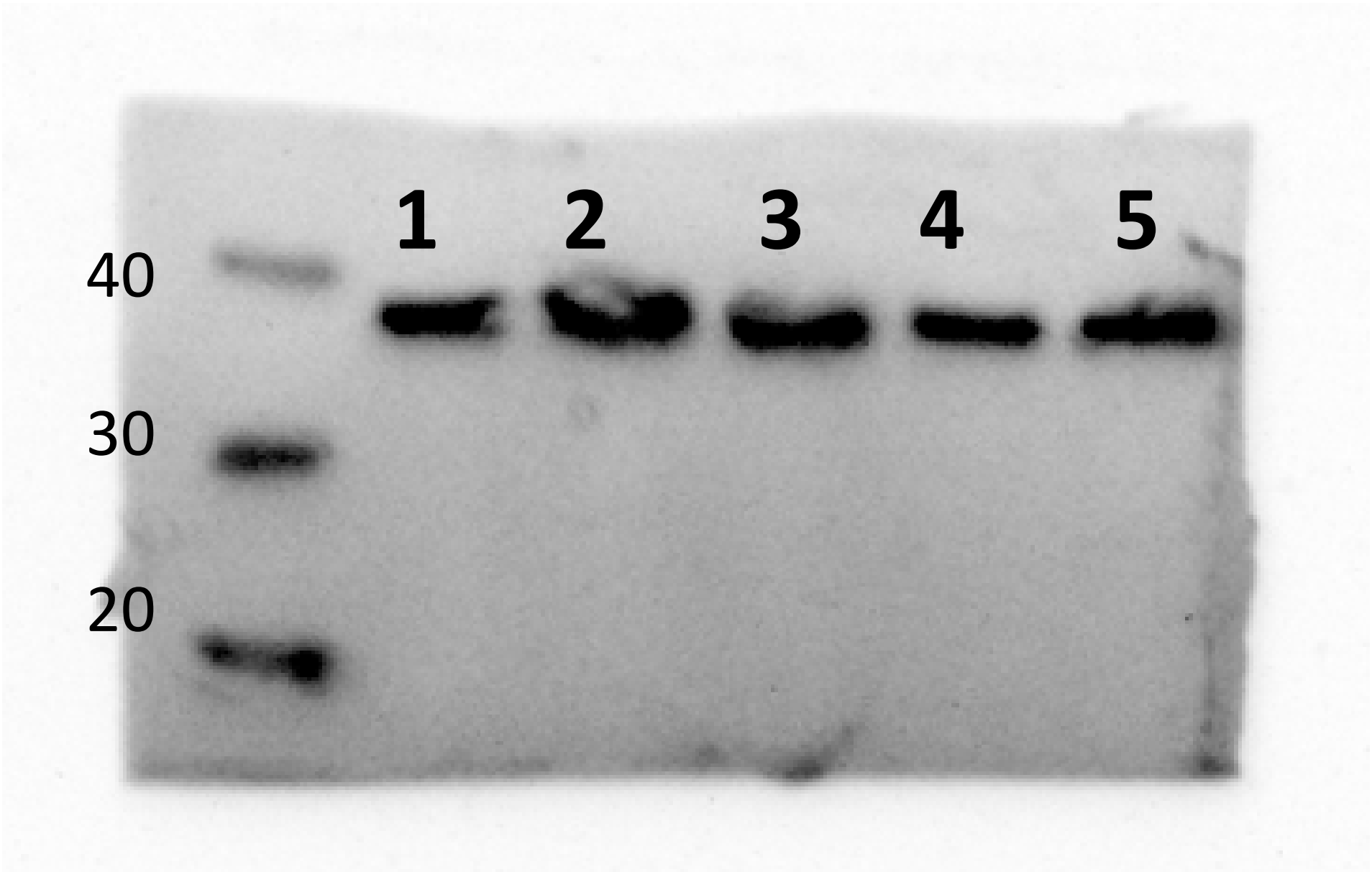

1. Control
2. TGF-β2
3. TGF-β2+sh-NC
4. TGF-β2+sh-KLF5
5. TGF-β2+sh-KLF5 +oe-THBS1

Fig2 E

THBS1
180KDA

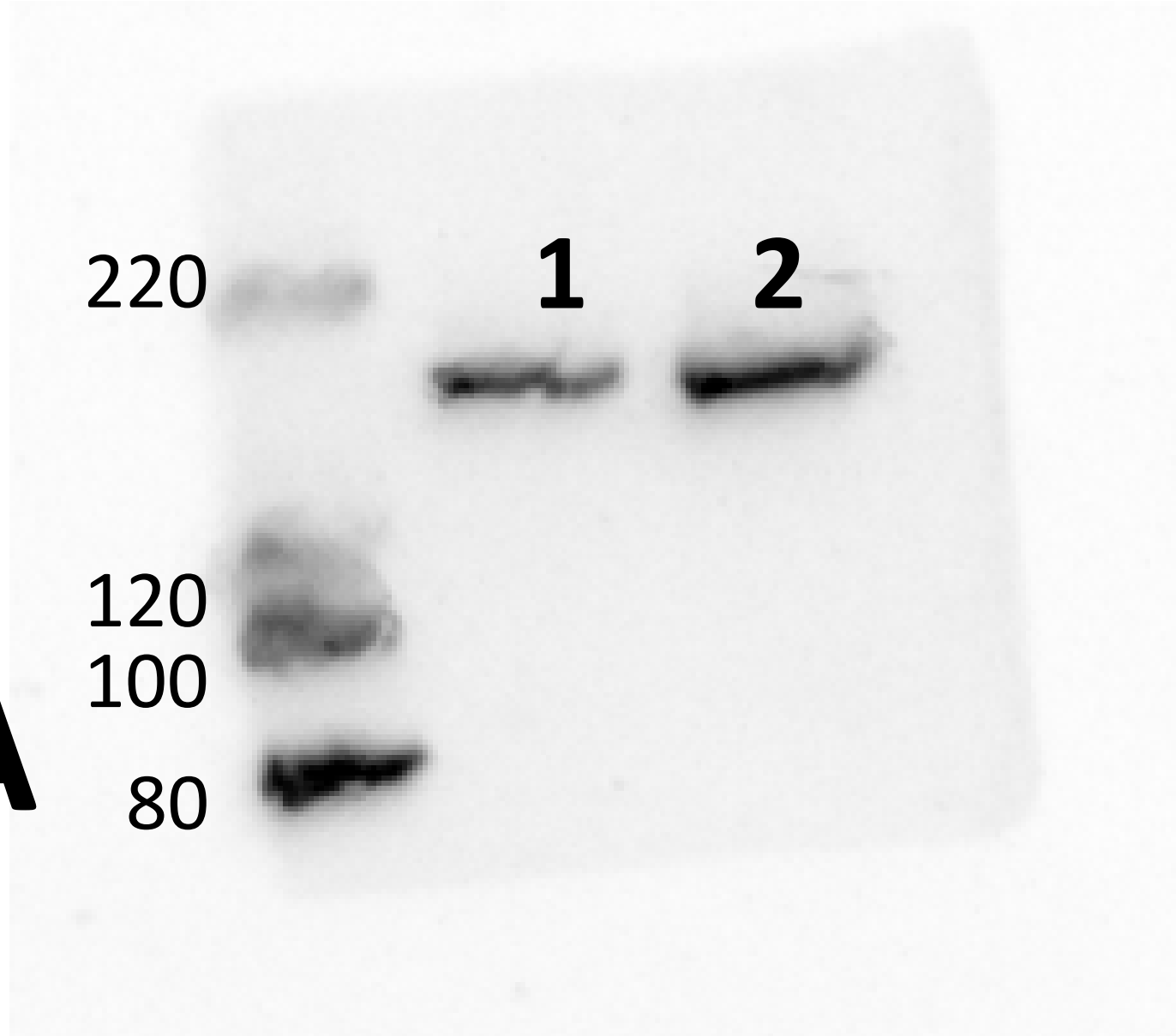

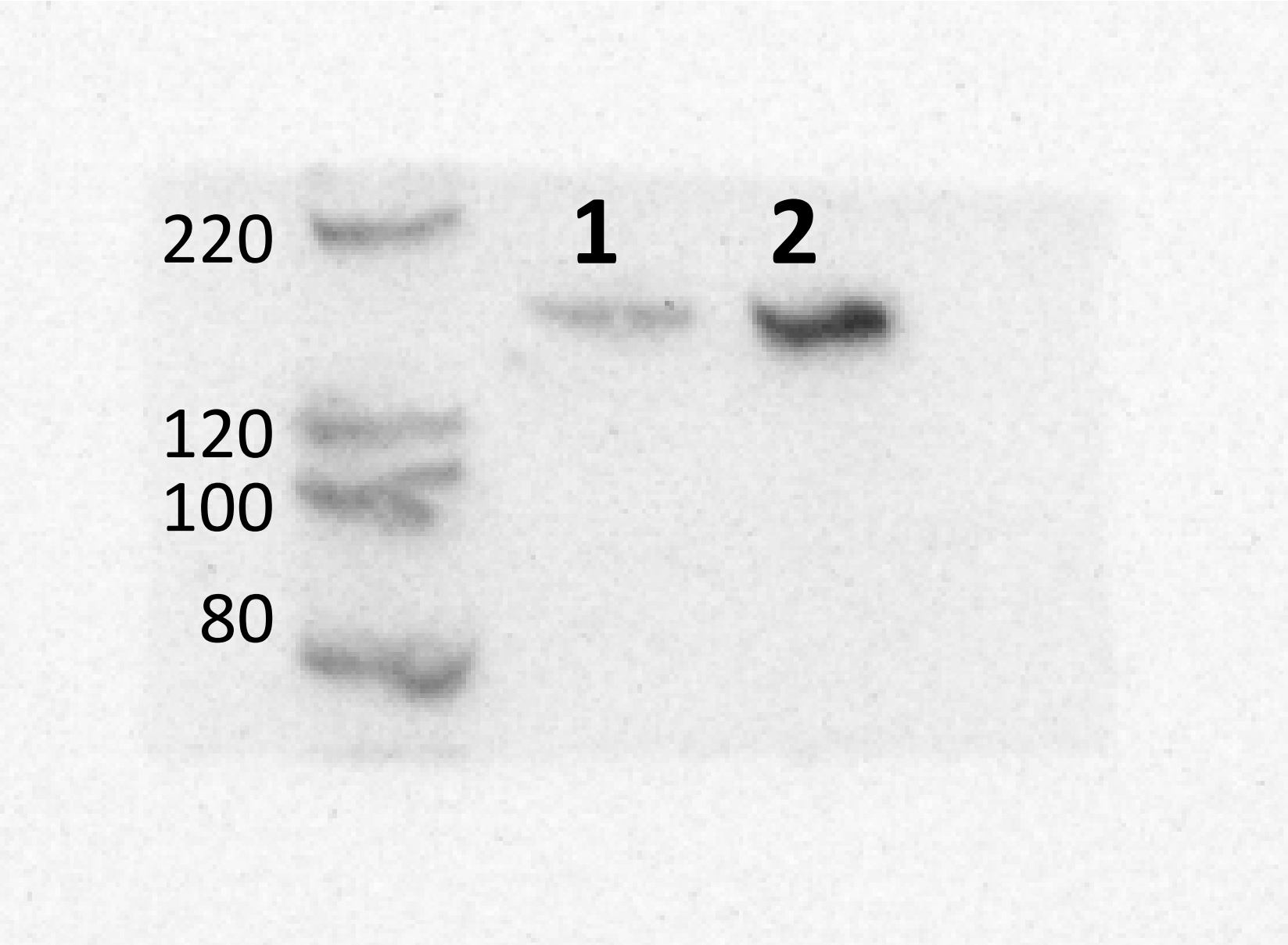

GAPDH
36KDA

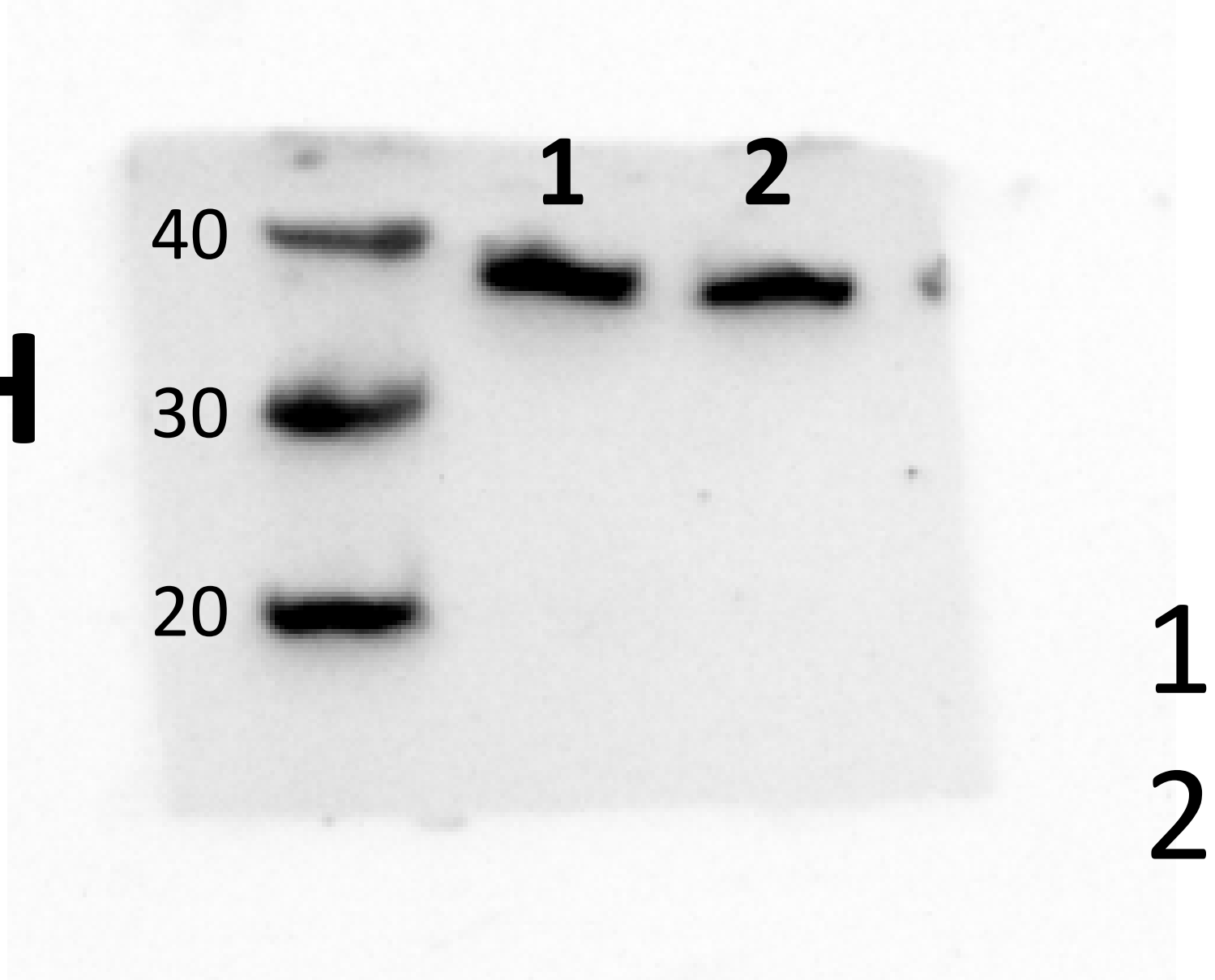

1. Control
2. TGF-β2

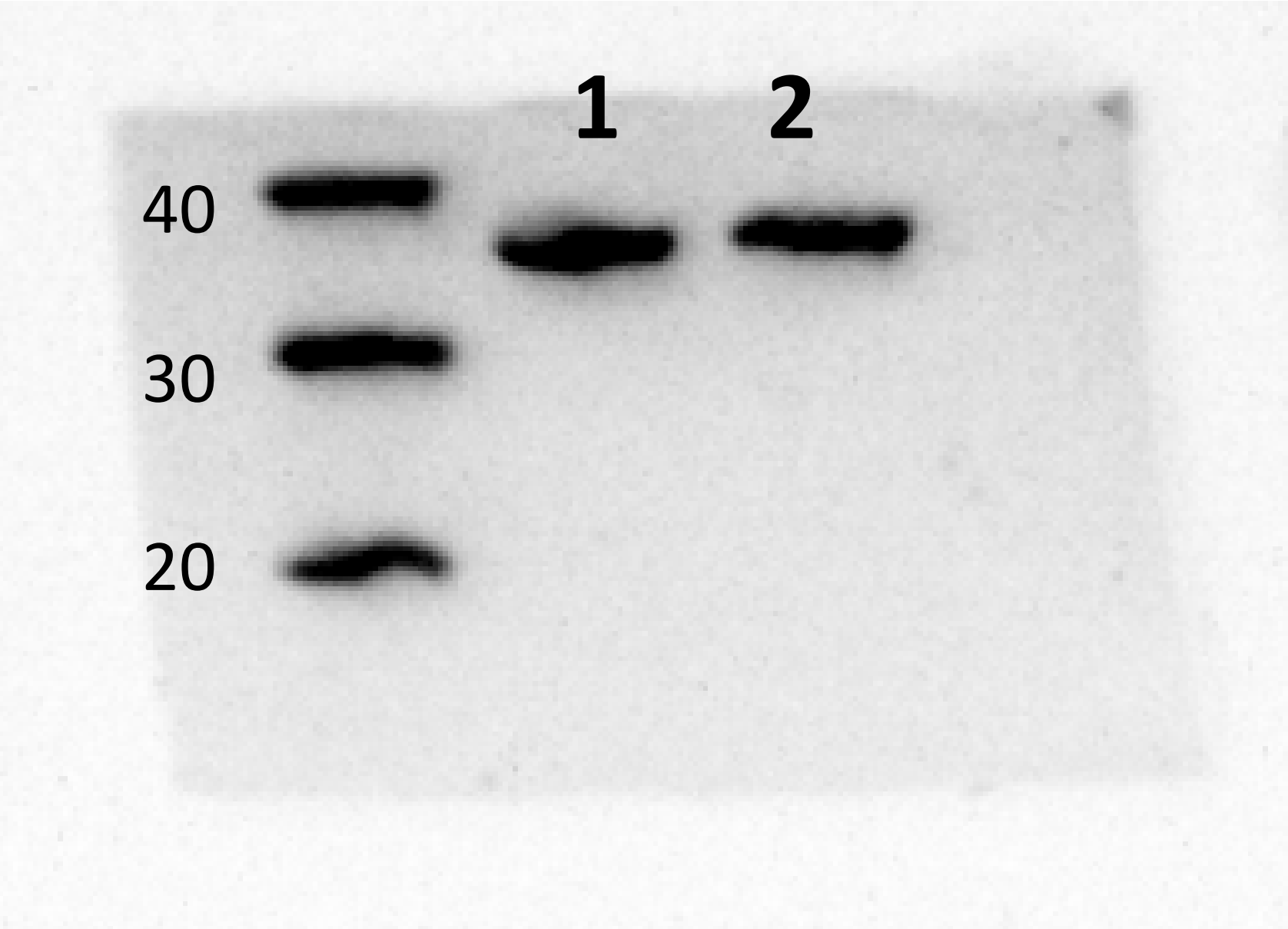

Fig2 F

1. Control
2. TGF-β2
3. TGF-β2+sh-NC
4. TGF-β2+sh-THBS1#1
5. TGF-β2+sh-THBS1#2
6. TGF-β2+sh-THBS1#3

THBS1
180KDA

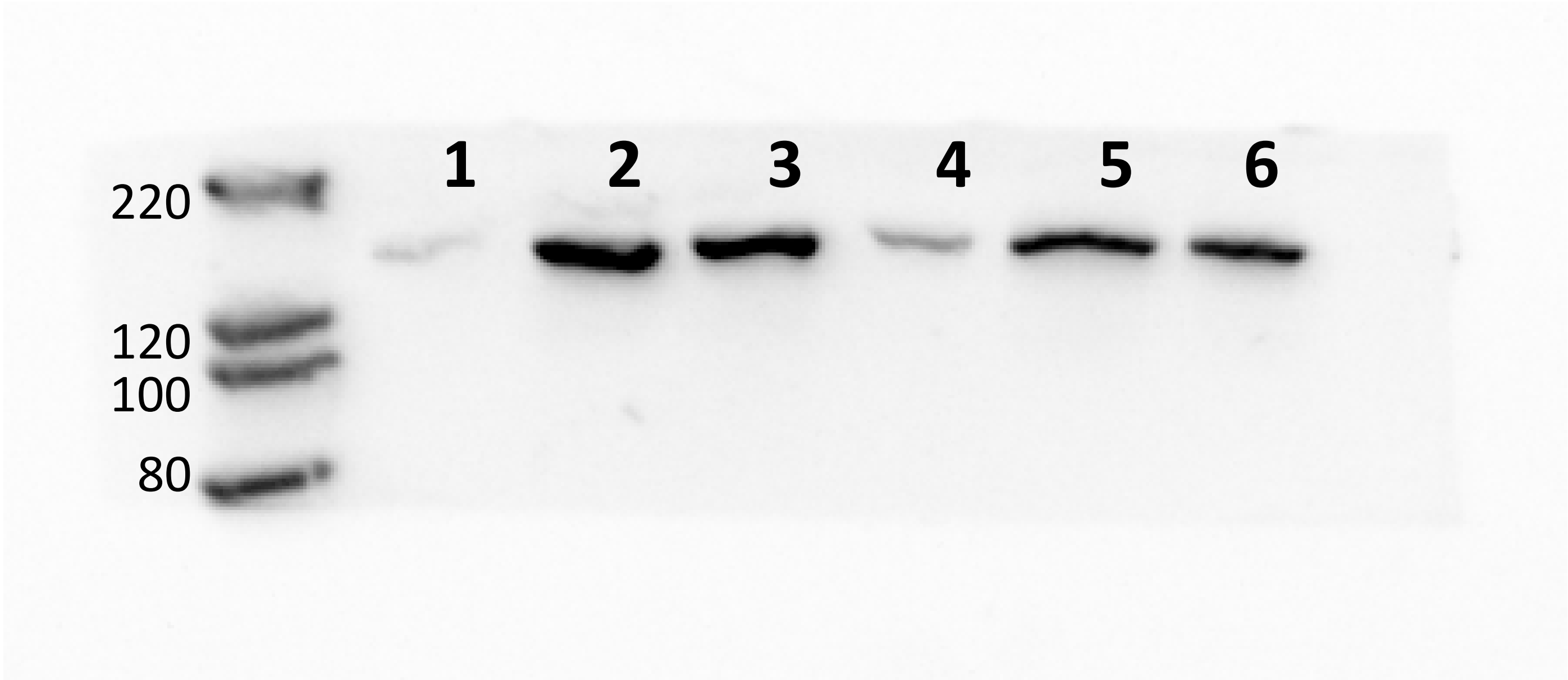

GAPDH
36KDA

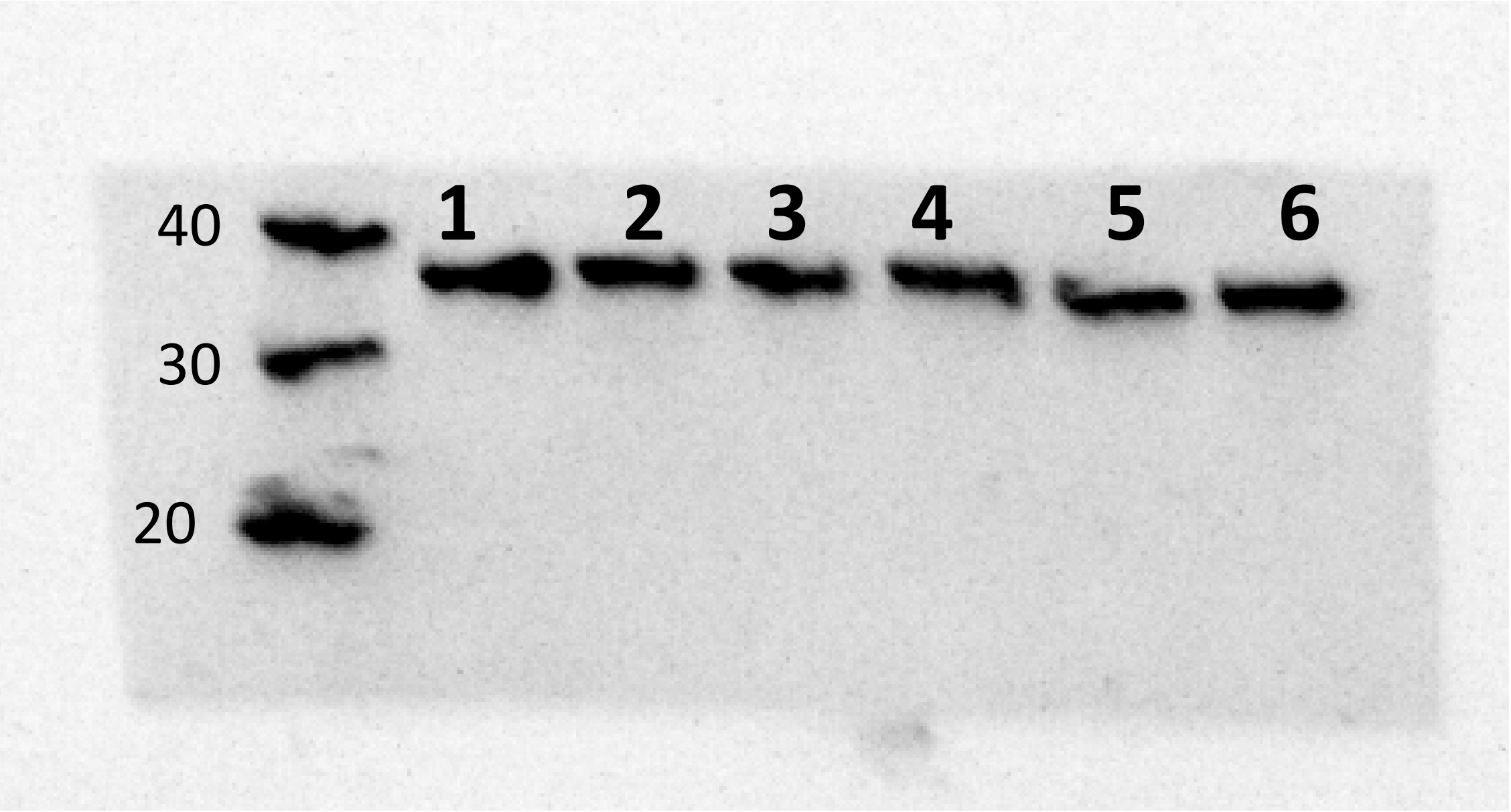

Fig2 F

1. Control
2. TGF-β2
3. TGF-β2+sh-NC
4. TGF-β2+sh-THBS1#1
5. TGF-β2+sh-THBS1#2
6. TGF-β2+sh-THBS1#3

THBS1
180KDA

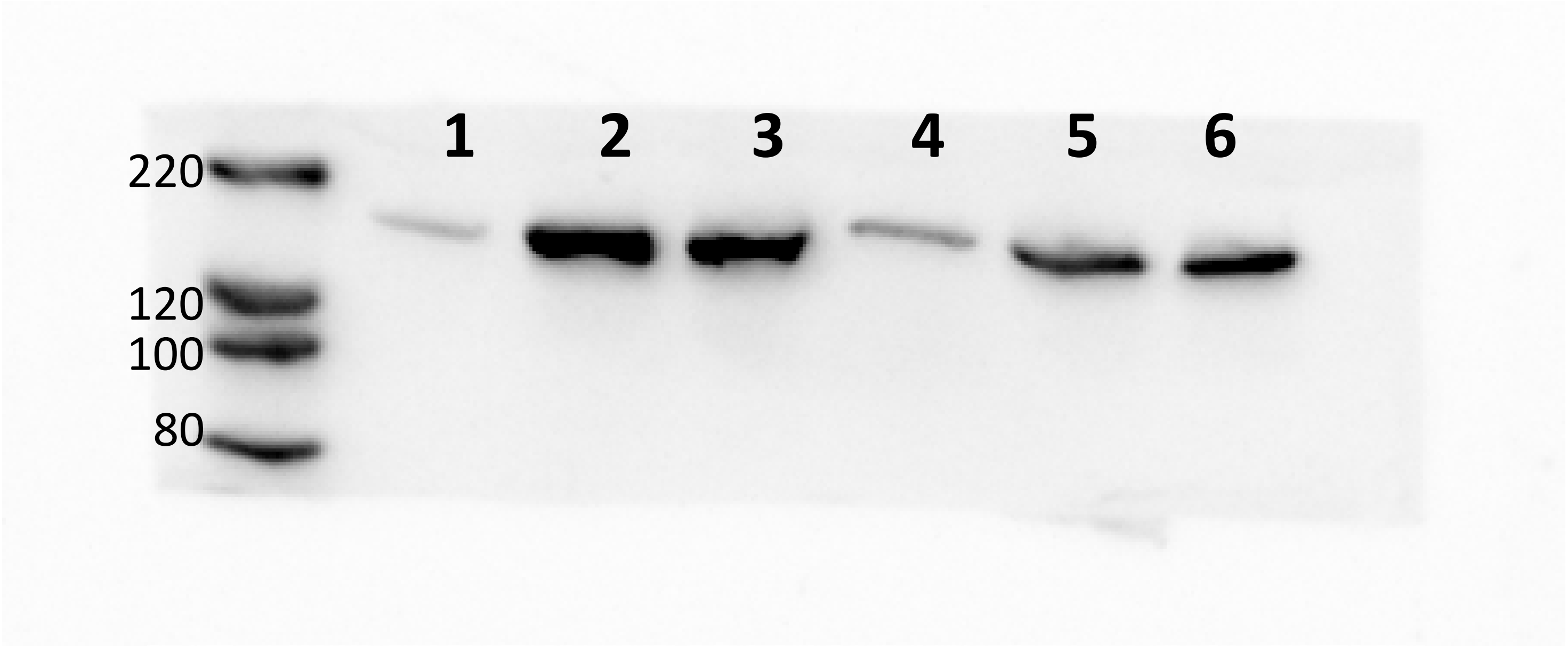

GAPDH
36KDA

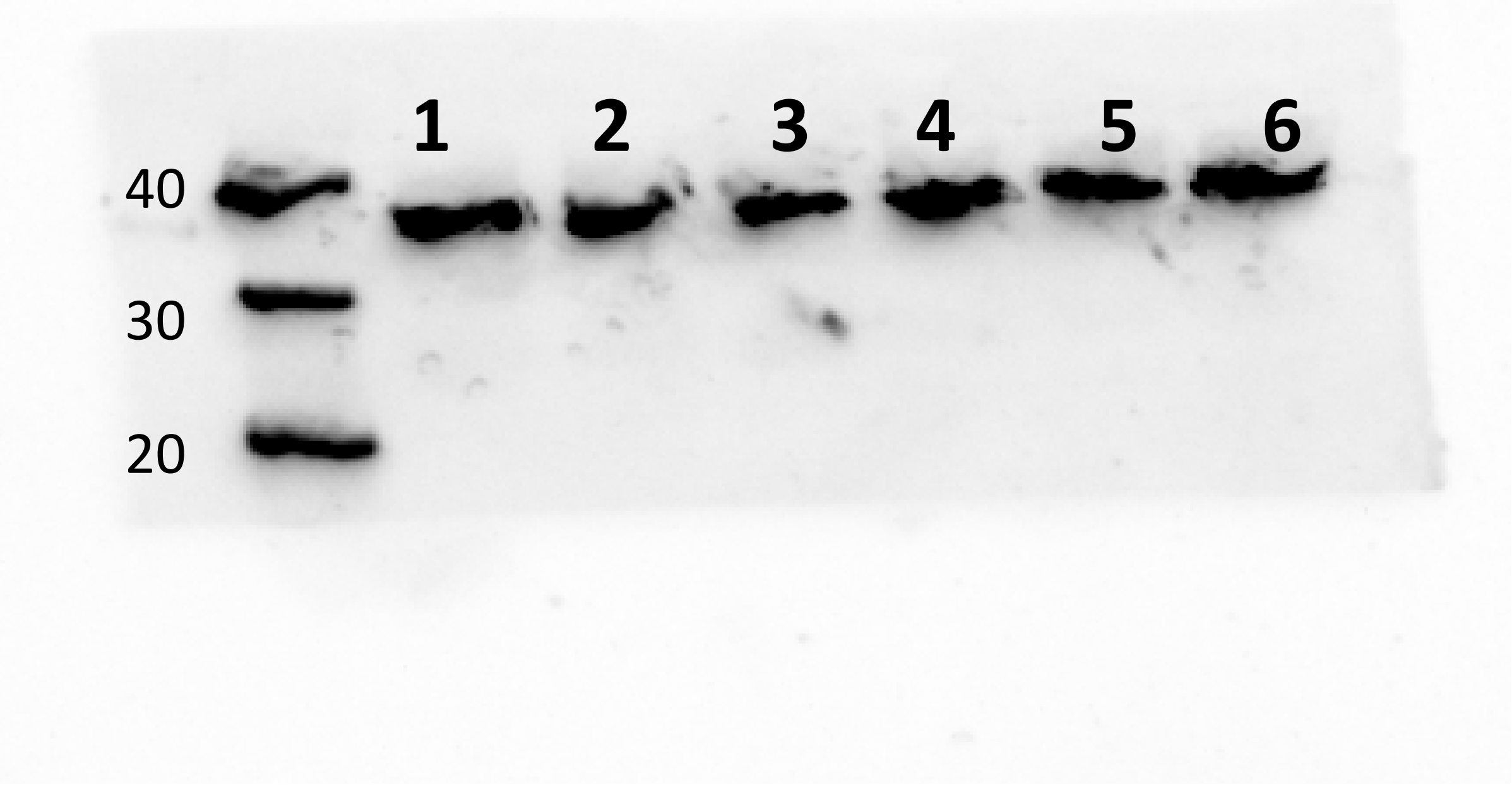

Fig4 A

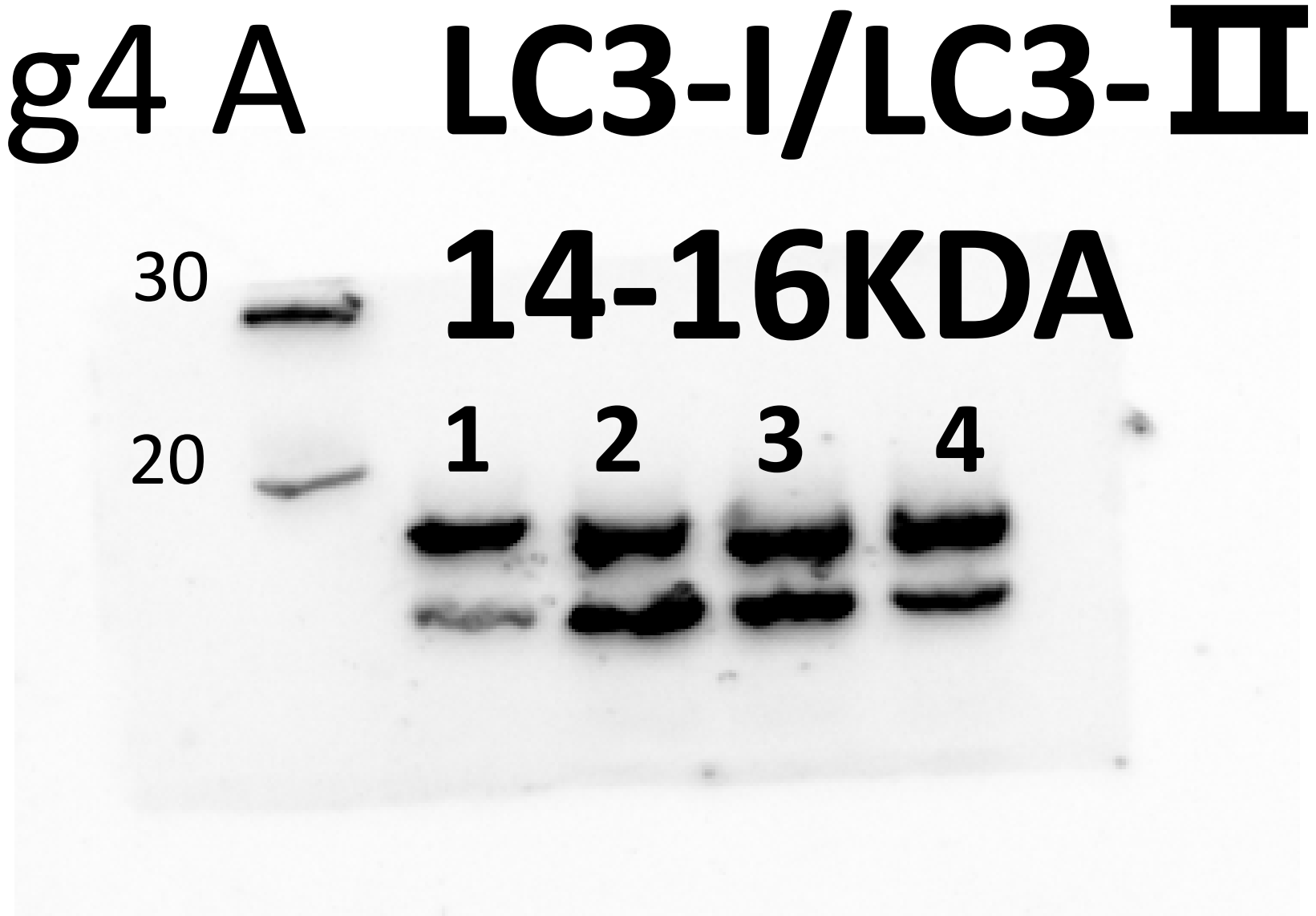

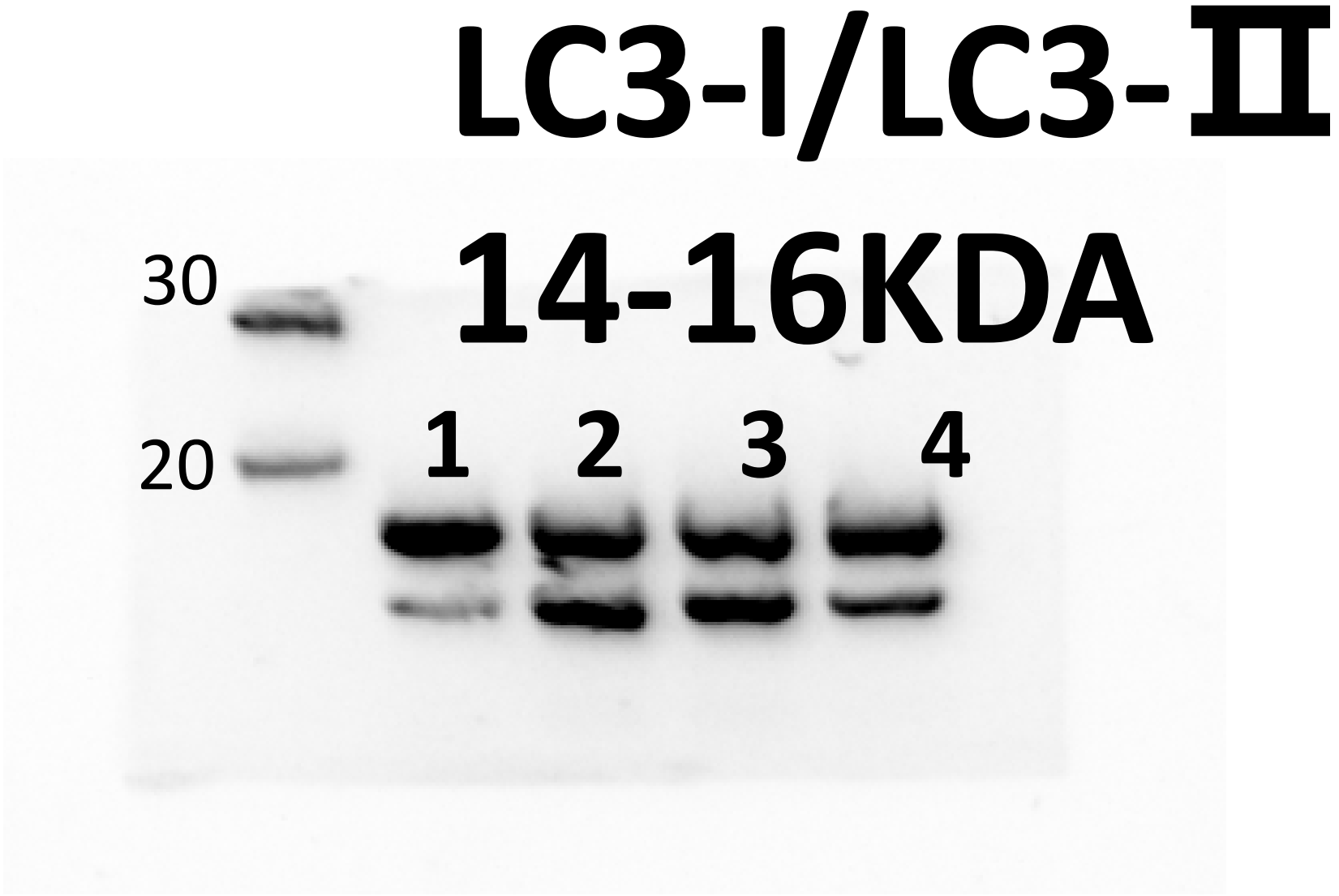

1. Control
2. TGF-β2
3. TGF-β2+sh-NC
4. TGF-β2+sh-THBS1

Fig4 A

Beclin 1
57 kDa

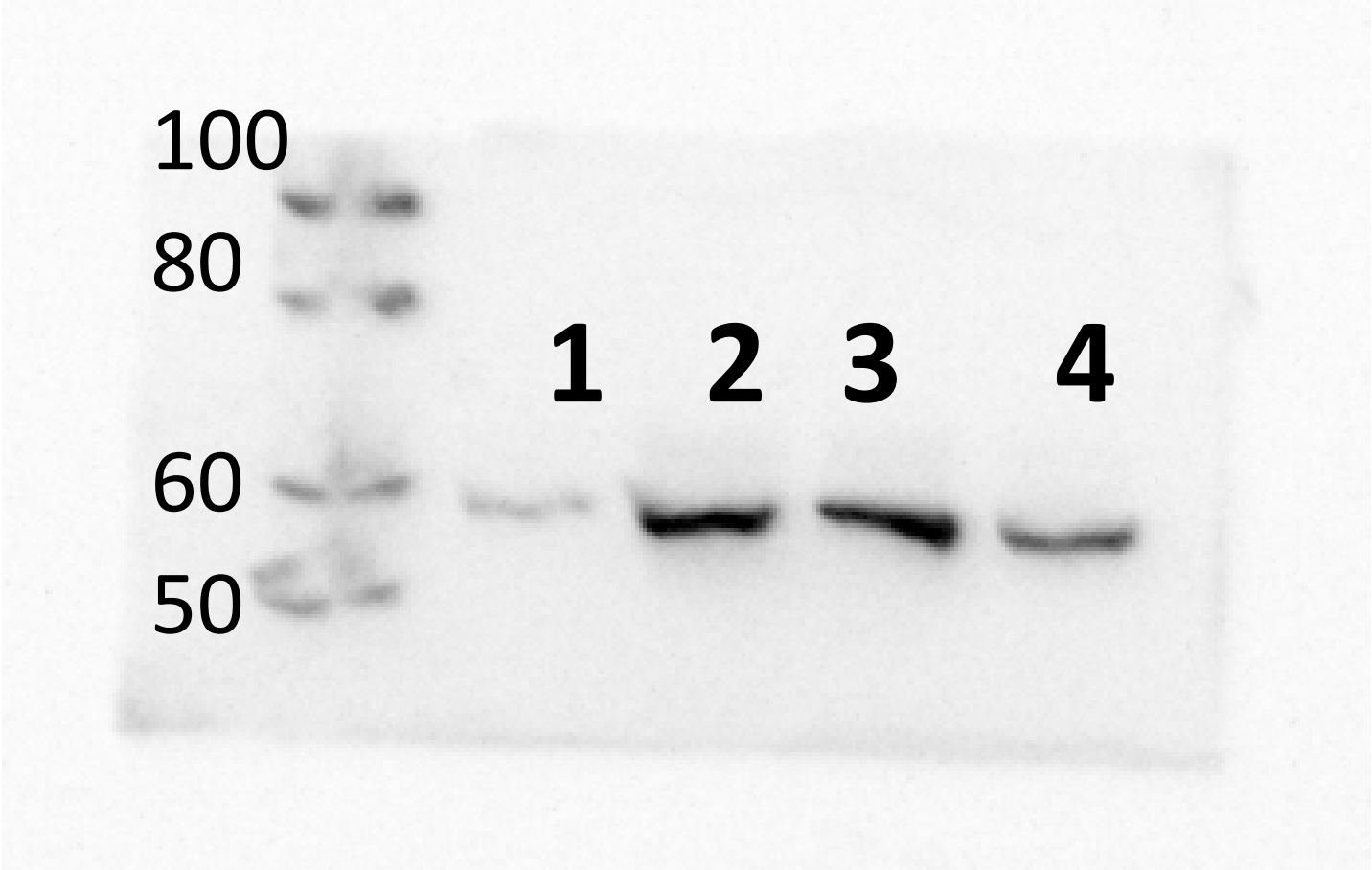

Beclin 1
57 kDa

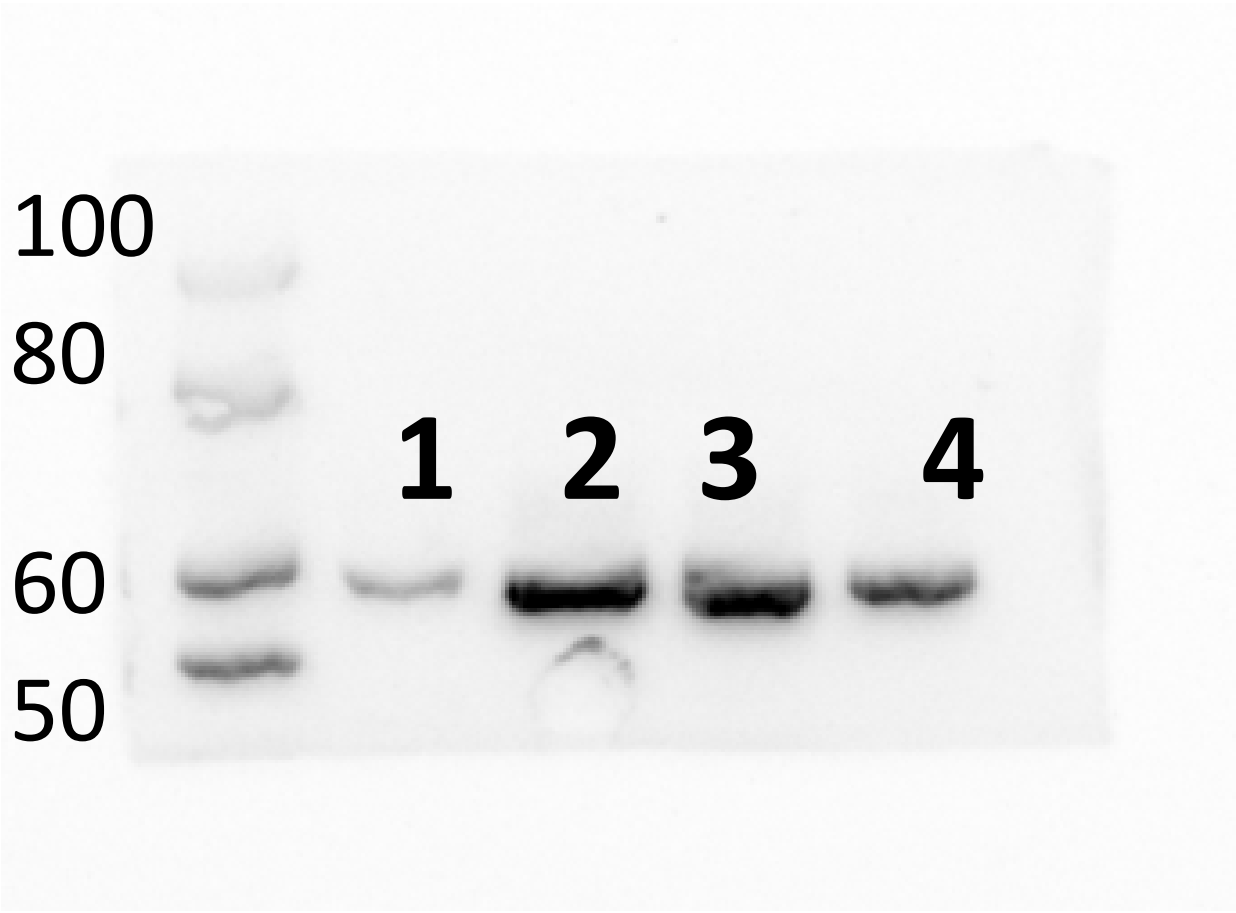

1. Control
2. TGF-β2
3. TGF-β2+sh-NC
4. TGF-β2+sh-THBS1

Fig4 A

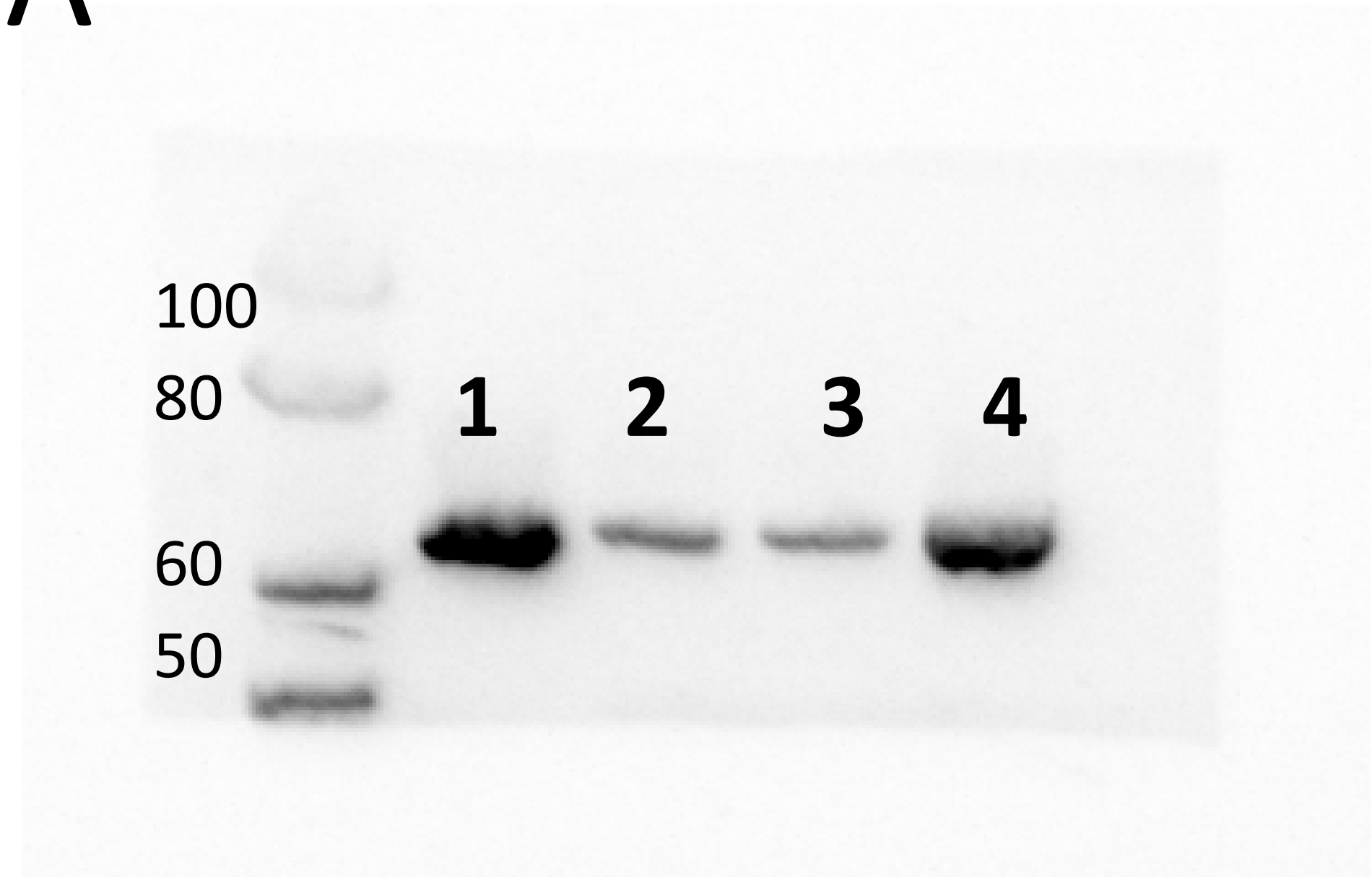

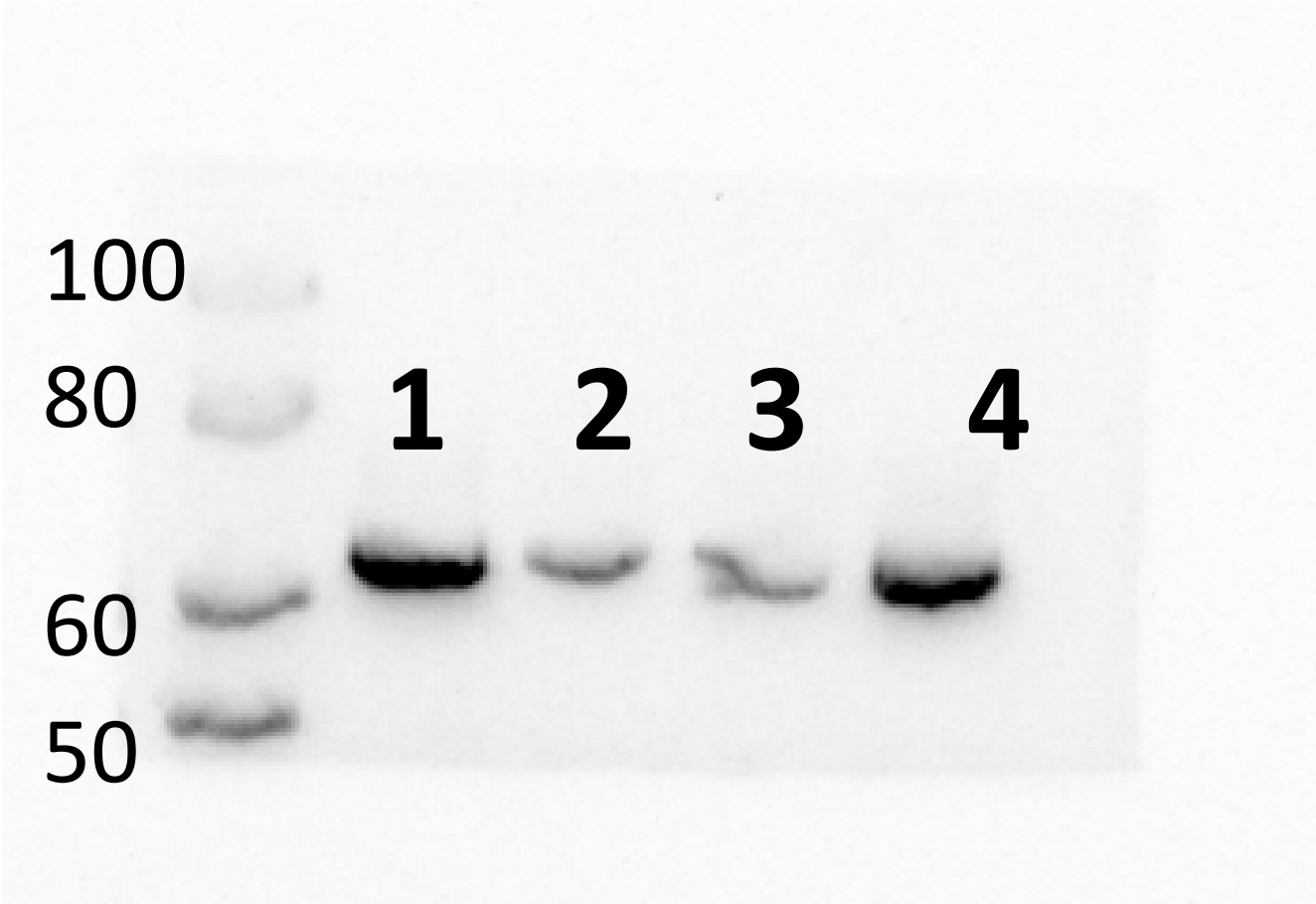

P62
62KDA

1. Control
2. TGF-β2
3. TGF-β2+sh-NC
4. TGF-β2+sh-THBS1

Fig4 A

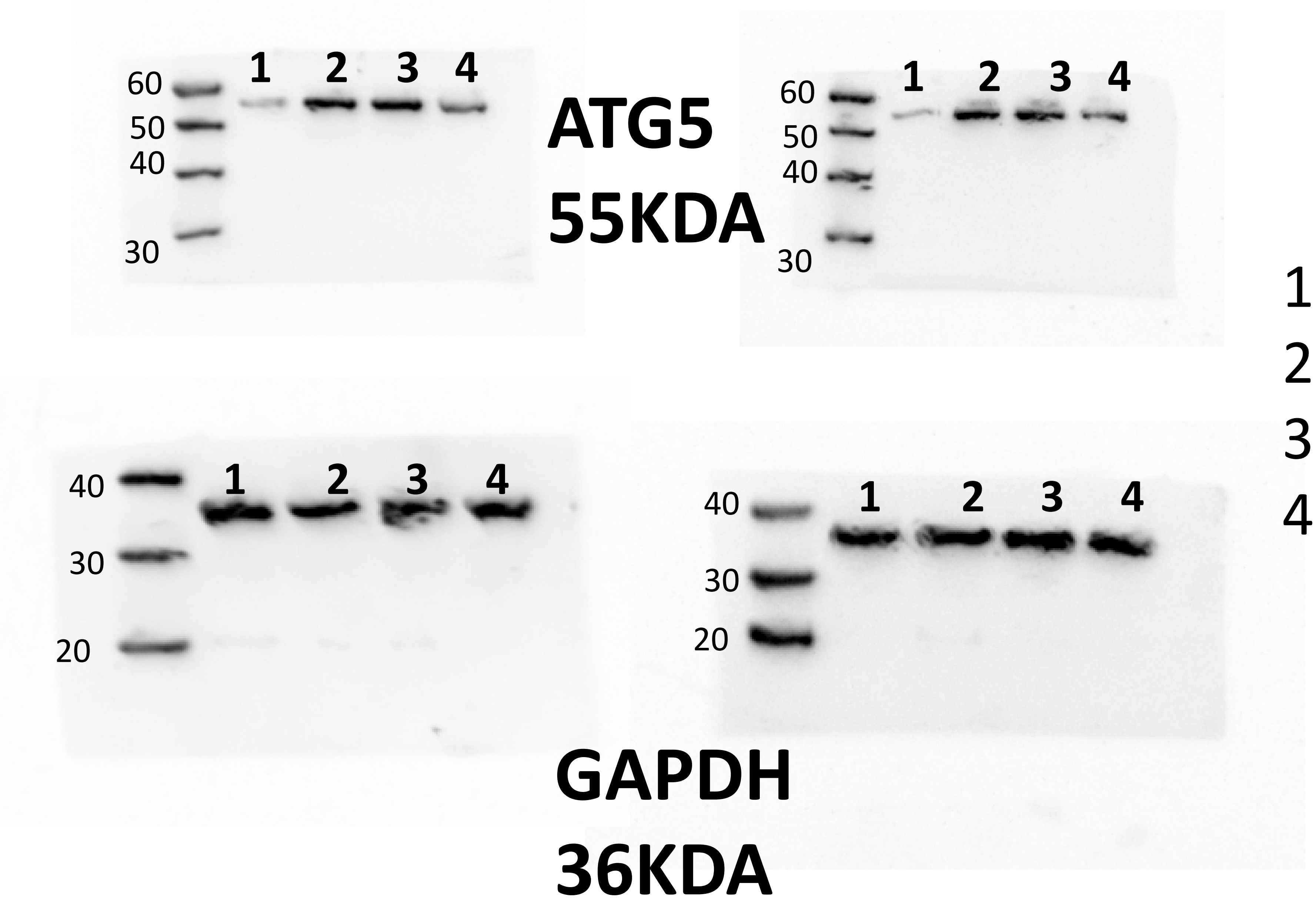

1. Control
2. TGF-β2
3. TGF-β2+sh-NC
4. TGF-β2+sh-THBS1

Fig4 B

COL-1

220KDA

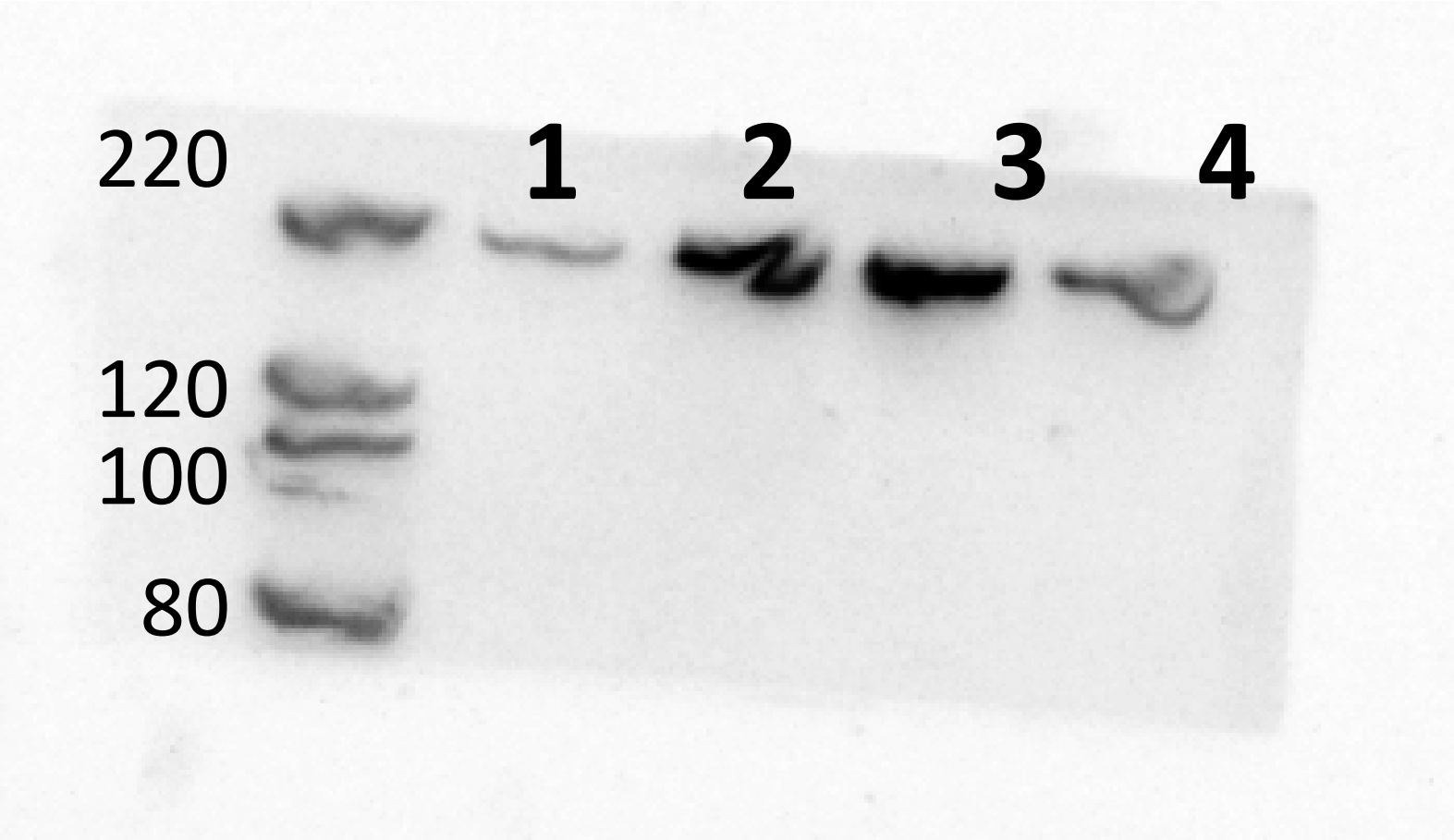

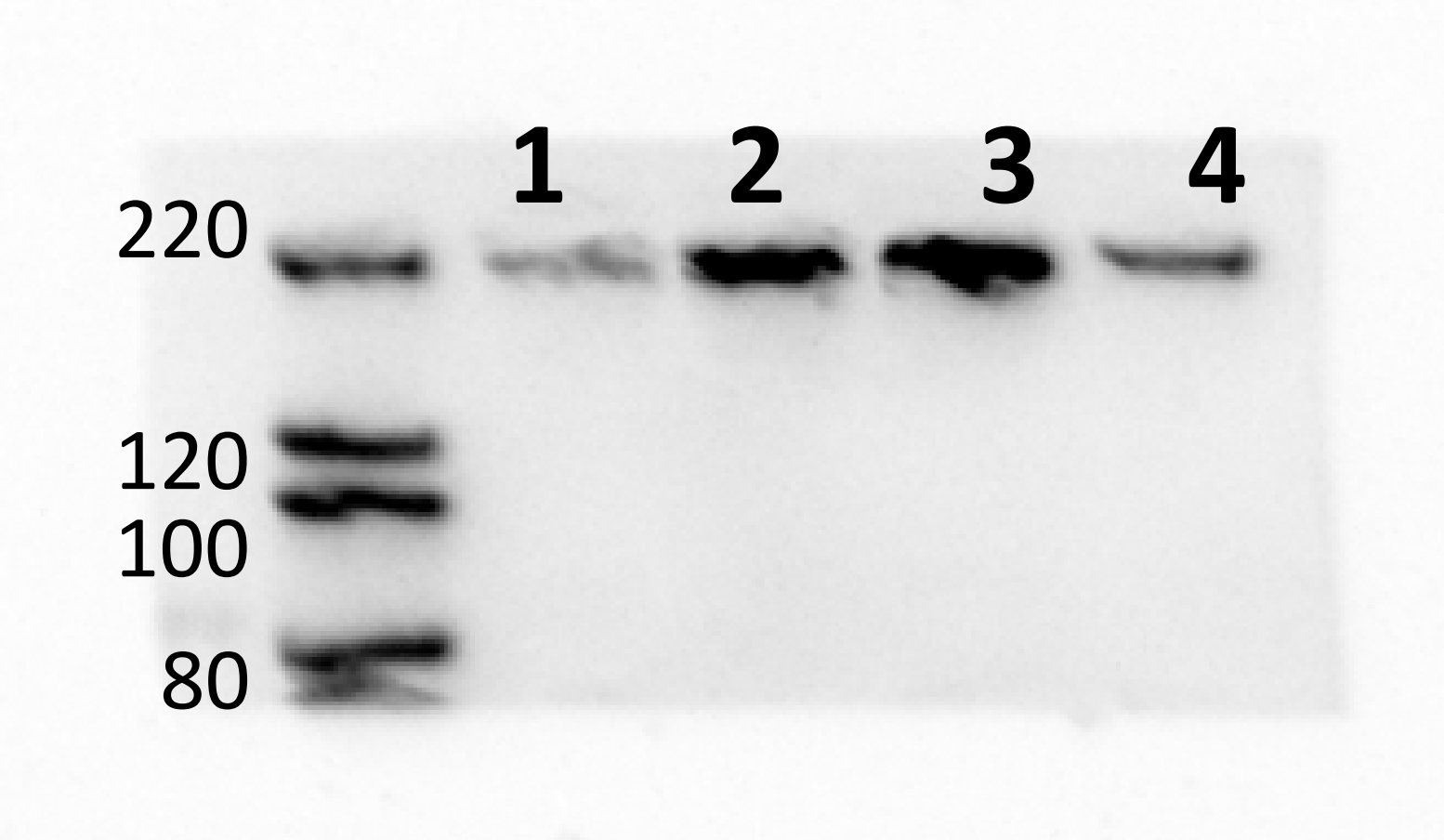

1. Control
2. TGF-β2
3. TGF-β2+sh-NC
4. TGF-β2+sh-THBS1

Fig4 B

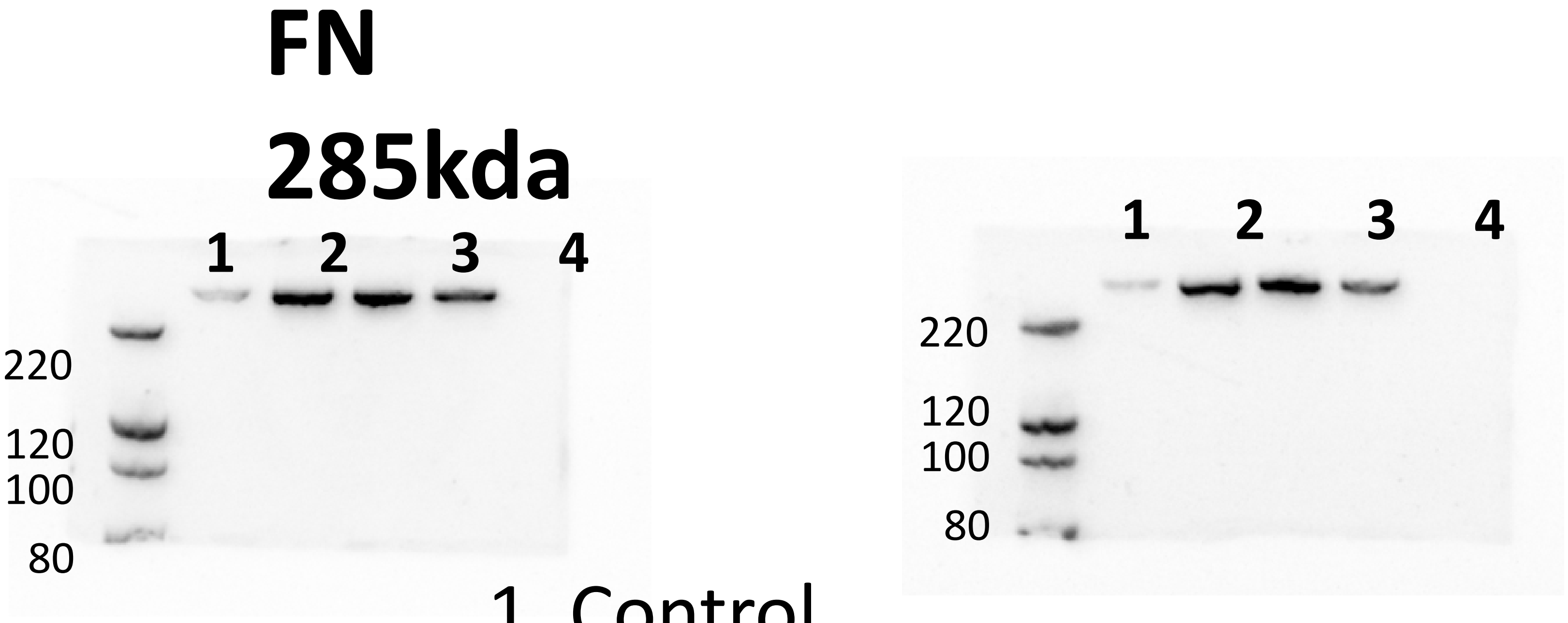

1. Control
2. TGF-β2
3. TGF-β2+sh-NC
4. TGF-β2+sh-THBS1

Fig4 B

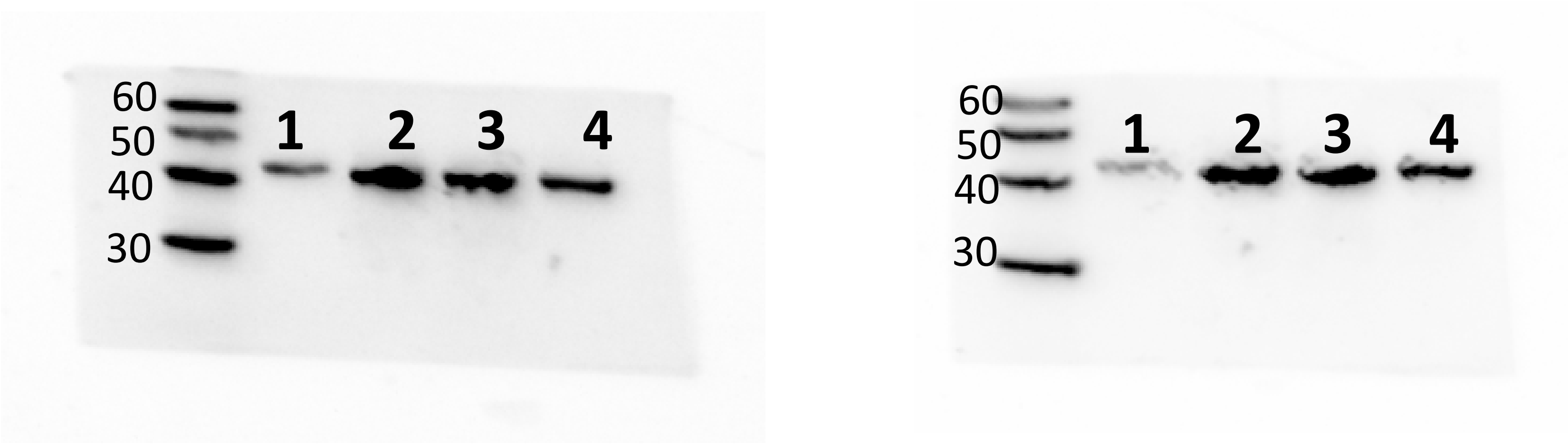

α-SMA

42 kDa

1. Control
2. TGF-β2
3. TGF-β2+sh-NC
4. TGF-β2+sh-THBS1

Fig4 B

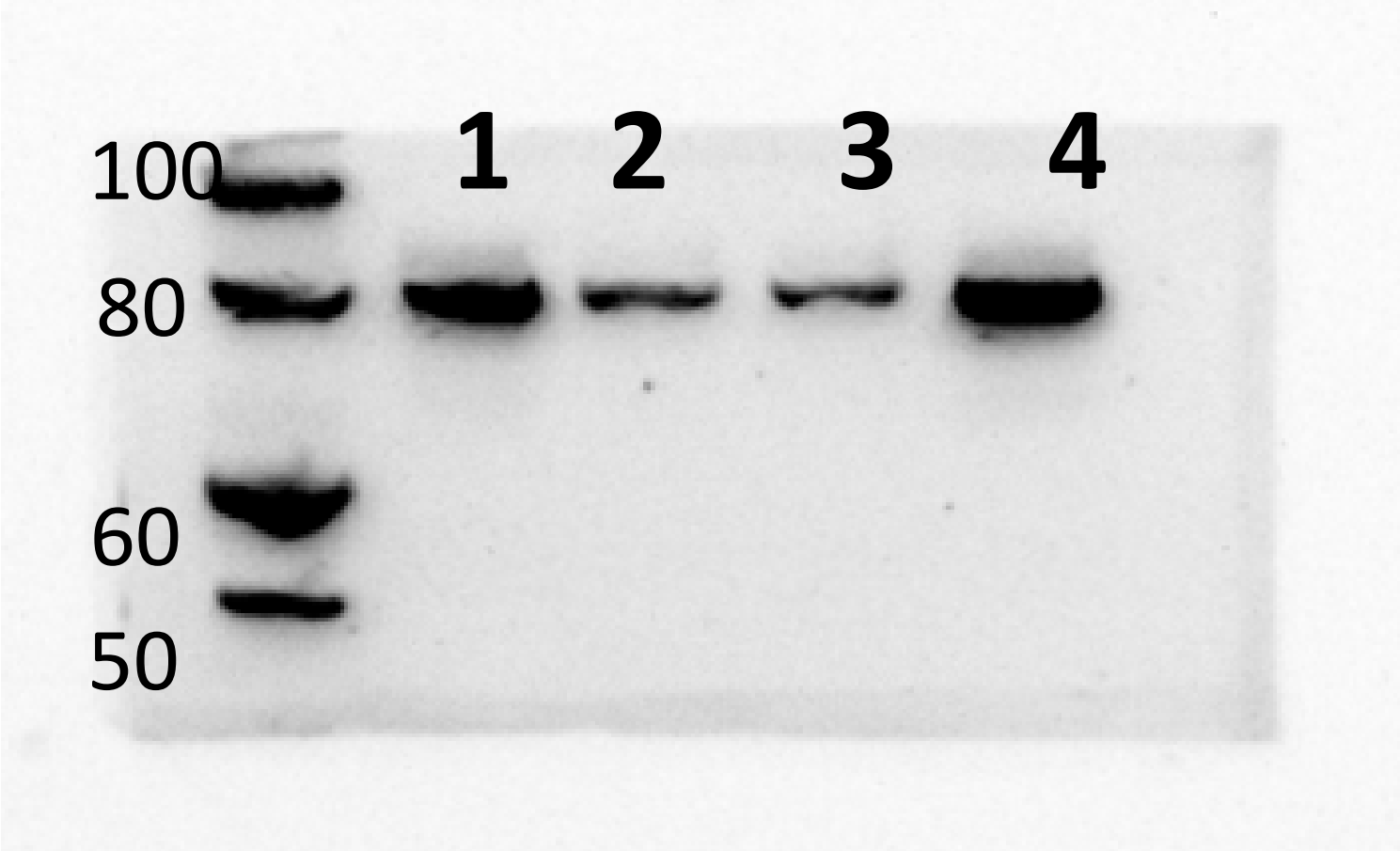

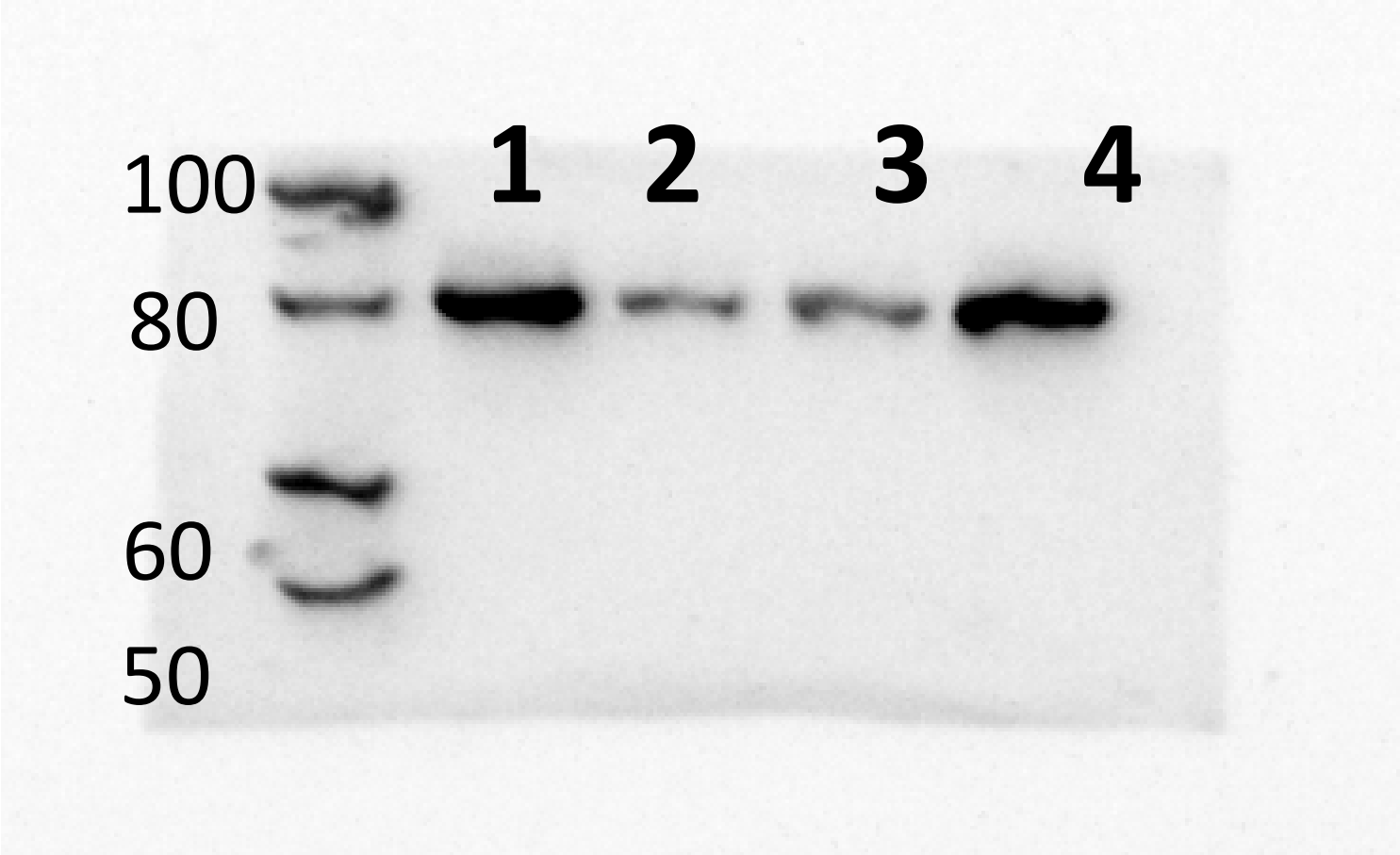

E Cadherin
80 kDa

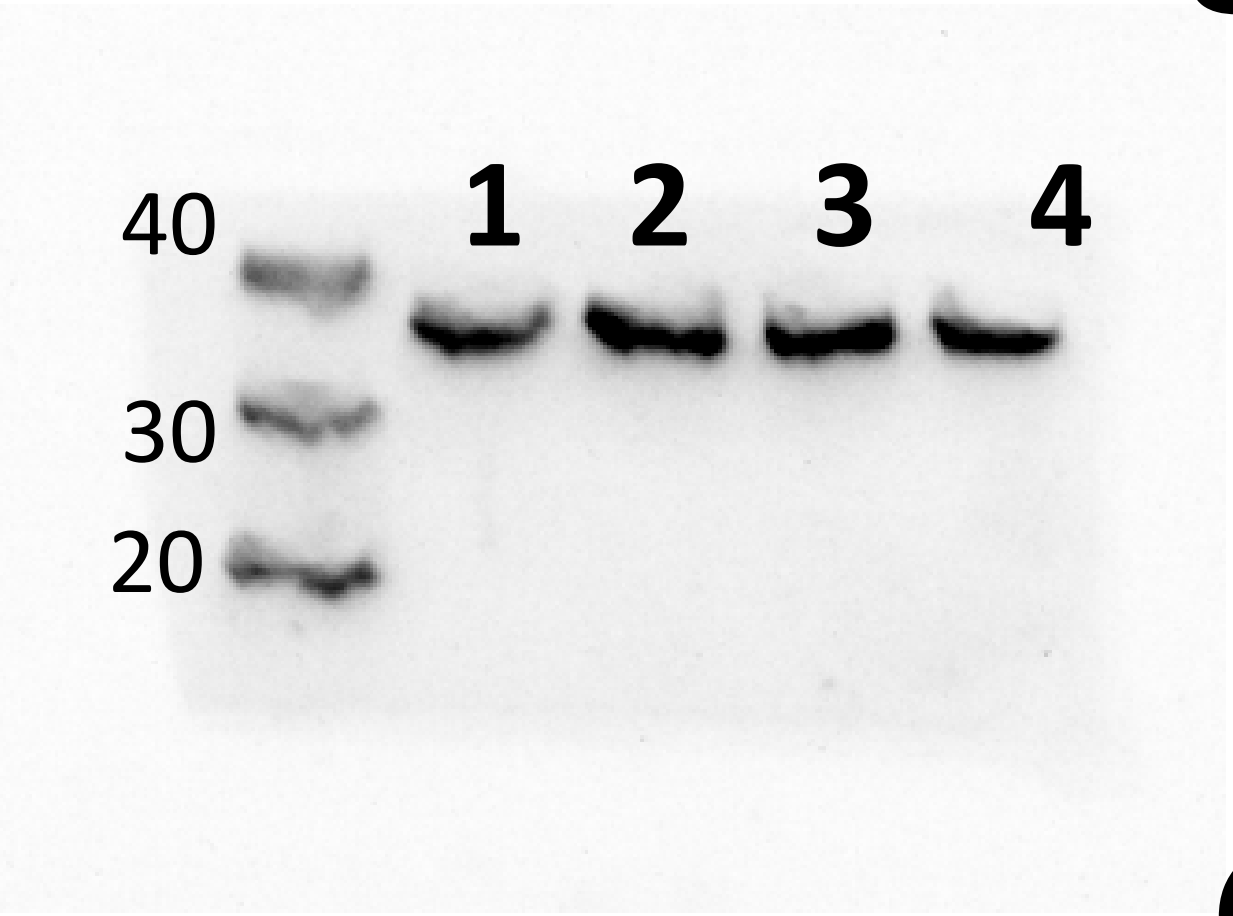

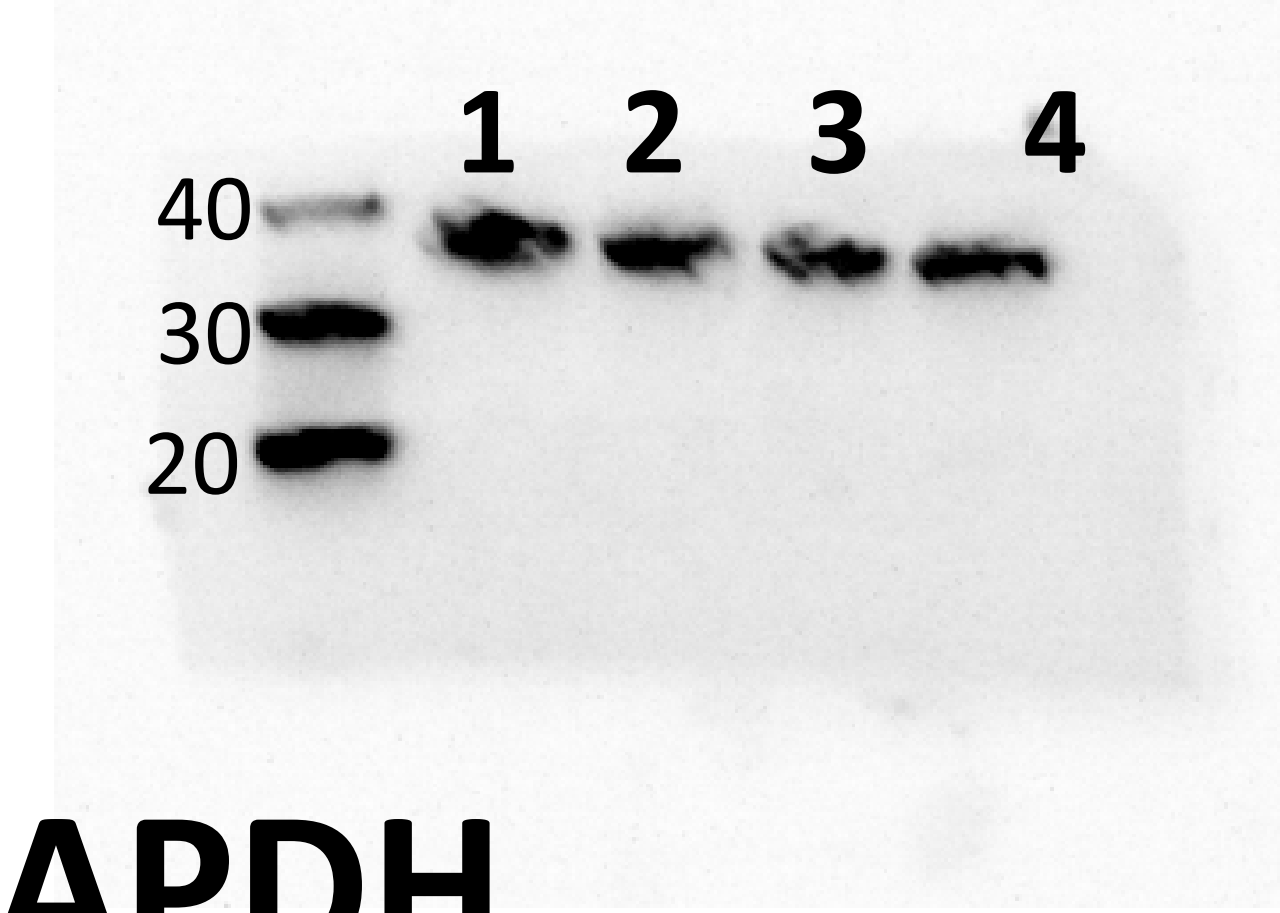

GAPDH
36KDA

1. Control
2. TGF-β2
3. TGF-β2+sh-NC
4. TGF-β2+sh-THBS1

Fig5 A

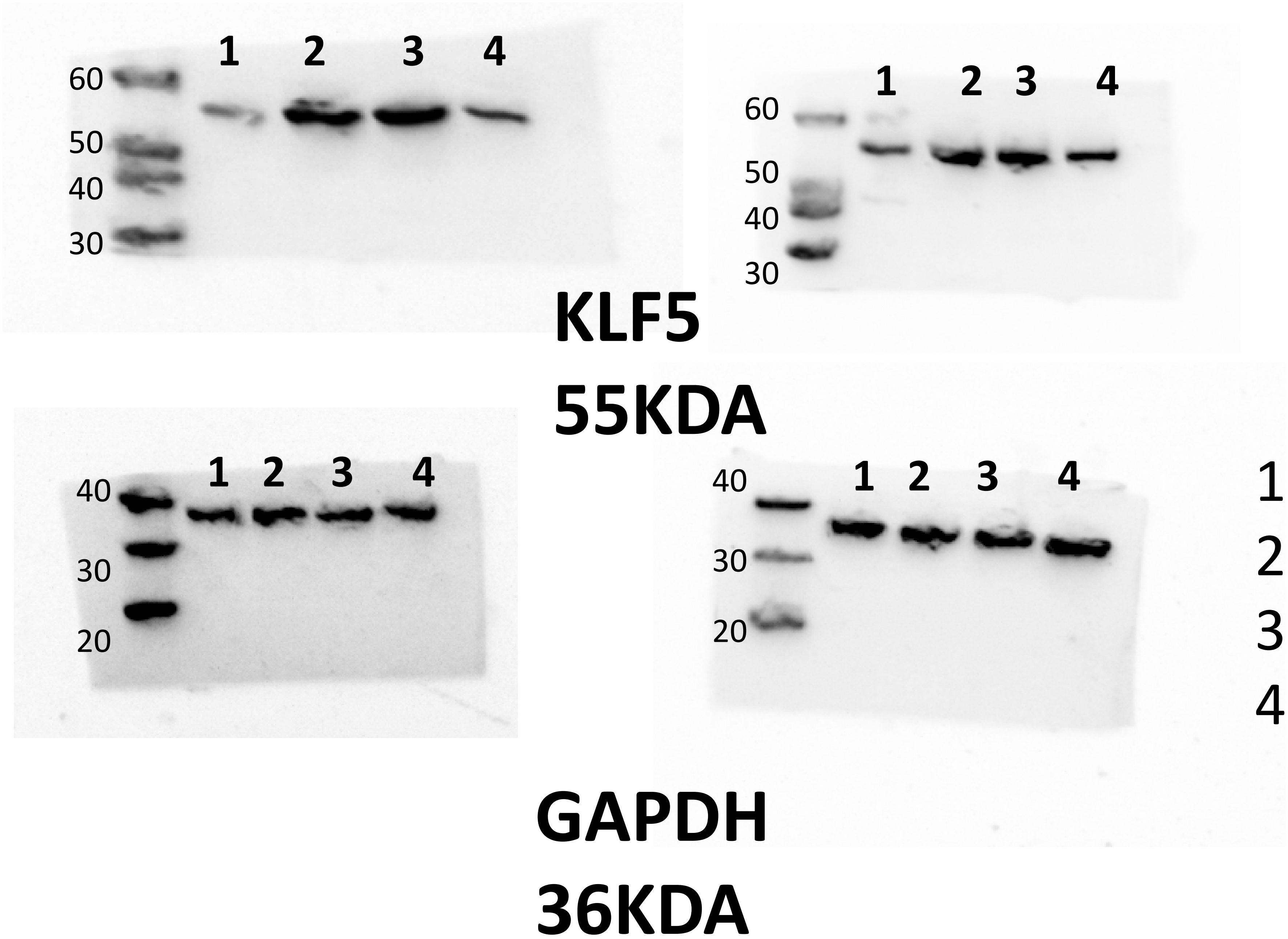

1. Control
2. TGF-β2
3. TGF-β2+sh-NC
4. TGF-β2+sh-KLF5

Fig5 D

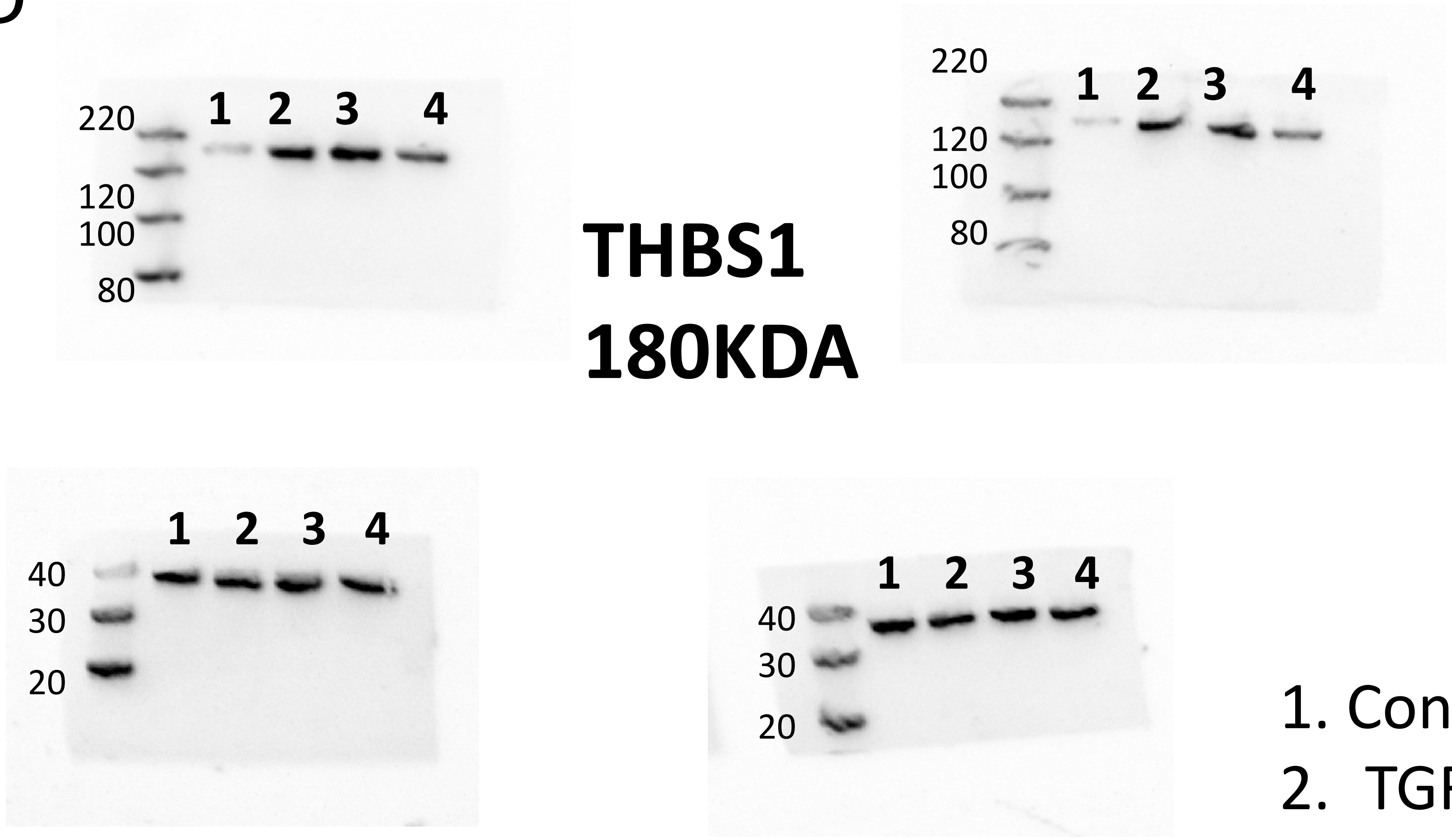

GAPDH
36KDA

1. Control
2. TGF-β2
3. TGF-β2+sh-NC
4. TGF-β2+sh-KLF5

Fig6 F

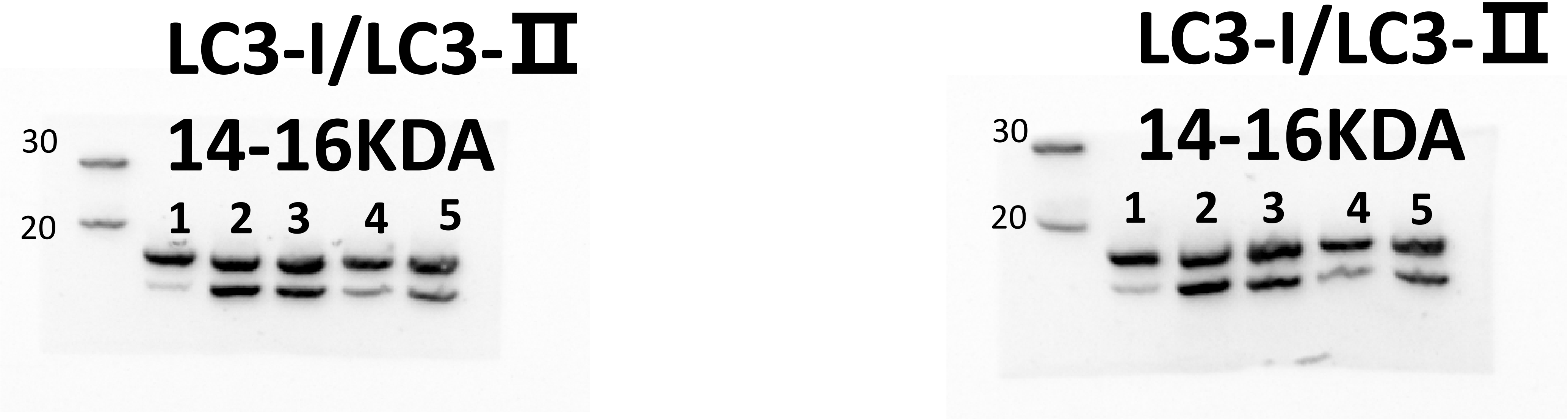

1. Control
2. TGF-β2
3. TGF-β2+sh-NC
4. TGF-β2+sh-KLF5
5. TGF-β2+sh-KLF5 +oe-THBS1

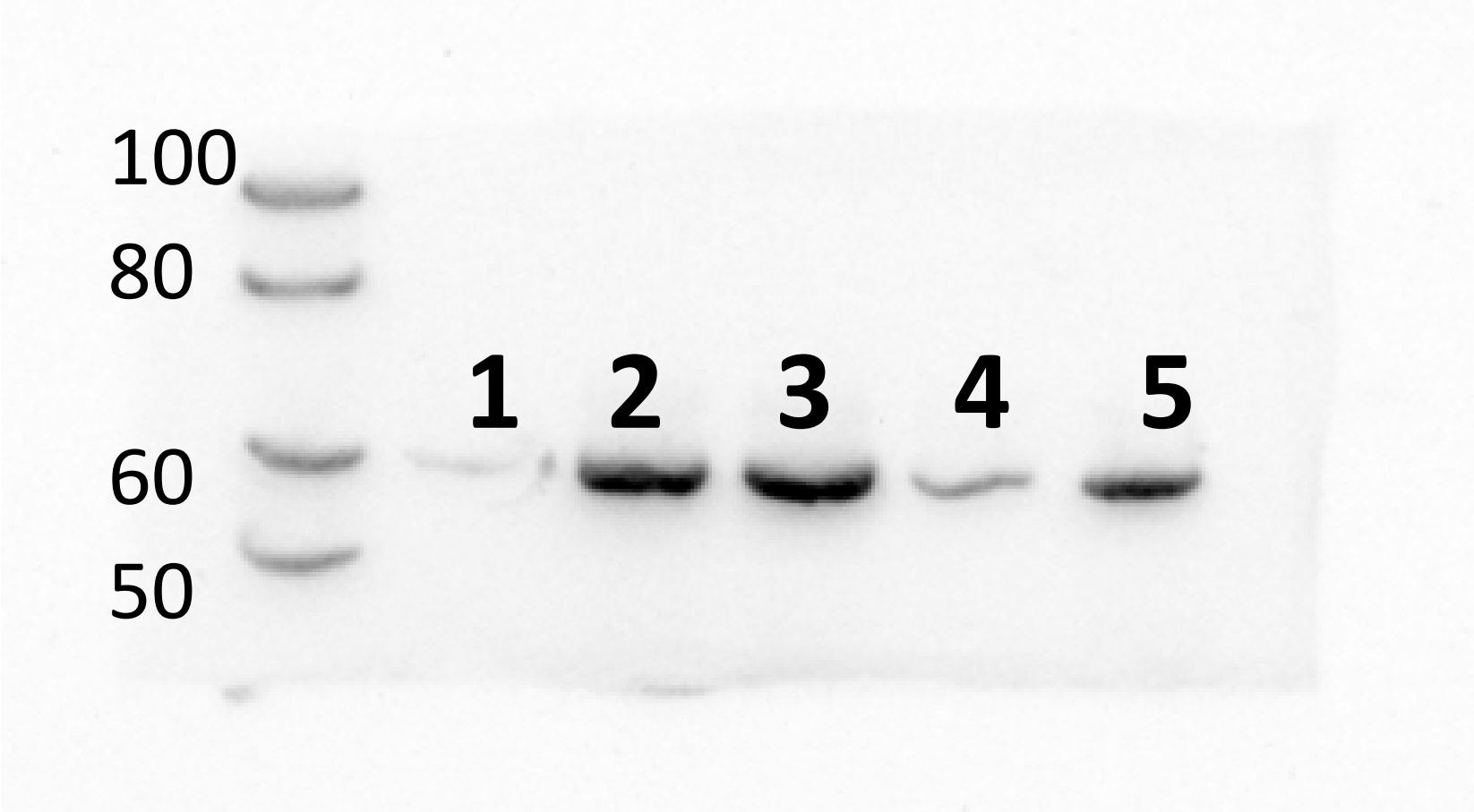

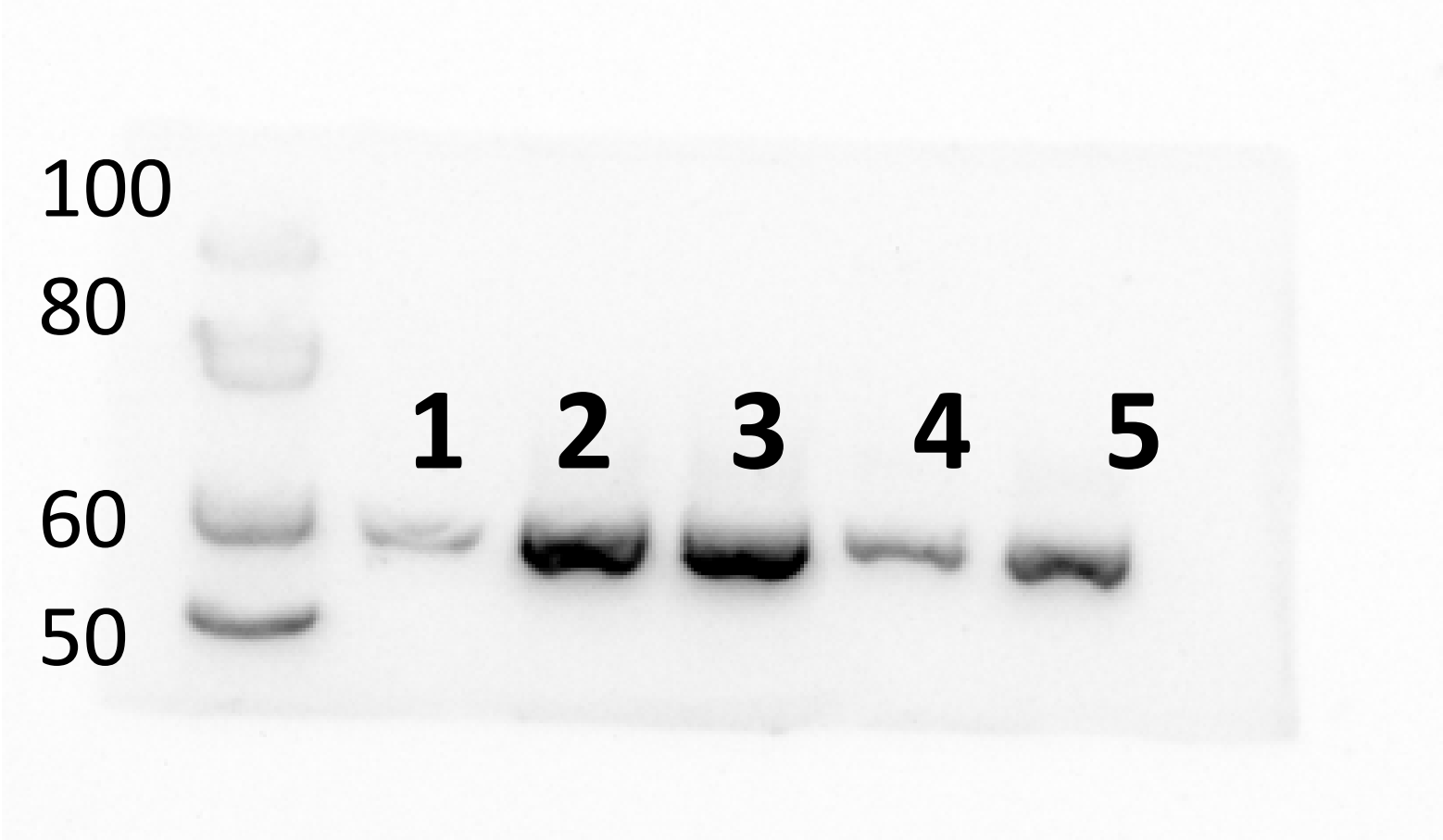

Fig6 F

Beclin 1
57 kDa

1. Control
2. TGF-β2
3. TGF-β2+sh-NC
4. TGF-β2+sh-KLF5
5. TGF-β2+sh-KLF5 +oe-THBS1

Fig6 F

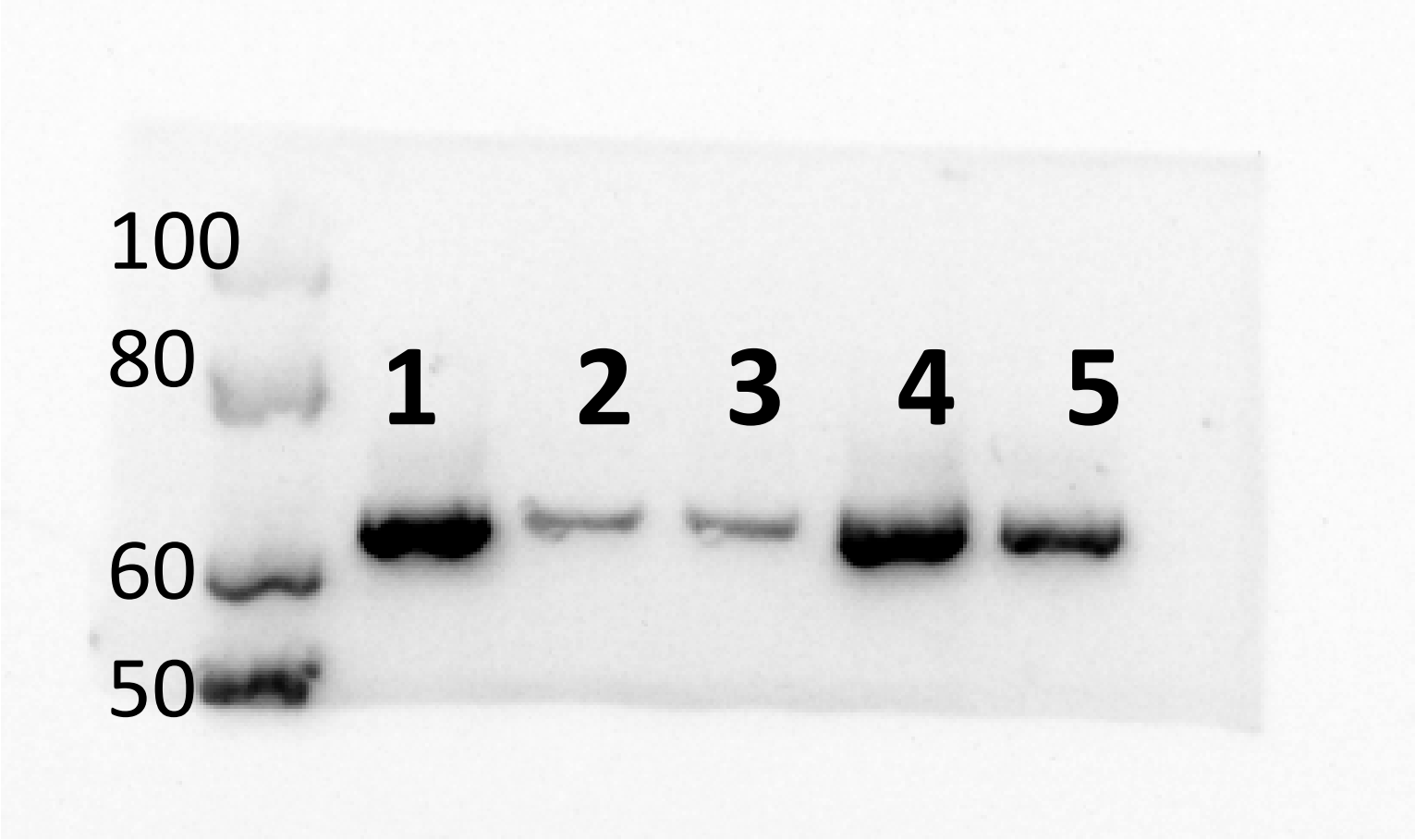

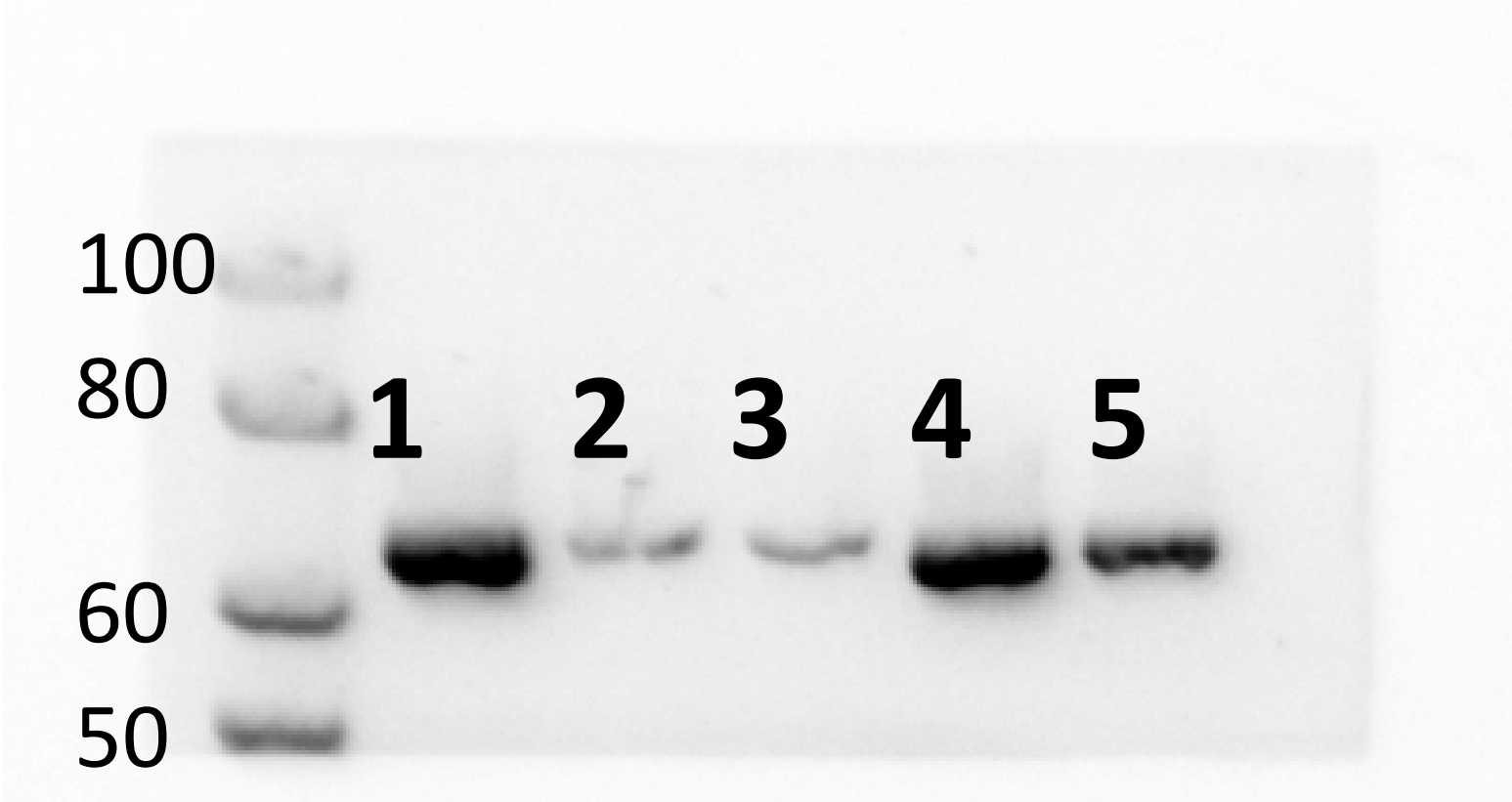

1. Control
2. TGF-β2
3. TGF-β2+sh-NC
4. TGF-β2+sh-KLF5
5. TGF-β2+sh-KLF5 +oe-THBS1

P62
62KDA

Fig6 F

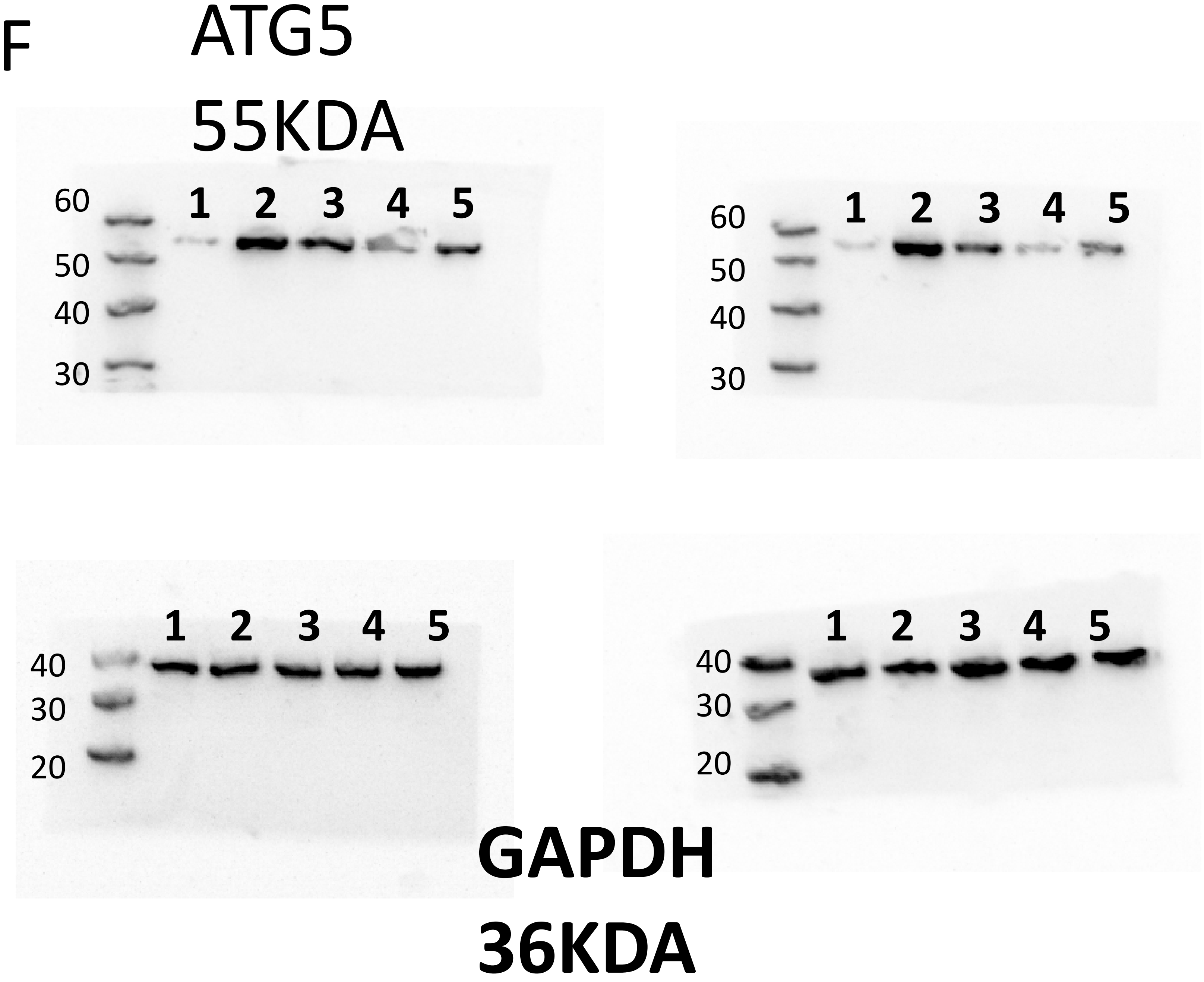

1. Control
2. TGF-β2
3. TGF-β2+sh-NC
4. TGF-β2+sh-KLF5
5. TGF-β2+sh-KLF5 +oe-THBS1

Fig6 G

COL-1

220KDA

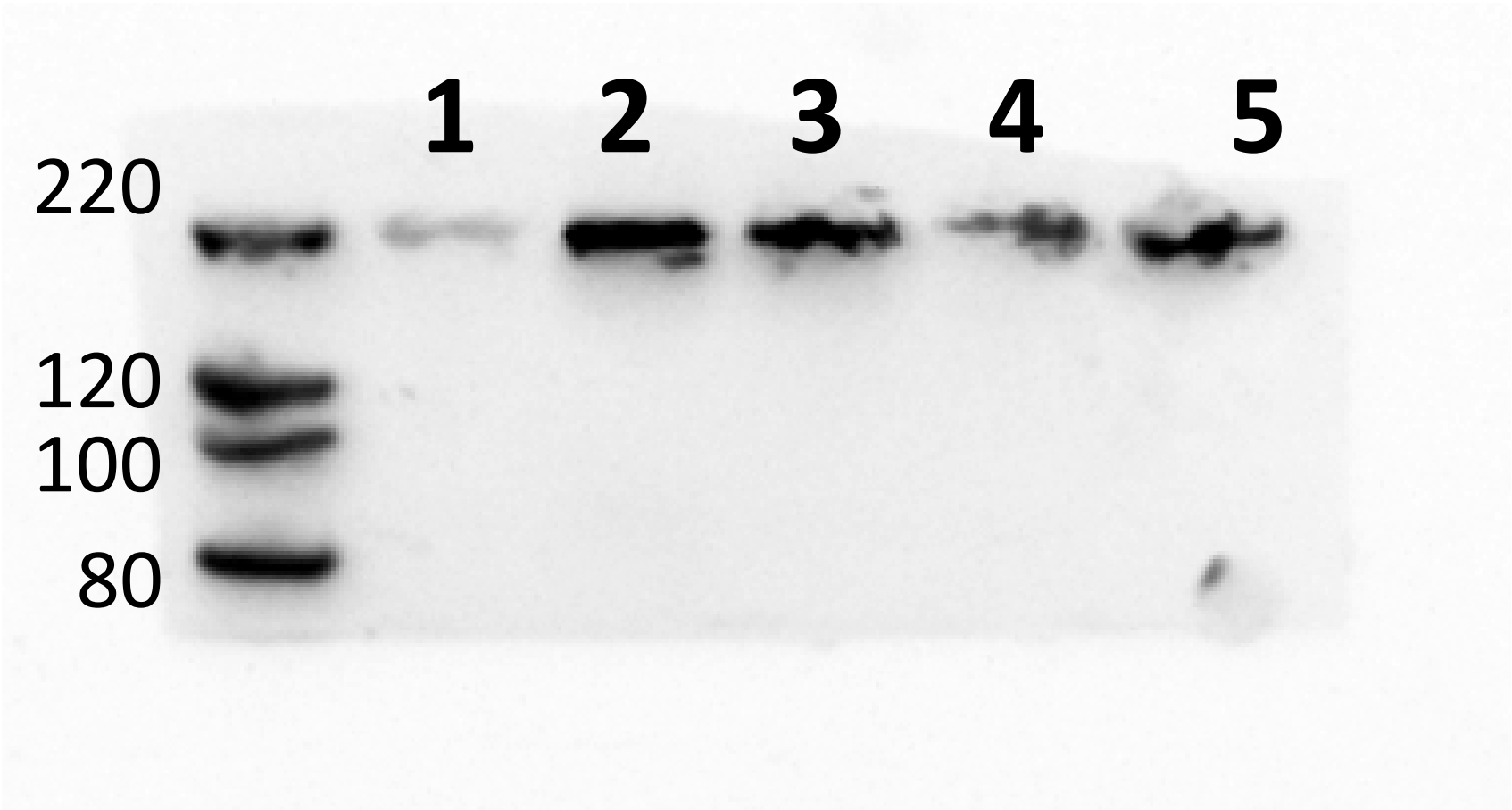

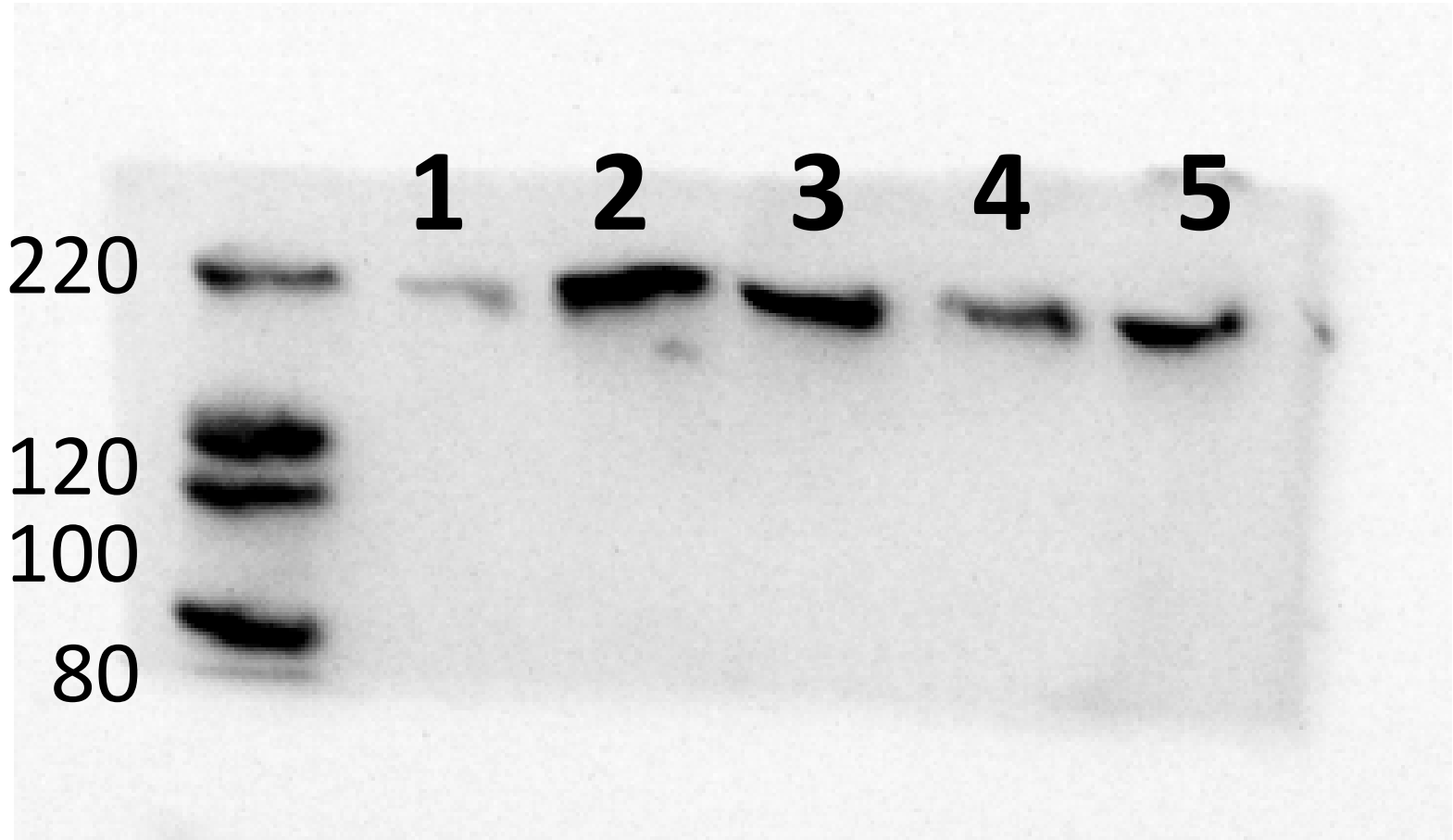

1. Control
2. TGF-β2
3. TGF-β2+sh-NC
4. TGF-β2+sh-KLF5
5. TGF-β2+sh-KLF5 +oe-THBS1

Fig6 G

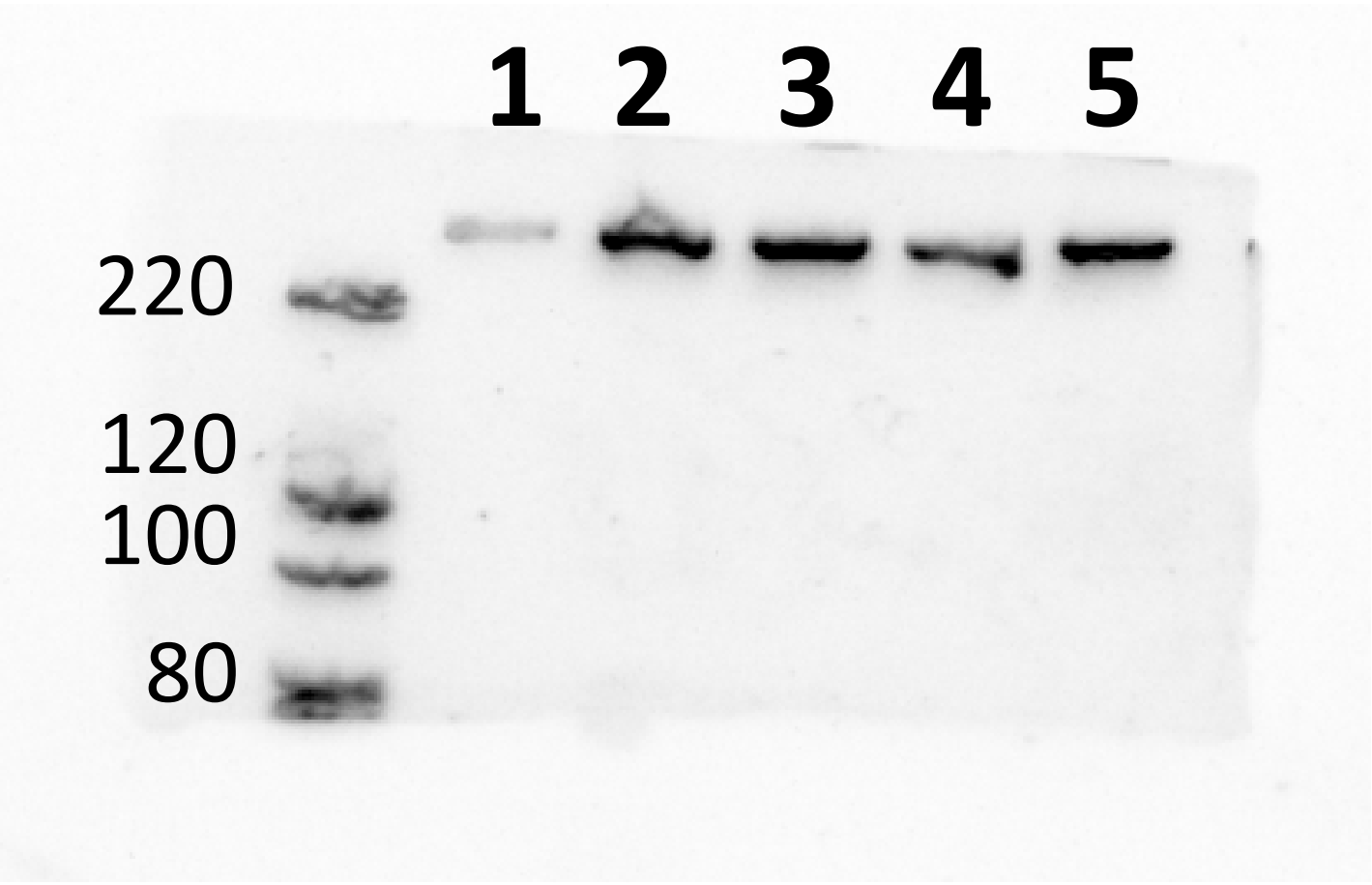

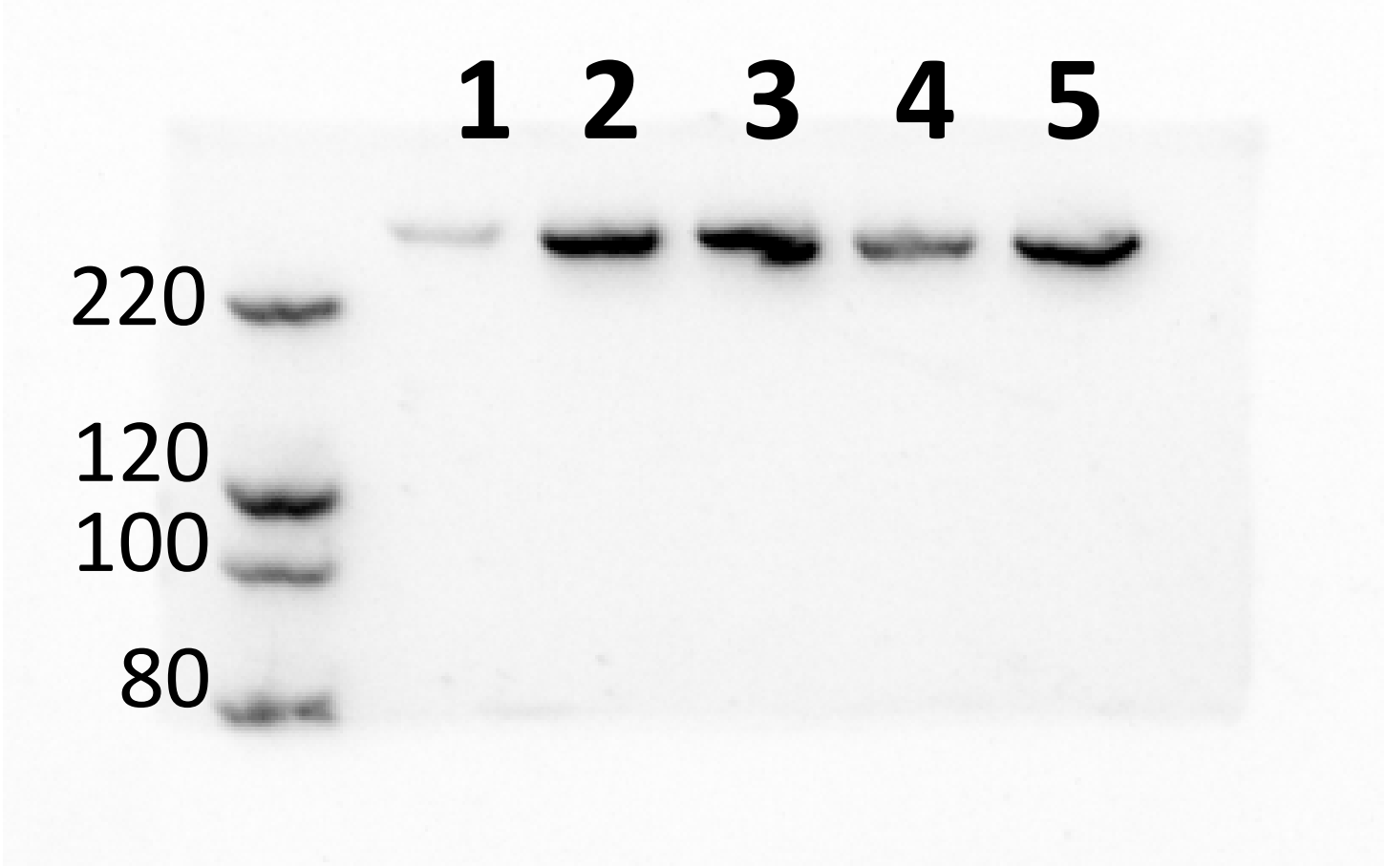

1. Control
2. TGF-β2
3. TGF-β2+sh-NC
4. TGF-β2+sh-KLF5
5. TGF-β2+sh-KLF5 +oe-THBS1

Fig6 G **α-SMA 42 kDa**

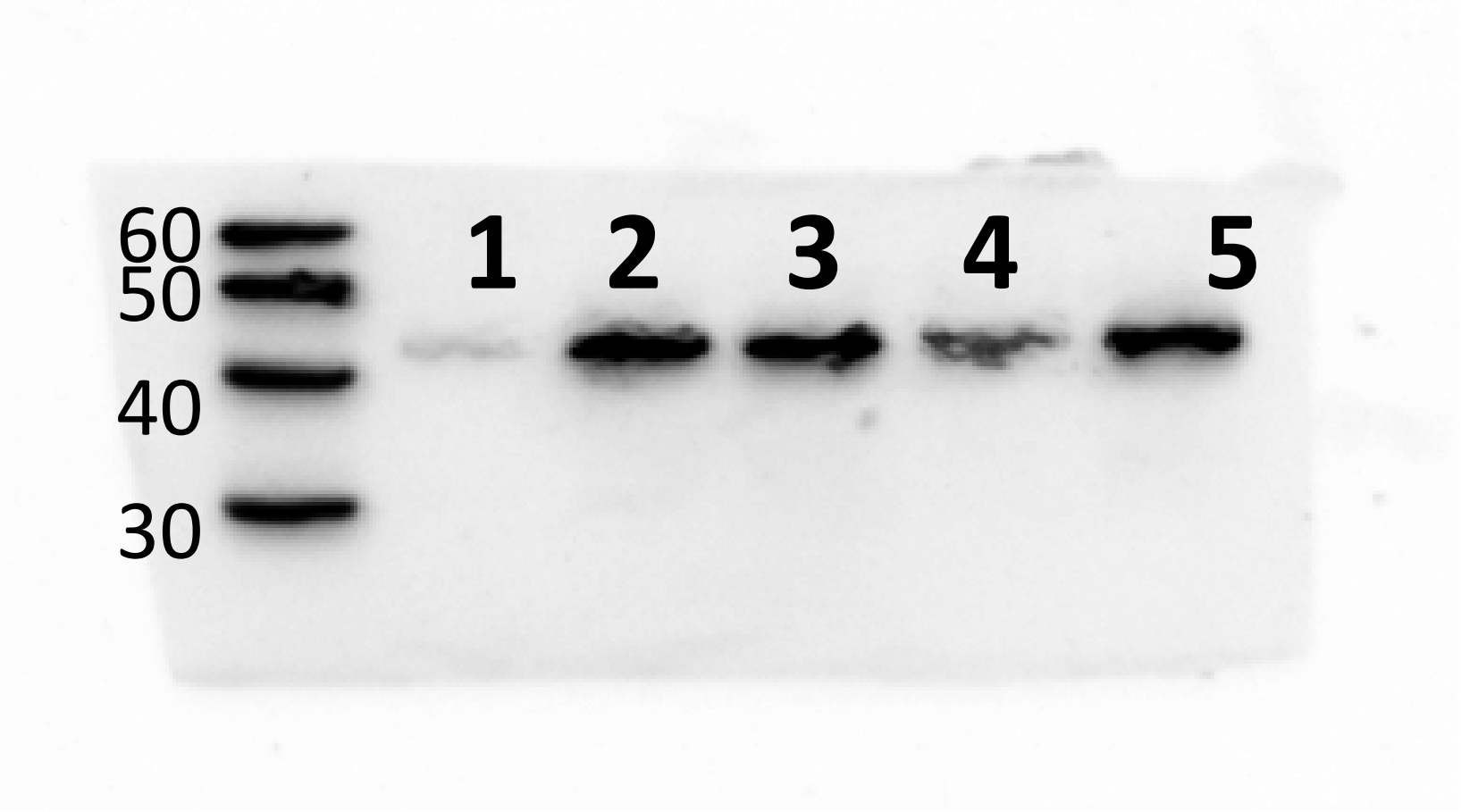

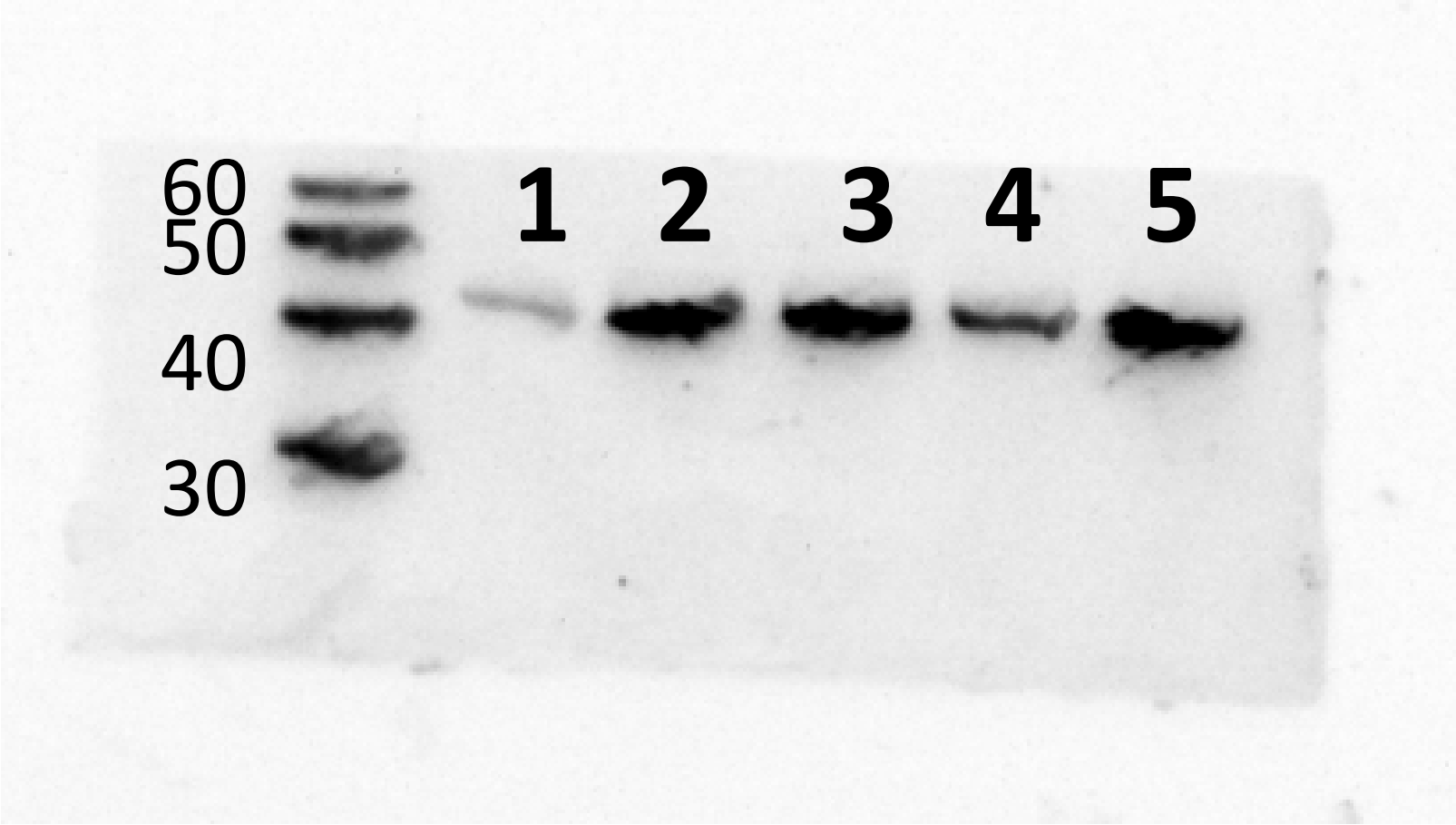

1. Control
2. TGF-β2
3. TGF-β2+sh-NC
4. TGF-β2+sh-KLF5
5. TGF-β2+sh-KLF5 +oe-THBS1

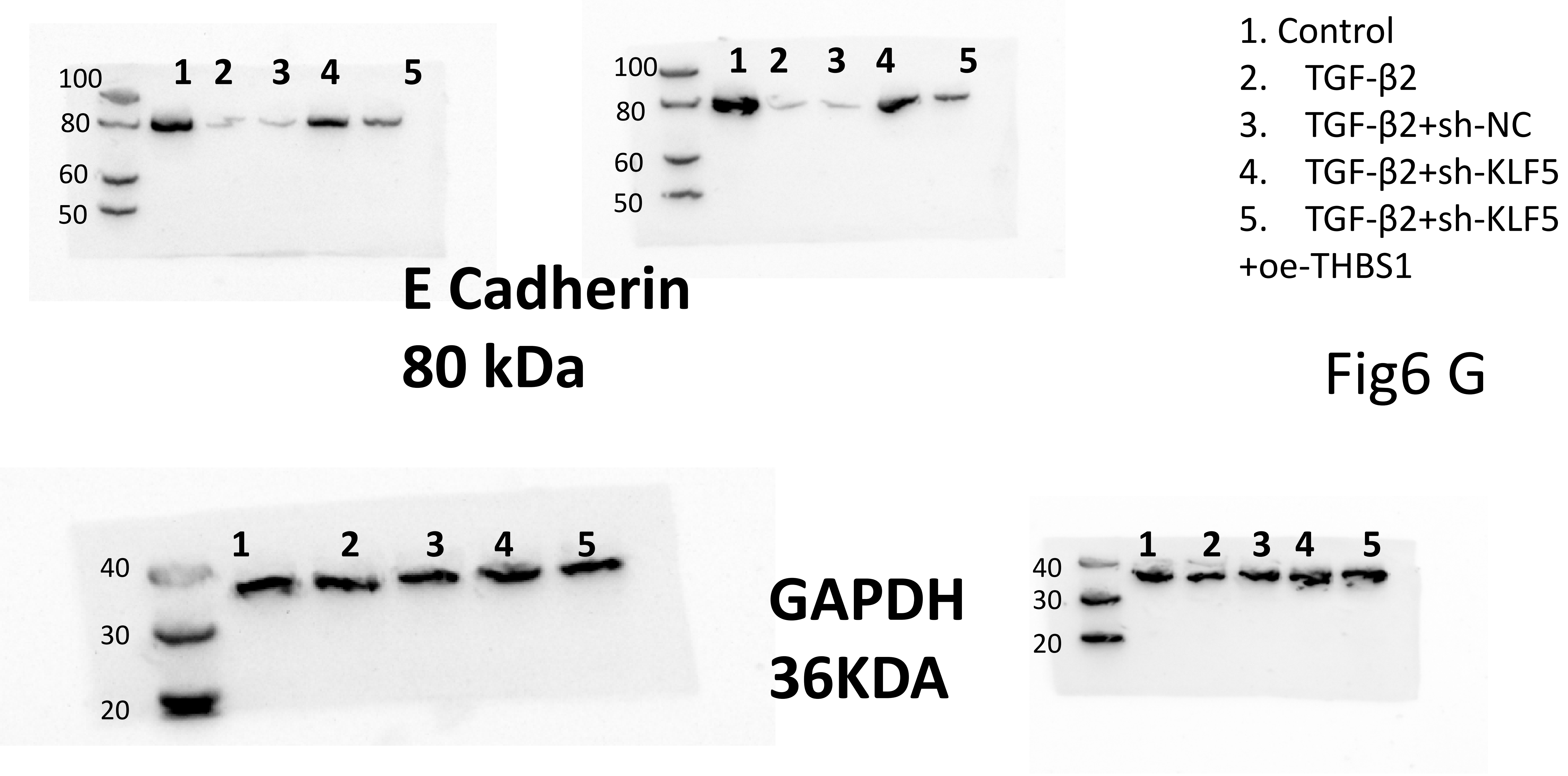
100
80
60
50
1 2 3 4 5
E Cadherin
80 kDa
1. Control
2. TGF-β2
3. TGF-β2+sh-NC
4. TGF-β2+sh-KLF5
5. TGF-β2+sh-KLF5
+oe-THBS1
40
30
20
GAPDH
36KDA

Fig6 G
